# Supplementary material for: Hepatic IR and IGF1R signaling govern distinct metabolic and carcinogenic processes upon PTEN deficiency in the liver
Source: JHEP Rep. 2024 Dec 19;7(4):101305. doi: 10.1016/j.jhepr.2024.101305 (PMC11925173; doi:10.1016/j.jhepr.2024.101305)
Supplement: Multimedia component 1 [file mmc1.pdf]

Hepatic IR and IGF1R signaling govern distinct metabolic and carcinogenic processes upon PTEN deficiency in the liver

Monika Gjorgjieva, Nicolas Calo, Cyril Sobolewski, Dorothea Portius, Jean-Luc Pitetti, Flavien Berthou, Anne-Sophie Ay, Marion Peyrou, Lucie Bourgoin, Christine Maeder, Margot Fournier, Marta Correia de Sousa, Etienne Delangre, Laurent Vinet, Xavier Montet, Christine Sempoux, Serge Nef, Michelangelo Foti

Table of contents

Supplementary figures……………………………………………………………………....2

Table S1…………………………………………………………………………….....24

Supplementary materials and methods………………………………………………….…25

Supplementary references…………………………………………………………...30

Supplementary figures

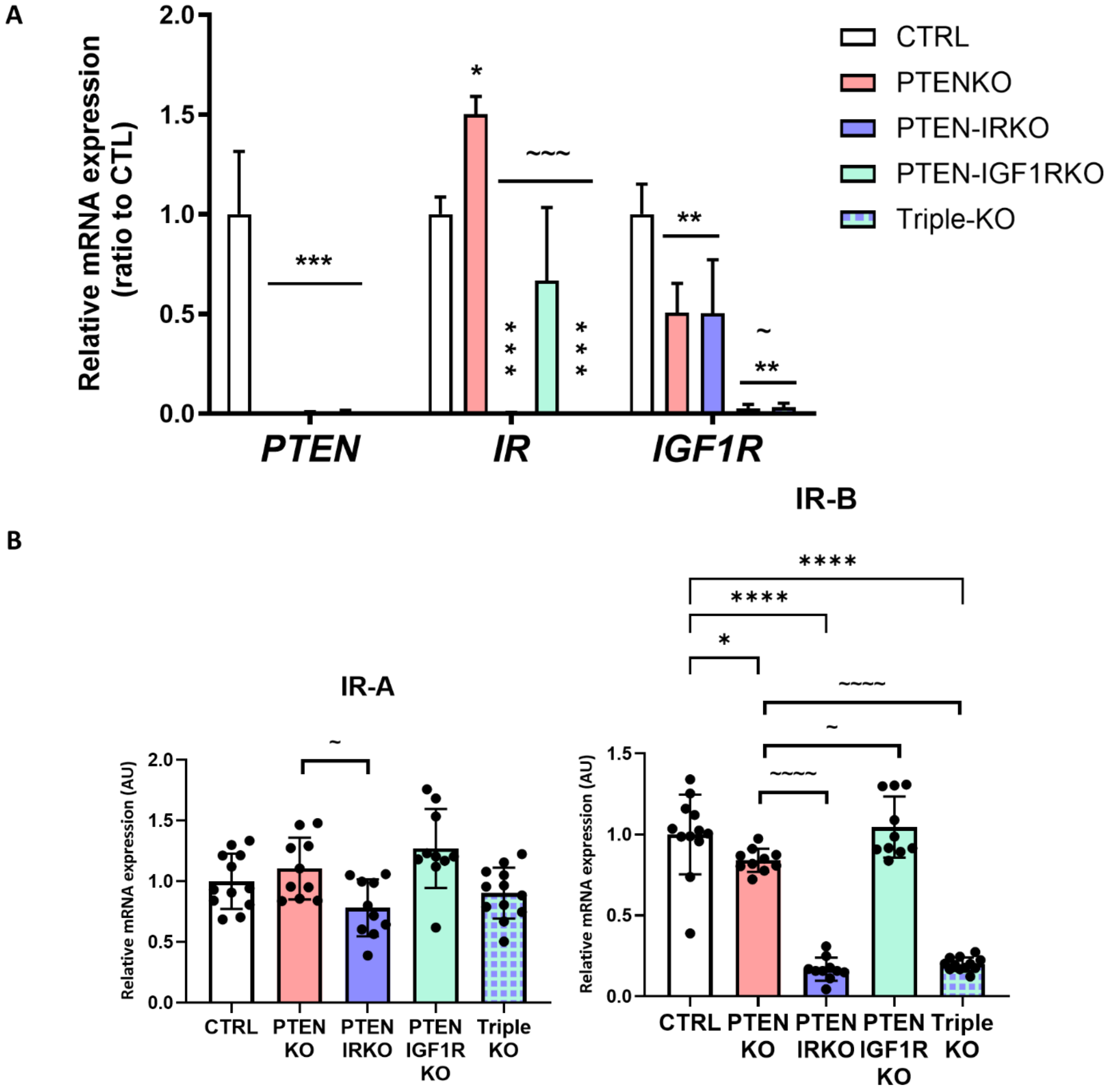

Fig. S1: Validation of hepatocyte-specific deletions of IR, IGF1R and PTEN in the different mutants.

(A) Specific genomic ablations of *Pten*, *Insr* and *Igf1r* were confirmed by RT-qPCR on isolated primary mouse hepatocytes. Values are mean ± SD of 3 mice per group. Primary mouse hepatocytes were isolated and seeded as previously described in Horie et al. [1]. After 24h, supernatant, protein and RNA were collected and flash frozen at -80°C (one-way ANOVA used for statistical analysis).

(B) Insulin receptor (IR) isoform A and B mRNA expression was investigated under fed conditions in the livers of CTRL, PTENKO, PTEN-IRKO, PTEN-IGF1RKO and triple-KO mice at 4 months of age *via* RT-qPCR. Values are mean +/- SD of 10-12 mice per group (2-way ANOVA used for statistical analysis).

Values were considered significant when compared to CTRL (*) or to PTENKO (~):
*/~ p≤ 0.05, **/~~ p≤ 0.01,***/~~~ p≤ 0.001 or ****/~~~~ p≤ 0.0001.

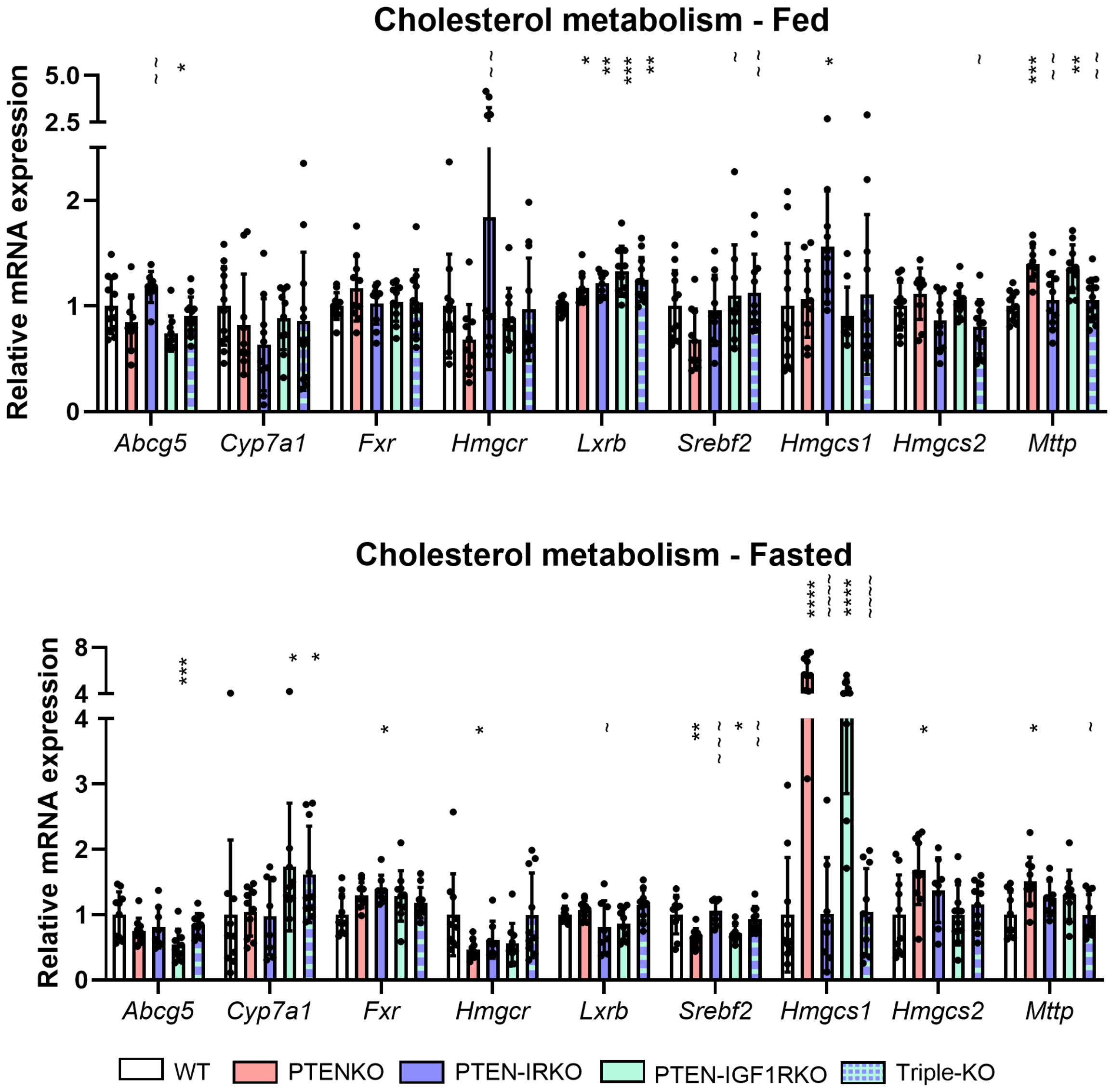

Fig. S2: Hepatic expression of key enzymes involved in cholesterol metabolism.

mRNA expressions of key genes involved in cholesterol metabolism (fed state: CTRL n=12, PTENKO n=10, PTEN-IRKO n=10, PTEN-IGF1RKO n=10, triple-KO n=12; fasted state: CTRL n=10, PTENKO n=10, PTEN-IRKO n=8, PTEN-IGF1RKO n=10, triple-KO n=10) in the livers of 4 months old mice under fed (upper panels) and overnight fasted conditions (bottom panels). Outliers were removed following ROUT (Q=1%) test and one-way ANOVA was performed. Values are represented as mean +/- SD. Values were considered significant when compared to CTRL (*) and to PTENKO (~):
*/~ p≤ 0.05, **/~~ p≤ 0.01,***/~~~ p≤ 0.001 or ****/~~~~ p≤ 0.0001.

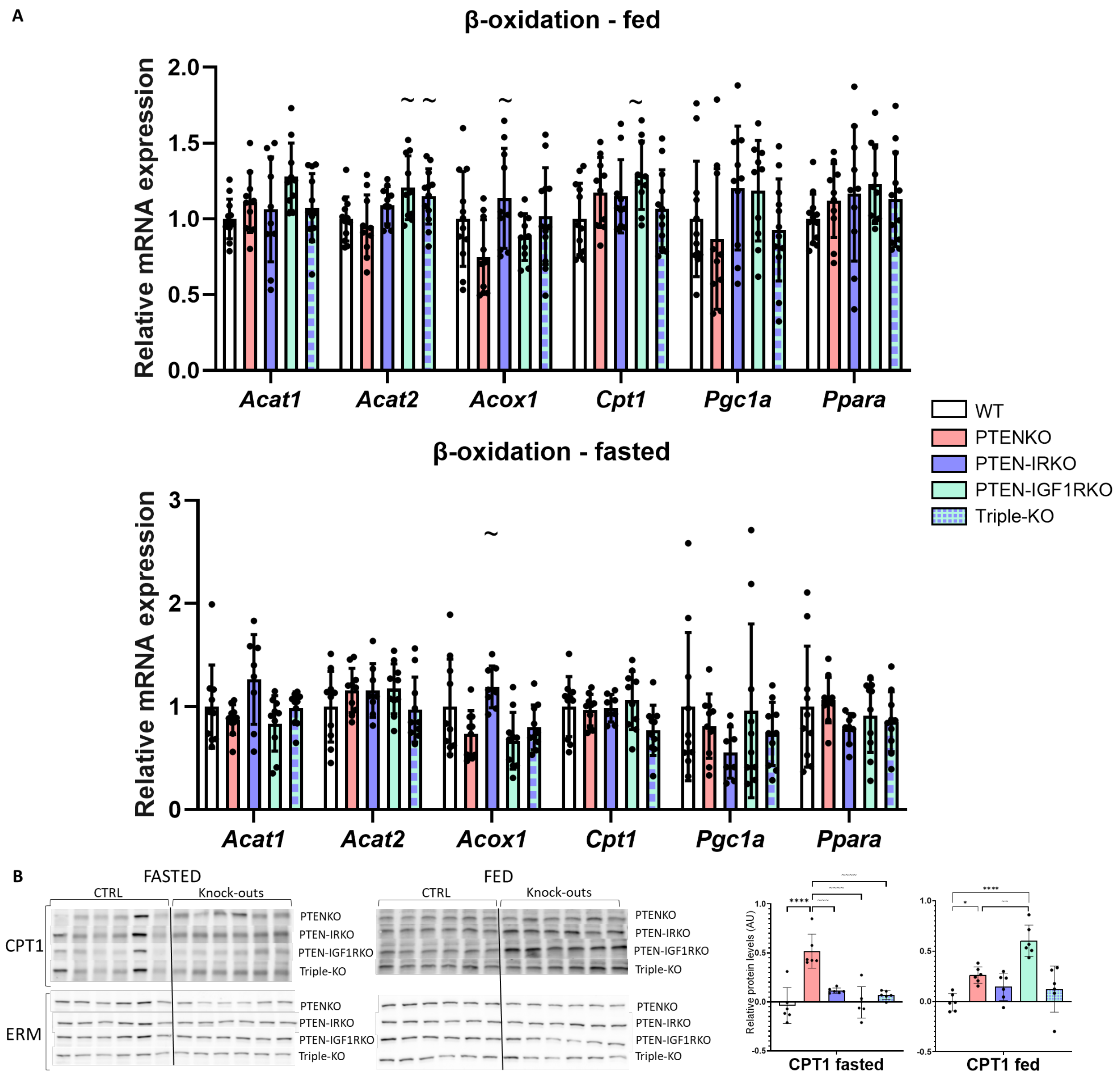

Fig. S3: Hepatic expression of key enzymes involved in β-oxidation.

(A) Hepatic β-oxidation was investigated under fasted or fed conditions in CTRL, PTENKO, PTEN-IRKO, PTEN-IGF1RKO and triple-KO mice at 4 months of age *via* RT-qPCR of *Acat1, Acat2, Acox1, Cpt1, Pgc1a* and *Ppara*. Values are represented as mean ± SD of 8-12 mice per group.

(B) CPT1 protein levels were determined *via* Western blot in CTRL, PTENKO, PTEN-IRKO, PTEN-IGF1RKO and triple-KO livers under fasted or fed conditions. ERM was used as standardization protein (run at the same time as Figure 2B – same normalization blot, 6 mice per group). Values are represented as mean ± SD.

Outliers were removed following ROUT (Q=1%) test and one-way ANOVA was performed. Values were considered significant when compared to CTRL (*) and to PTENKO (~):
*/~ p≤ 0.05, **/~~ p≤ 0.01, ***/~~~ p≤ 0.001 or ****/~~~~ p≤ 0.0001.

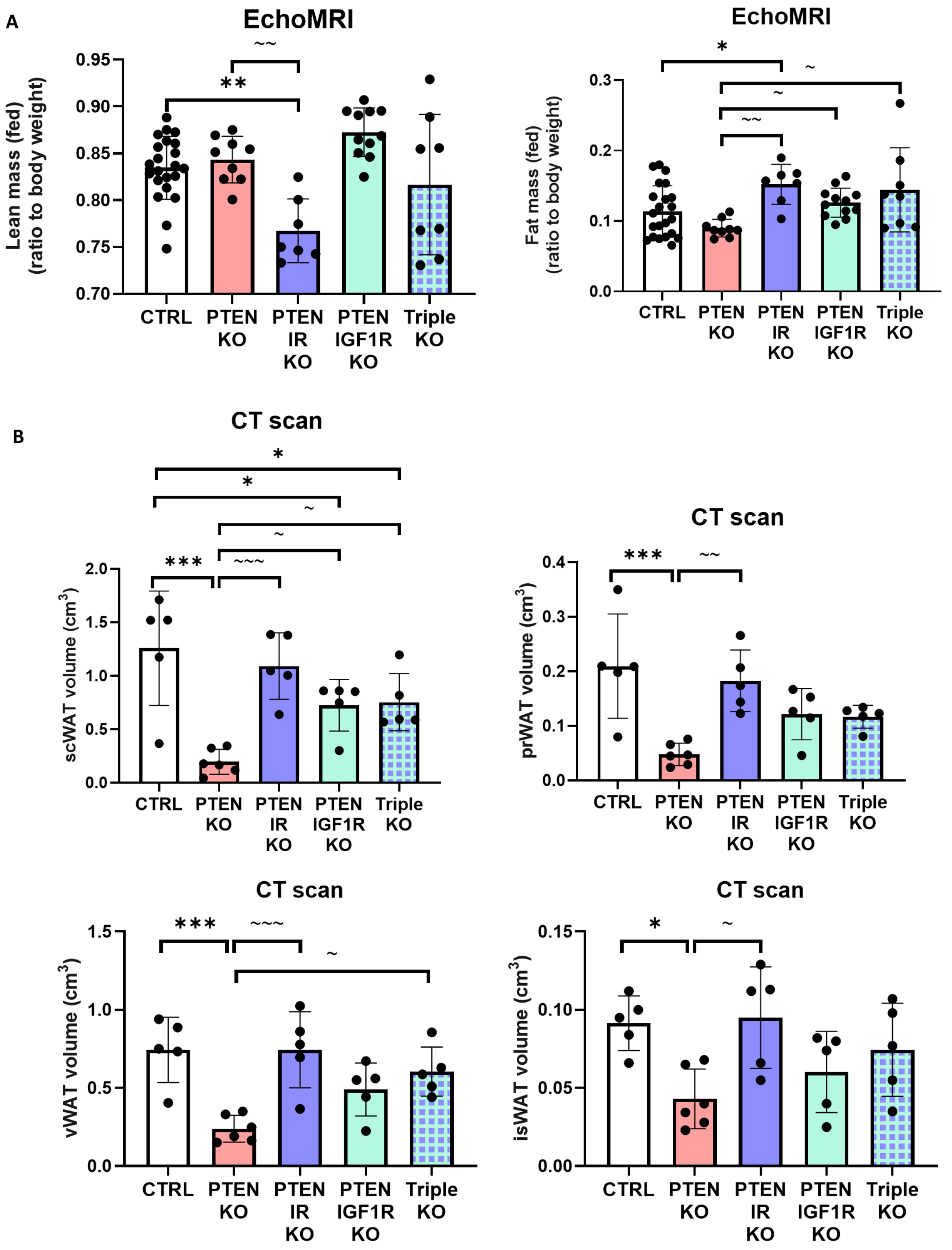

Fig. S4: Echo-MRI analysis and CT-scan analysis of different white adipose tissue depots.

(A) Total lean mass and fat mass determined through Echo-MRI.

(B) scWAT – subcutaneous white adipose tissue, prWAT – perirenal white adipose tissue, vWAT – visceral white adipose tissue and isWAT – inguinal subcutaneous white adipose tissue volumes in CTRL, PTENKO, PTEN-IRKO, PTEN-IFG1RKO and PTEN-IR-IFG1RKO (triple-KO) mice under fed conditions determined through CT-scan (n=5-6/group).

Outliers were removed following ROUT (Q=1%) test and one-way ANOVA was performed. Values are represented as mean ±SD and were considered significant when compared to CTRL (*) and to PTENKO (~):
*/~ p≤ 0.05, **/~~ p≤ 0.01, ***/~~~ p≤ 0.001 or ****/~~~~ p≤ 0.0001.

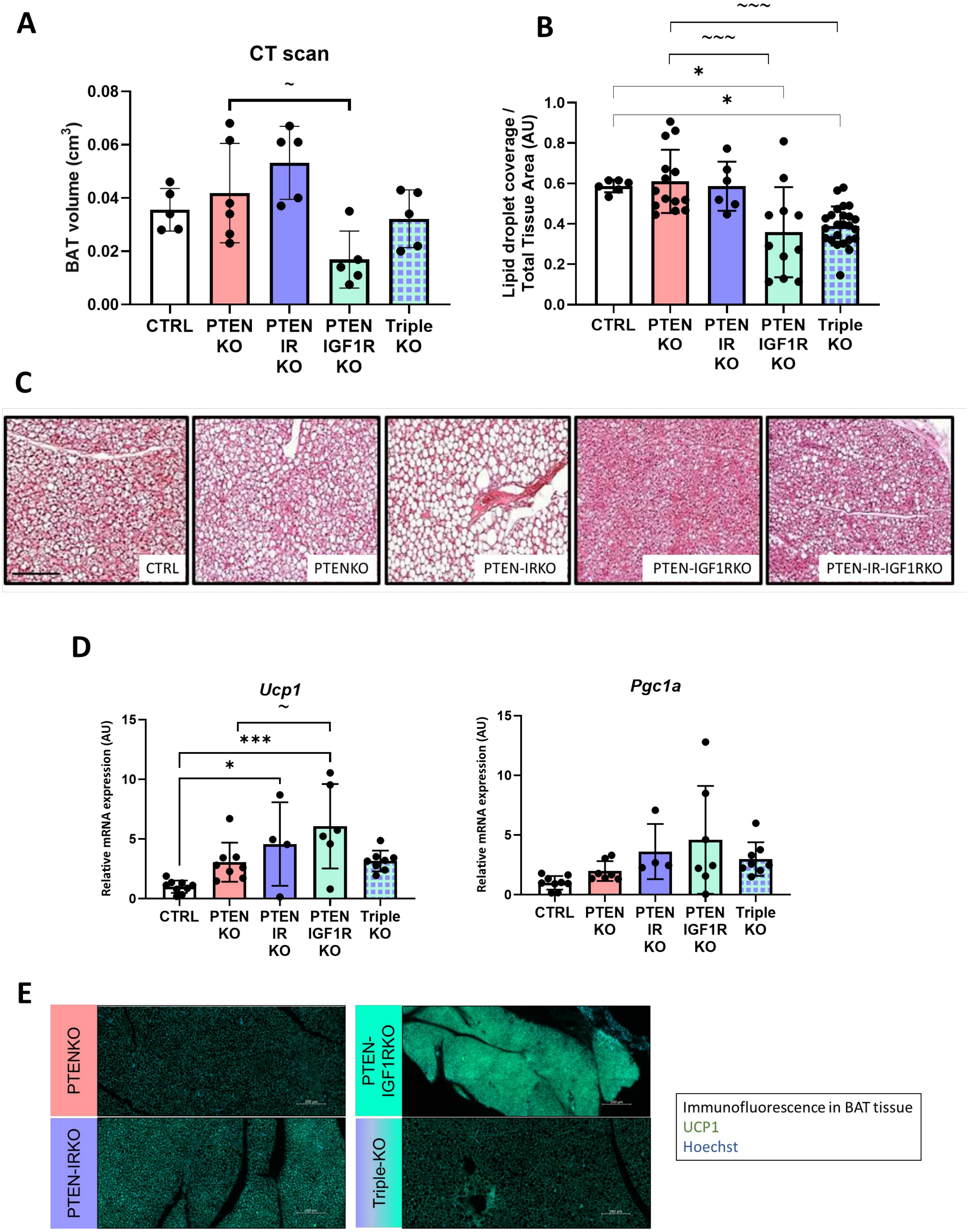

Fig. S5: Analysis of the brown adipose tissue in the different mutants.

(A) Volume of interscapular brown adipose tissue (BAT) as determined by CT-scan analyses in CTRL (n=5), PTENKO (n=6), PTEN-IRKO (n=5), PTEN-IGF1RKO (n=5), and triple-KO (n=5) mice.

(B) Quantification of lipid droplet area over total BAT area in CTRL (n=6), PTENKO (n=14), PTEN-IRKO (n=6), PTEN-IGF1RKO (n=11), and triple-KO (n=24) mice. H&E stained sections from BAT were scanned using AxioScan and analyzed using the QuPath software. Following tissue manual detection, the pixel classification tool was used to classify positive areas within the established ROI (channel: red; prefilter: gaussian; smoothing sigma: 2). Lipid droplets were considered as empty spaces (negative areas) within the region of interest (ROI) and calculated based on the following: 1- (positive area/total tissue area).

(C) Representative histological sections (H&E staining) of BAT sections.

(D) mRNA expressions of UCP1 and PGC1a (CTRL n=9, PTENKO n=8, PTEN-IRKO n=4, PTEN-IGF1RKO n=6, triple-KO n=8) analyzed through RT-qPCR.

(E) Representative images of UCP1 immunofluorescence in the BAT of CTRL, PTENKO, PTEN-IRKO, PTEN-IGF1RKO, and triple-KO mice (green-UCP1, blue – Hoechst).

Outliers were removed following ROUT (Q=1%) test and one-way ANOVA was performed. Values were considered significant when compared to CTRL (*) and to PTENKO (~):
*/~ p≤ 0.05, **/~~ p≤ 0.01, ***/~~~ p≤ 0.001 or ****/~~~~ p≤ 0.0001.

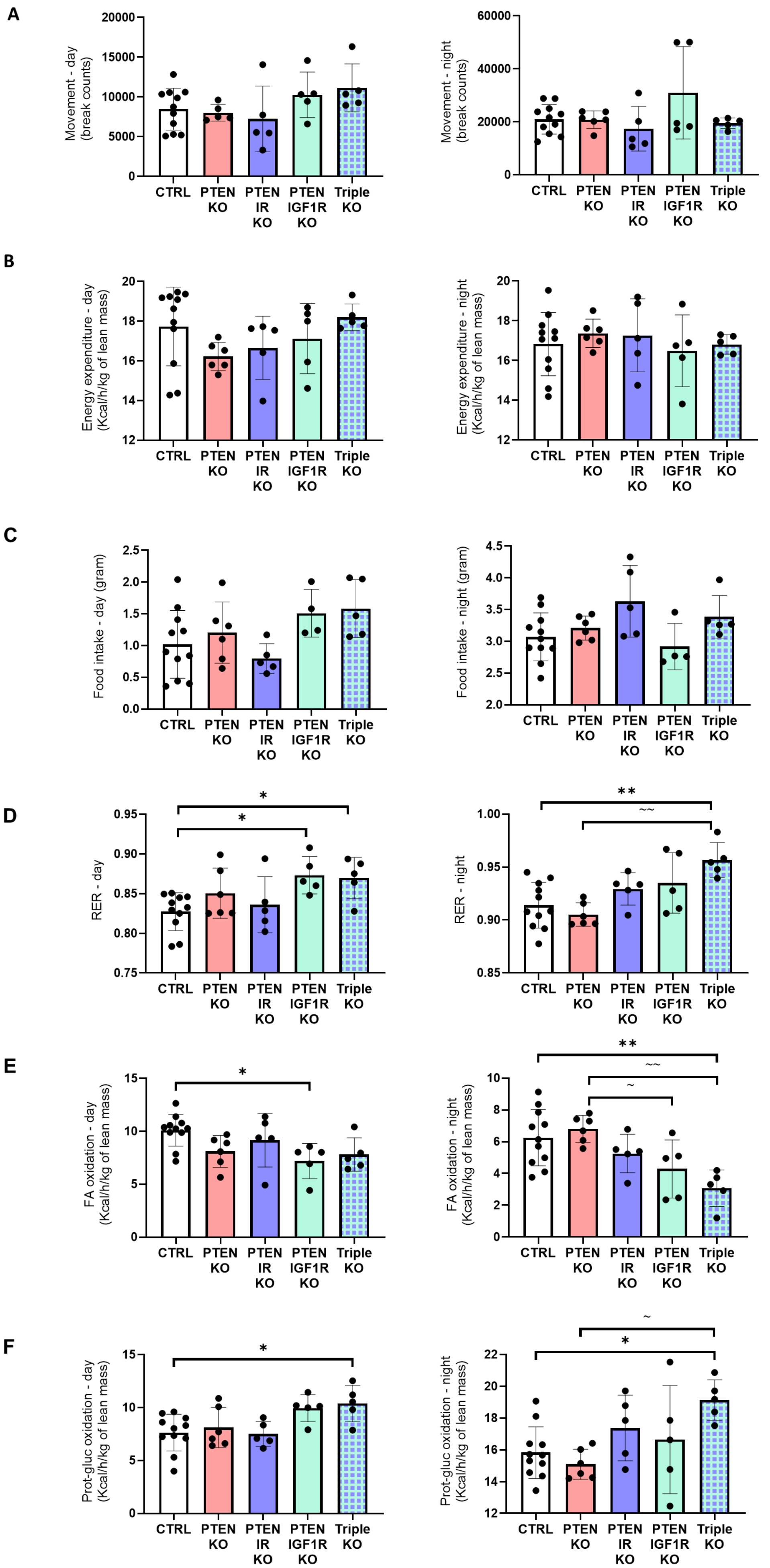
A
Movement - day (break counts)
20000
15000
10000
5000
0
Movement - night (break counts)
60000
40000
20000
0
B
Energy expenditure - day (Kcal/h/kg of lean mass)
Energy expenditure - night (Kcal/h/kg of lean mass)
20
18
16
14
12
C
Food intake - day (gram)
2.5
2.0
1.5
1.0
0.5
0.0
Food intake - night (gram)
4.5
4.0
3.5
3.0
2.5
2.0
D
RER - day
0.95
0.90
0.85
0.80
0.75
*
*
RER - night
1.00
0.95
0.90
0.85
**
~~
E
FA oxidation - day (Kcal/h/kg of lean mass)
15
10
5
0
*
FA oxidation - night (Kcal/h/kg of lean mass)
10
8
6
4
2
0
**
~~
~
F
Prot-gluc oxidation - day (Kcal/h/kg of lean mass)
15
10
5
0
*
Prot-gluc oxidation - night (Kcal/h/kg of lean mass)
22
20
18
16
14
12
~
*
CTRL
PTEN KO
PTEN IR KO
PTEN IGF1R KO
Triple KO

Fig. S6: Metabolic phenotyping of the different mutants.

(A) Physical activity - movement, (B) energy expenditure, (C) food consumption, (D) Respiratory Exchange Ratio (RER) (E) fatty acid consumption and (F) protein and glucose oxidation were recorded for 24 hours using Labmaster metabolic cages. Graphs represent values obtained during the day (left panels) vs during the night (right panels). Values are mean (± SD) of 5-11 mice per group. Outliers were removed following ROUT (Q=1%) test and one-way ANOVA was performed. Values were considered significant when compared to CTRL (*) and to PTENKO (~):
*/~ $p \leq 0.05$, **/~~ $p \leq 0.01$, ***/~~~ $p \leq 0.001$ or ****/~~~~ $p \leq 0.0001$.

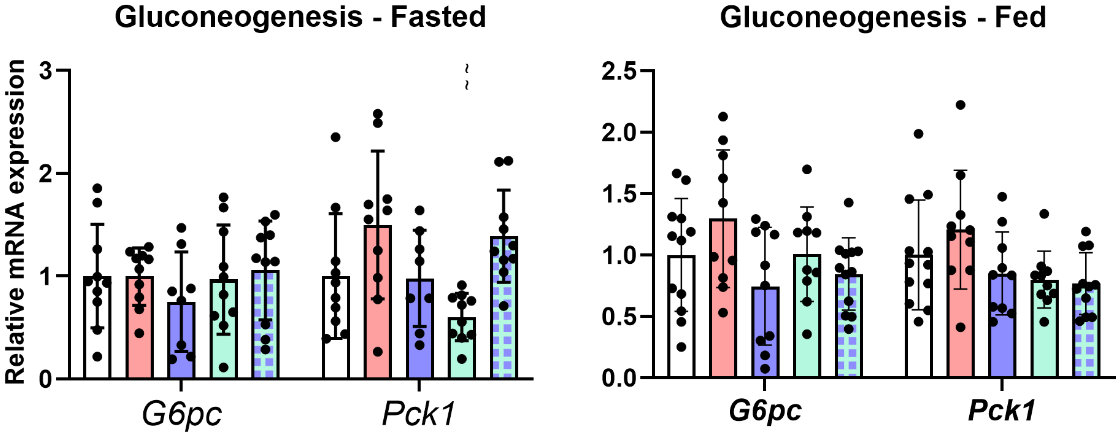

Fig. S7: Hepatic gluconeogenic gene expression in the different mutants in fasted and fed conditions.

Hepatic gluconeogenic gene expression levels were investigated in fasted conditions (left panel, CTRL n=10, PTENKO n=10, PTEN-IRKO n=8, PTEN-IGF1RKO n=10, and triple-KO n=10), as well as in fed conditions (right panel, CTRL n=12, PTENKO n=10, PTEN-IRKO n=10, PTEN-IGF1RKO n=10, and triple-KO n=12), at 4 months of age *via* RT-qPCR. Values are represented as fold-change to CTRL (± SD). Outliers were removed following ROUT (Q=1%) test and one-way ANOVA was performed. Values were considered significant when compared to CTRL (*) and to PTENKO (~):
*/~ p≤ 0.05, **/~~ p≤ 0.01, ***/~~~ p≤ 0.001 or ****/~~~~ p≤ 0.0001.

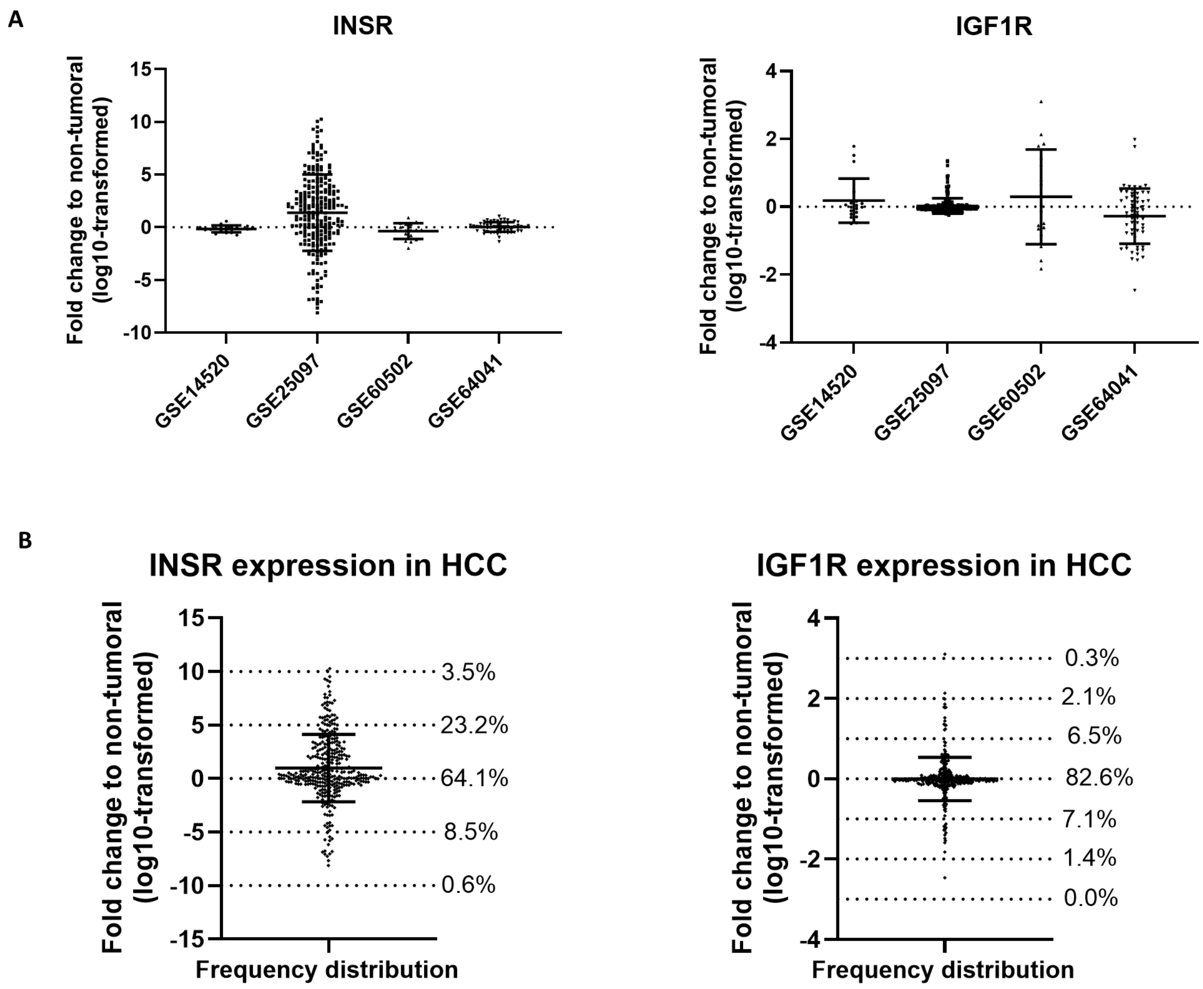

Fig. S8: INSR and IGF1R mRNA expression in HCC *versus* non-tumoral liver.

(A) The mRNA expression levels of INSR and IGF1R were determined in 4 different GEOdatasets of HCC patients (GSE14520, n=19, GSE25097, n=243, GSE60502, n=18, GSE64041, n=60) and the fold change in expression between HCC and non-tumoral biopsy was determined for each patient. Expression data are presented as log10 transformed.

(B) The data from the 4 GEOdatasets were pooled together and frequency distribution was calculated (INSR: relative frequency percentages, bin=5 [-10,10]; IGF1R: relative frequency-percentages, bin=1 [-3,3].

Data is represented as mean+/-SD.

	GS follow link to MSigDB	GS DETAILS	SIZE	ES	NES	NOM p-val	FDR q-val	FWER p-val	RANK AT MAX	LEADING EDGE
1	HALLMARK_BILE_ACID_METABOLISM	Details ...	112	-0.78	-1.69	0.000	0.020	0.031	1512	tags=50%, list=7%, signal=54%
2	HALLMARK_FATTY_ACID_METABOLISM	Details ...	153	-0.70	-1.66	0.002	0.021	0.060	2118	tags=57%, list=10%, signal=63%
3	HALLMARK_PEROXISOME	Details ...	101	-0.65	-1.58	0.000	0.044	0.149	2026	tags=48%, list=10%, signal=52%
4	HALLMARK_XENOBIOTIC_METABOLISM	Details ...	196	-0.70	-1.52	0.010	0.073	0.286	1442	tags=49%, list=7%, signal=52%
5	HALLMARK_ADIPOGENESIS	Details ...	193	-0.50	-1.40	0.063	0.166	0.533	2525	tags=39%, list=12%, signal=45%
6	HALLMARK_OXIDATIVE_PHOSPHORYLATION	Details ...	194	-0.56	-1.40	0.107	0.141	0.537	2492	tags=35%, list=12%, signal=40%
7	HALLMARK_COAGULATION	Details ...	136	-0.56	-1.31	0.102	0.211	0.710	1285	tags=34%, list=6%, signal=36%
8	HALLMARK_INTERFERON_ALPHA_RESPONSE	Details ...	97	-0.58	-1.23	0.253	0.287	0.844	2544	tags=48%, list=12%, signal=55%
9	HALLMARK_KRAS_SIGNALING_DN	Details ...	192	-0.32	-1.23	0.128	0.258	0.847	3081	tags=19%, list=15%, signal=22%
10	HALLMARK_PANCREAS_BETA_CELLS	Details ...	39	-0.38	-1.20	0.211	0.269	0.882	880	tags=10%, list=4%, signal=11%
11	HALLMARK_ANDROGEN_RESPONSE	Details ...	97	-0.39	-1.17	0.153	0.290	0.909	1813	tags=28%, list=9%, signal=30%
12	HALLMARK_HEME_METABOLISM	Details ...	194	-0.30	-1.03	0.363	0.461	0.976	2899	tags=24%, list=14%, signal=27%
13	HALLMARK_COMPLEMENT	Details ...	194	-0.36	-1.02	0.427	0.456	0.983	2234	tags=24%, list=11%, signal=27%
14	HALLMARK_INTERFERON_GAMMA_RESPONSE	Details ...	200	-0.42	-1.00	0.456	0.443	0.984	1968	tags=28%, list=10%, signal=31%
15	HALLMARK_CHOLESTEROL_HOMEOSTASIS	Details ...	73	-0.41	-0.99	0.457	0.439	0.990	1492	tags=27%, list=7%, signal=29%

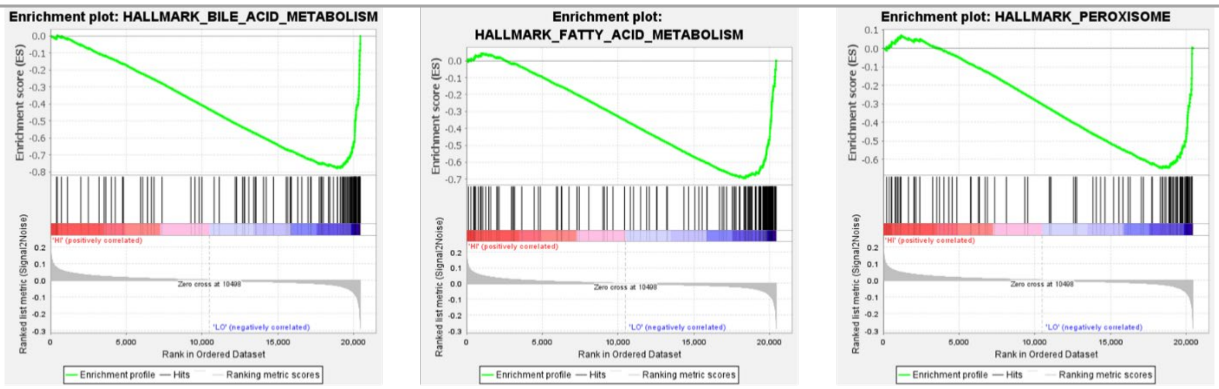

Gene sets enriched in phenotype LOW IGF1R

Fig. S9: Gene set enrichment analysis in HCC.

Gene set enrichment analysis was performed in the GSE36376 dataset containing expression analysis from human HCC (240 human HCC samples, segregated between the 20% most expressing *IGF1R* and the rest 80% of the samples). Enrichment was considered as significant if FDR q-value < 0.05. The three significantly enriched processes in low-IGF1R are highlighted in blue in the table and the enrichment plots are represented below.

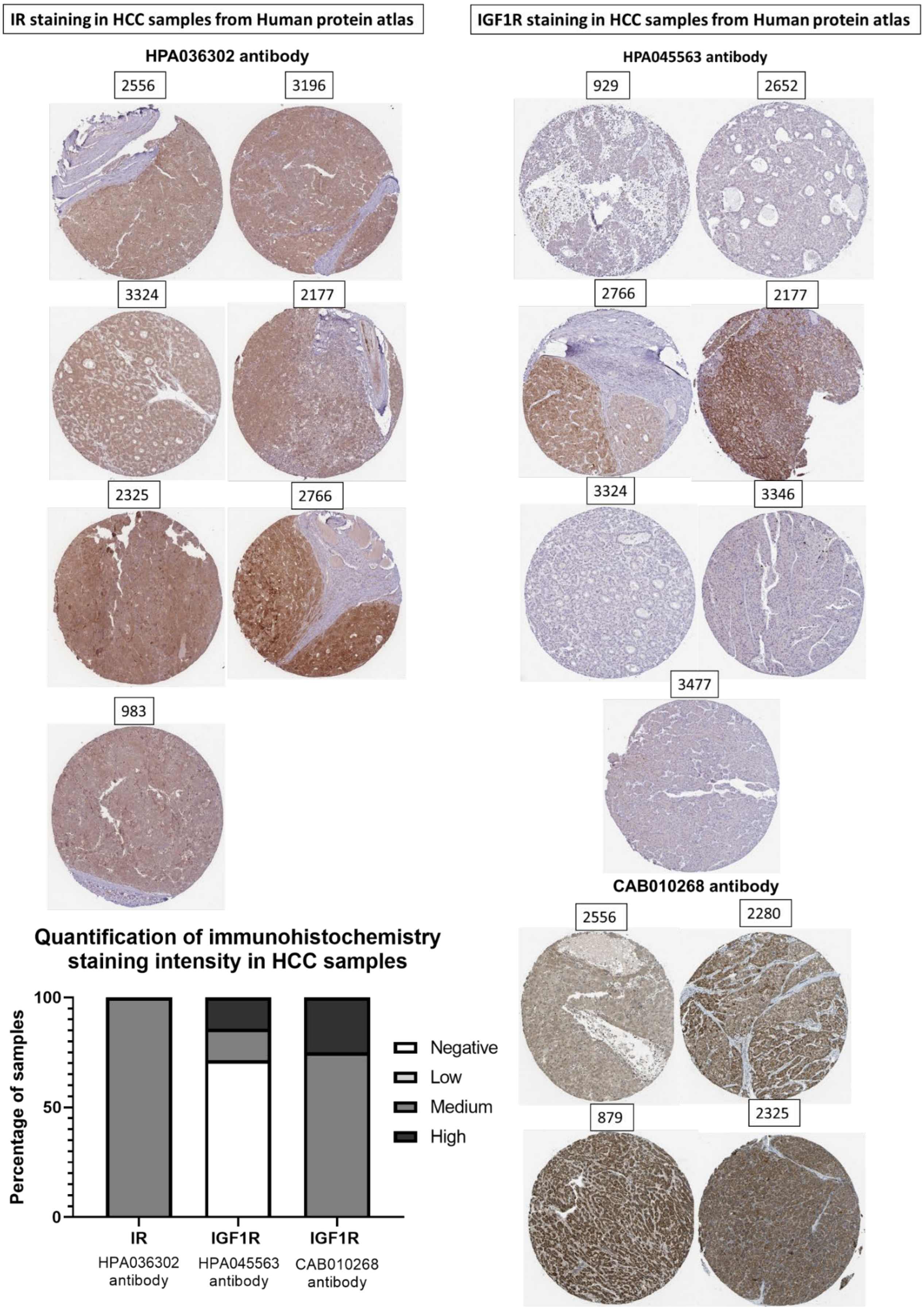
IR staining in HCC samples from Human protein atlas
HPA036302 antibody
2556
3196
3324
2177
2325
2766
983
IGF1R staining in HCC samples from Human protein atlas
HPA045563 antibody
929
2652
2766
2177
3324
3346
3477
CAB010268 antibody
2556
2280
879
2325
Quantification of immunohistochemistry staining intensity in HCC samples
Percentage of samples
100
50
0
Negative
Low
Medium
High
IR
HPA036302 antibody
IGF1R
HPA045563 antibody
IGF1R
CAB010268 antibody

Fig. S10: Immunohistochemistry of IR and IGF1R in HCC samples from the Human Protein Atlas.

Images for IR or IGF1R immunohistochemistry staining in biopsies from HCC patients were obtained from the *Human Protein Atlas* database (proteinatlas.org). The ID for each patient is indicated above the immunohistochemistry image (black box).

The evaluation of the intensity of the staining (negative, low, medium or high) was done by the *Human Protein Atlas* database and a graphical representation was done, showing the percentage of different intensity levels for each staining.

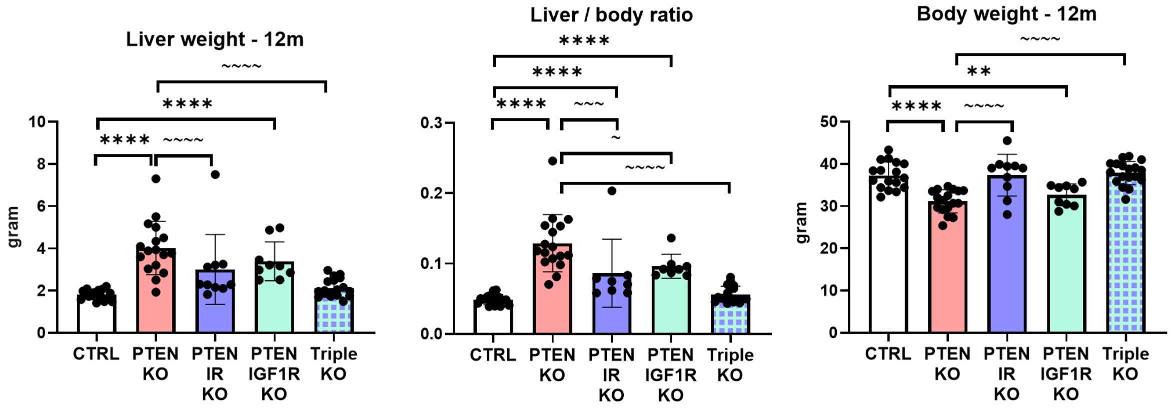

Fig. S11: Liver and body weight at 12 months of age.

Ex vivo assessment of liver weight (left panel), liver weight presented as percentage of body weight (middle panel), and body weight (right panel) in 12 months old CTRL, PTENKO, PTEN-IRKO, PTEN-IGF1RKO and triple-KO mice. Outliers were removed following ROUT (Q=1%) test and one-way ANOVA was performed. Values were considered significant when compared to CTRL (*) and to PTENKO (~):
*/~ p≤ 0.05, **/~~ p≤ 0.01,***/~~~ p≤ 0.001 or ****/~~~~ p≤ 0.0001.

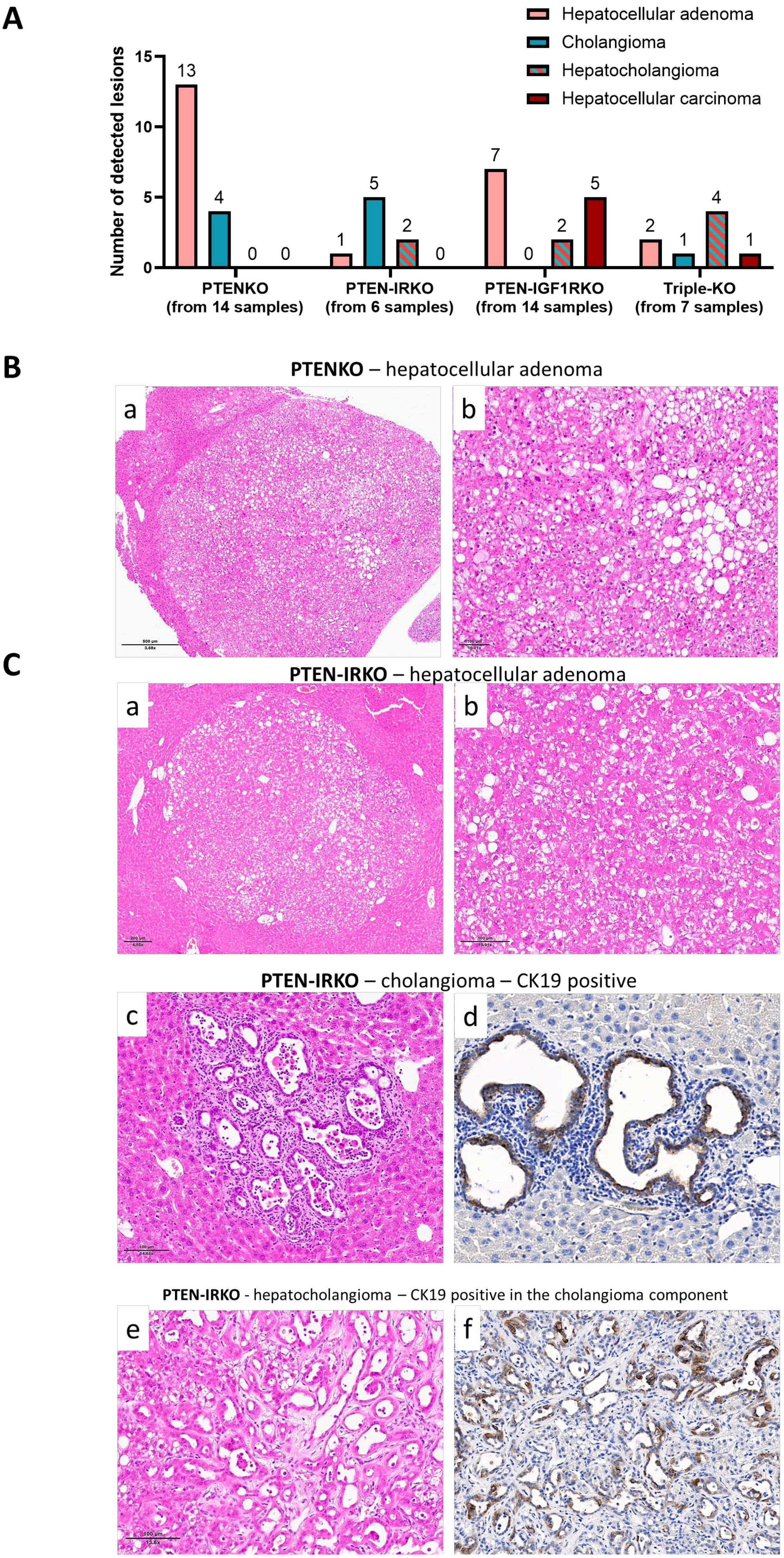
A
Hepatocellular adenoma
Cholangioma
Hepatocholangioma
Hepatocellular carcinoma
Number of detected lesions
0
5
10
15
13
4
0
0
1
5
2
0
7
0
2
5
2
1
4
1
PTENKO (from 14 samples)
PTEN-IRKO (from 6 samples)
PTEN-IGF1RKO (from 14 samples)
Triple-KO (from 7 samples)
B
PTENKO – hepatocellular adenoma
a
b
C
PTEN-IRKO – hepatocellular adenoma
a
b
PTEN-IRKO – cholangioma – CK19 positive
c
d
PTEN-IRKO - hepatocholangioma – CK19 positive in the cholangioma component
e
f

D **PTEN-IGF1RKO** – hepatocellular carcinoma

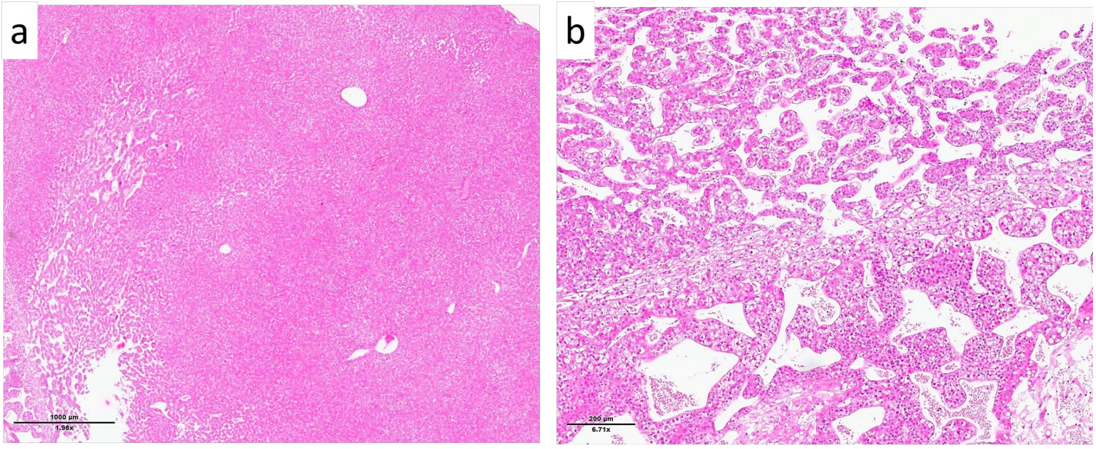

E **Triple-KO** – hepatocholangioma – CK19 positive in the cholangioma component

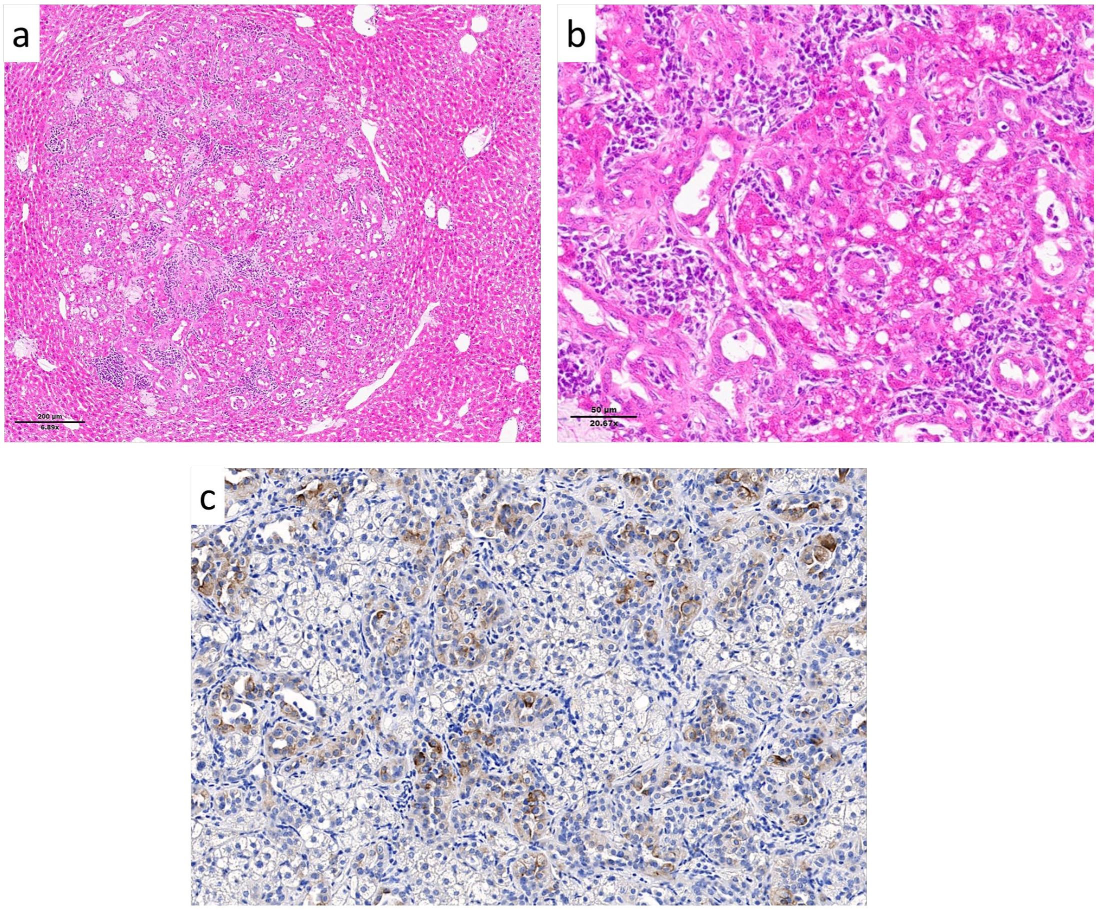

Fig. S12: Hepatic tumor incidence and histology.

H&E staining and CK19 immunohistochemistry of livers/hepatic tumors of 12 months-old PTENKO, PTEN-IRKO, PTEN-IGF1RKO and triple-KO mice.

(A) Incidence rate of different types of tumors in the mutants analyzed by histological assessment. Hepatic samples were randomly selected (PTENKO n=14, PTEN-IRKO n=6 PTEN-IGF1RKO n=14, triple-KO n=7). Representative images from each group are shown below (panels B-E).

(B) PTENKO mice: Representative H&E staining images of liver cell adenoma at (a) OMx3.68 and at (b) OMx10, negative for CK19: a nodule of well-differentiated hepatocytes with borders pushing towards the adjacent parenchyma. The liver cell plates are thicker than normal but no larger than two or three cells at the maximum. Hepatocytes have a large clear cytoplasm.

(C) PTEN-IRKO mice: Representative H&E staining images of liver cell adenoma at (a) OMx4.55 and at (b) OMx16, negative for CK19; (c) cholangioma at OMx14.6, (d) positive for CK19; and (e) hepatocholangioma at OMx13.6, (f) positive for CK19 staining in the cholangioma department. The mixed hepatobiliary tumors are a mixture of irregular ductular bile structures lined either by cuboïdal cells or by thin small cells and of liver cell plates or nests with well-differentiated hepatocytes showing either an eosinophilic cytoplasm or some steatosis.

(D) PTEN-IGF1RKO mice: a) Hepatocellular carcinoma at (a) OMx1.98 and (b) OMx6.71, negative for CK19. Large nodule composed of malignant hepatocytes arranged in thick trabeculae along large vascular channels. Hepatocytes have a large eosinophilic cytoplasm and there is some nuclear polymorphism. Mitoses are found. There are areas of necrosis and the lesion is not well delimitated.

(E) Triple-KO mice. Hepatocholangioma at (a) OMx6.89 and (b) OMx20.87, (c) CK19 positive in the cholangioma component.

Regarding the CK19 immunohistochemistry staining: Antigen retrieval was done with citrate buffer pH 6, followed by 15 min treatment H_2O_2 3%. The saturation of the slides was done with 10% NGS. CK19 primary antibody was incubated overnight at 4°C. The next day, secondary HRP antibody was used and staining was revealed with the Abcam DAB kit.

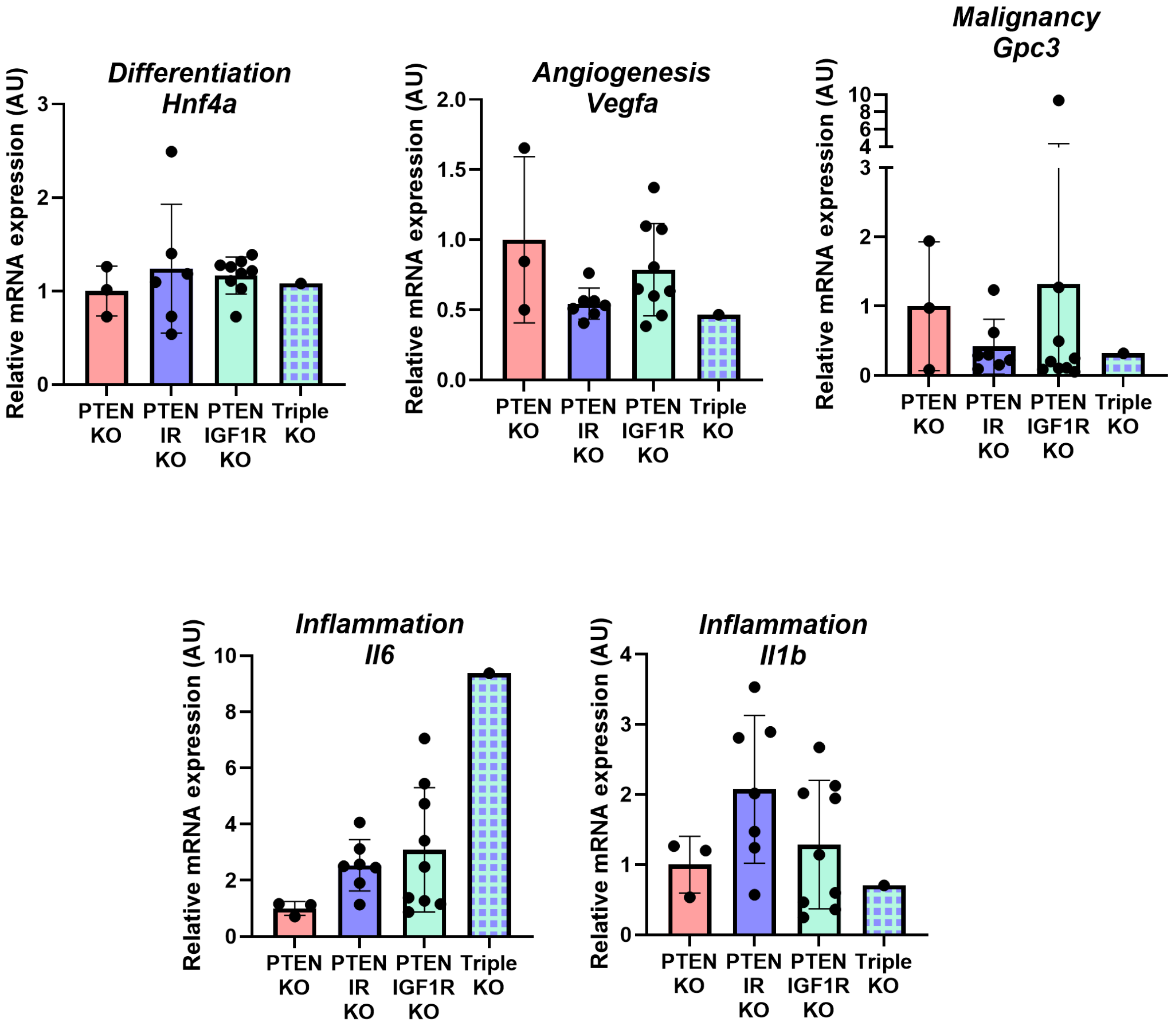

Fig. S13: Cancer marker expression in dissected tumors.

mRNA expression of *Hnf4, Vegfa, Gpc3, Il6 and Il1b* was investigated under fed conditions in dissected tumor samples of PTENKO (n=3), PTEN-IRKO (n=7), PTEN-IGF1RKO (n=9) and triple-KO (n=1) mice at 12 months of age *via* RT-qPCR. Outliers were removed following ROUT (Q=1%) test and one-way ANOVA was performed. Values are represented as mean +/- SD.

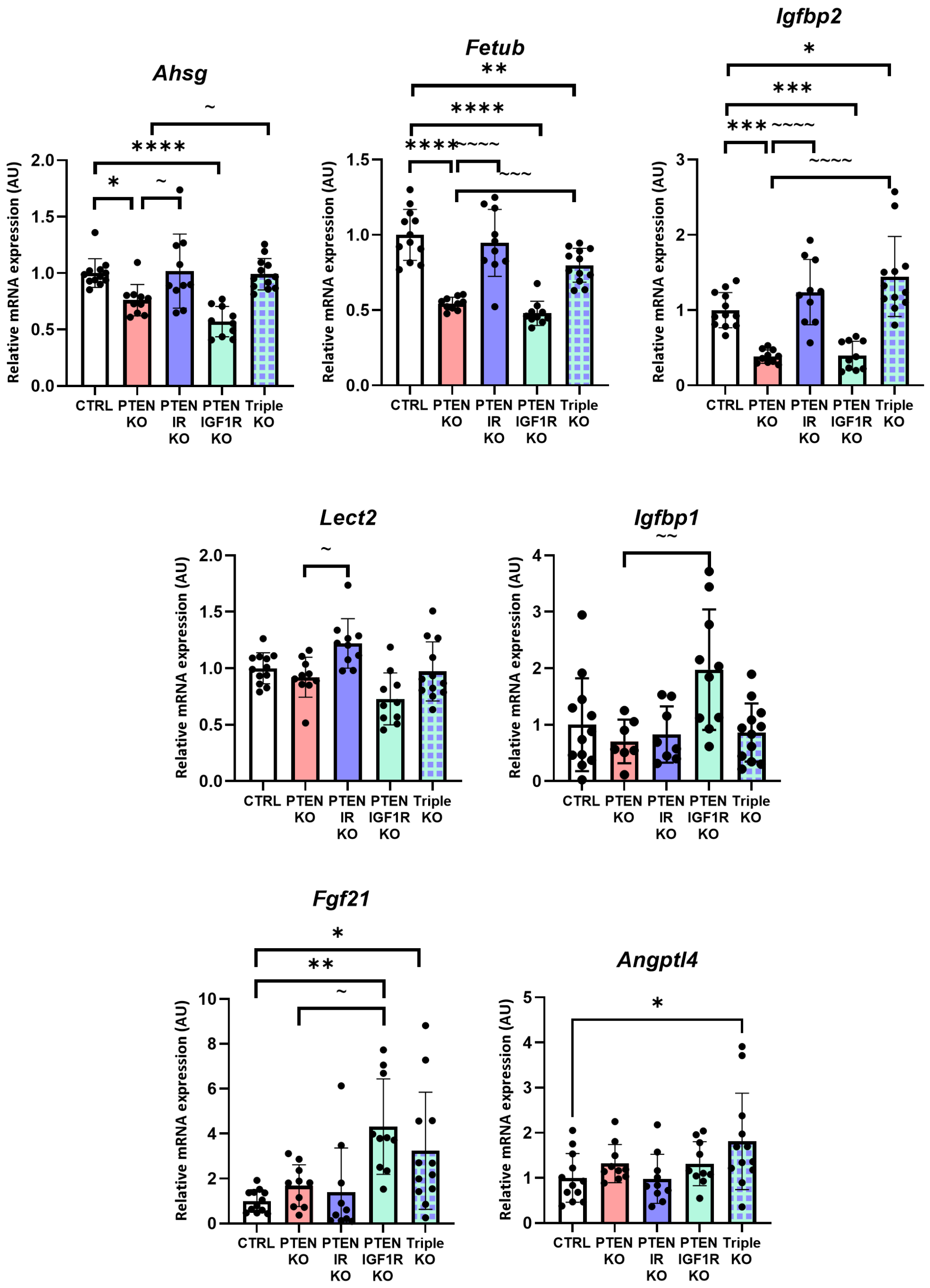

Fig. S14: Hepatokine expression in the liver.

Hepatokine mRNA expression was investigated under fed conditions in the livers of CTRL, PTENKO, PTEN-IRKO, PTEN-IGF1RKO and triple-KO mice at 4 months of age *via* RT-qPCR of *Ahsg* (Fetuin-A), *Fetub* (Fetuin-B), *Angptl4* (Angiopoietin-Like 4), *Igfbp1* (Insulin-like Growth Factor Binding Protein 1), *Igfbp2* (Insulin-like Growth Factor Binding Protein 2), *Lect2* (Leukocyte Cell Derived Chemotaxin 2) and *Fgf21* (Fibroblast Growth Factor 21). Values are mean +/- SD of 8-12 mice per group. Outliers were removed following ROUT (Q=1%) test and two-way ANOVA was performed, followed by a multiple comparison test. Values were considered significant when compared to CTRL (*) and to PTENKO (~): */~ p≤ 0.05, **/~~ p≤ 0.01,***/~~~ p≤ 0.001 or ****/~~~~ p≤ 0.0001.

A

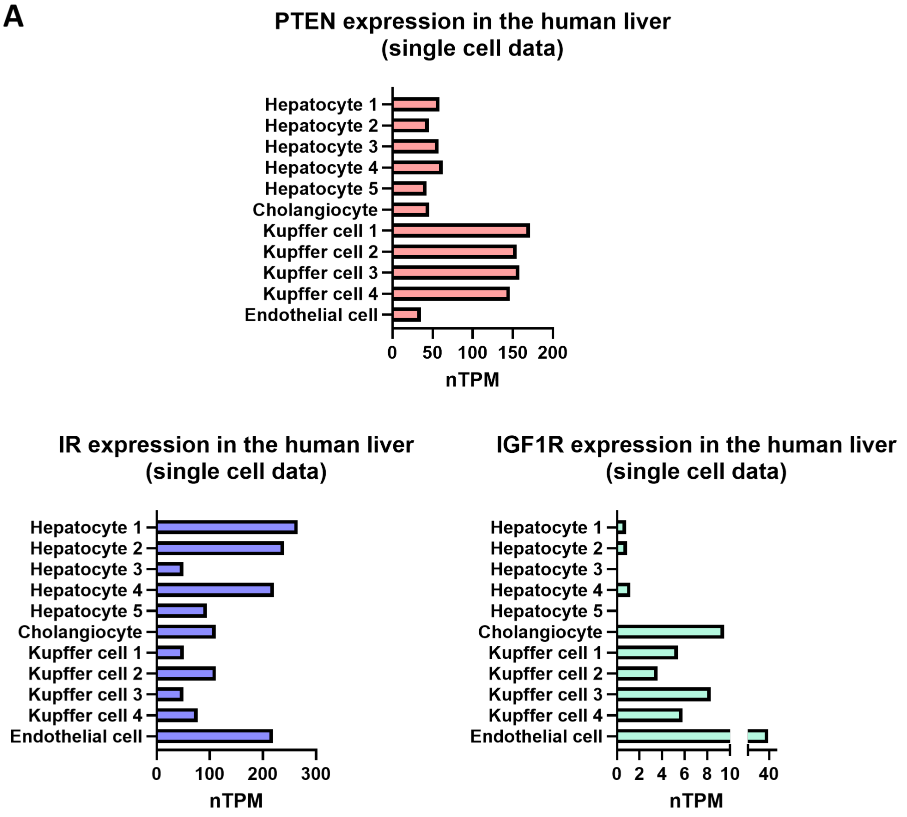

B

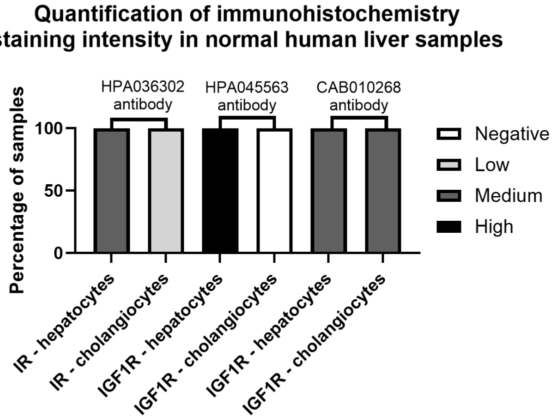

Fig. S15: RNA and protein levels of IGF1R and IR in hepatocytes and cholangiocytes in normal human liver.

(A) Single-cell RNA gene expression data for PTEN, IGF1R and INSR in human liver were obtained through the Human protein atlas database (proteinatlas.org). RNA expression is expressed in normalized transcript per million (nTPM).

(B) Protein levels of IR and IGF1R in human hepatocytes and cholangiocytes were assessed through immunohistochemistry of these proteins in healthy human liver samples, available in the publicly available human protein atlas database (proteinatlas.org). The evaluation of the intensity of the staining (negative, low, medium or high) was done by the Human Protein Atlas database and a graphical representation was done, showing the percentage of different intensity levels for each staining. For IGF1R, two different antibodies were used for immunohistochemistry (HPA045563 and CAB010268, scored separately).

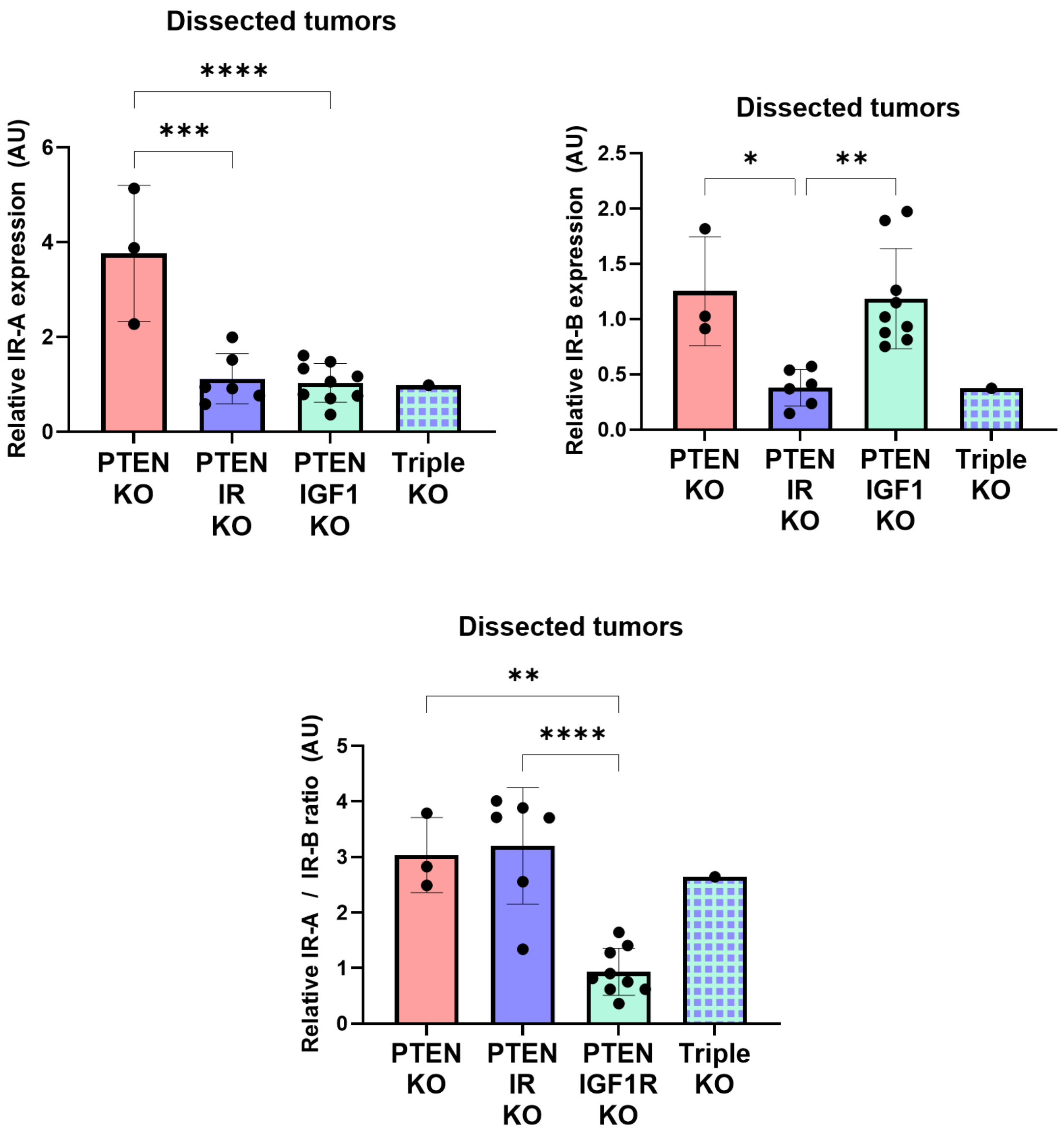

Fig. S16: IR-A and IR-B expression in the dissected tumors.

mRNA expression of IR-A and IR-B isoform and IR-A/IR-B ratio was investigated under fed conditions in dissected tumor samples of PTENKO, PTEN-IRKO, PTEN-IGF1RKO and triple-KO mice at 12 months of age *via* RT-qPCR. Outliers were removed following ROUT (Q=1%) test and one-way ANOVA was performed. Values are mean +/- SD of 1-9 mice per group. Values were considered significant when * p≤ 0.05, ** p≤ 0.01, *** p≤ 0.001 or **** p≤ 0.0001.

.

	PMID	Publication	Role
IGF1R in hepatocellular carcinoma	28619711	IGFBP7 Deletion Promotes Hepatocellular Carcinoma	Pro-oncogenic
	28202495	Expression of GRK2 and IGF1R in Hepatocellular Carcinoma: Clinicopathological and Prognostic Significance	Pro-oncogenic
	31732382	miR-455-5p Suppresses Hepatocellular Carcinoma Cell Growth and Invasion via IGF-1R/AKT/GLUT1 Pathway by Targeting IGF-1R	Pro-oncogenic
	30360707	Insulin-like Growth Factor I Receptor: A Novel Target for Hepatocellular Carcinoma Gene Therapy	Pro-oncogenic
	28624790	Co-targeting of IGF1R/mTOR Pathway by miR-497 and miR-99a Impairs Hepatocellular Carcinoma Development	Pro-oncogenic
	26658144	Tumour Initiating Cells and IGF/FGF Signalling Contribute to Sorafenib Resistance in Hepatocellular Carcinoma	Pro-oncogenic
	31267557	GSTZ1-1 Deficiency Activates NRF2/IGF1R Axis in HCC via Accumulation of Oncometabolite Succinylacetone	Pro-oncogenic
	29156819	miR-29a-3p Suppresses Cell Proliferation and Migration by Downregulating IGF1R in Hepatocellular Carcinoma	Pro-oncogenic
	31160483	MiR-505 Suppressed the Growth of Hepatocellular Carcinoma Cells via Targeting IGF-1R	Pro-oncogenic
	27126374	Abrogating the Interplay Between IGF2BP1, 2 and 3 and IGF1R by let-7i Arrests Hepatocellular Carcinoma Growth	Pro-oncogenic
	29881824	Inhibition of insulin-like growth factor 1 receptor enhances the efficacy of sorafenib in inhibiting hepatocellular carcinoma cell growth and survival	Pro-oncogenic
	22895605	Inhibition of hepatocellular carcinoma cell growth by an anti-insulin-like growth factor-I receptor monoclonal antibody	Pro-oncogenic
	31114322	microRNA-944 Inhibits the Malignancy of Hepatocellular Carcinoma by Directly Targeting IGF-1R and Deactivating the PI3K/Akt Signaling Pathway	Pro-oncogenic

	PMID	Publication	Role
IR in hepatocellular carcinoma	30849481	Insulin receptor isoform A favors tumor progression in human hepatocellular carcinoma by increasing stem/progenitor cell features	Pro-oncogenic
	28710407	Role of insulin receptor substrates in the progression of hepatocellular carcinoma	Pro-oncogenic
	23633480	Mitogenic Insulin Receptor-A Is Overexpressed in Human Hepatocellular Carcinoma due to EGFR-Mediated Dysregulation of RNA Splicing Factors	Pro-oncogenic
	21411721	Upregulation of the Insulin Receptor and Type I Insulin-Like Growth Factor Receptor Are Early Events in Hepatocarcinogenesis	Pro-oncogenic

Table S1: Available literature on IR and IGF1R in hepatic carcinogenesis.

Supplementary materials and methods:

EchoMRI and CT-scan

Lean mass was measured using quantitative nuclear magnetic resonance analyzer EchoMRI-700 (Echo Medical Systems, Houston, TX). The fat depot distribution was imaged by multidetector CT-scan (Discovery 750 HD, GE Healthcare, Milwaukee, USA) and the volume was quantified by OsiriX imaging software (*Version 3.9*). The weight of major adipose depots was also investigated by dissection at the end of the experimental protocols.

Livers of 12 months aged mice were imaged by *in vivo* X-Ray computed tomography (CT scan) (Skysan 1076, Bruker, Belgium). Injection of the contrast agent ExiTron^{TM} Nano12000 (MiltenyiBioTec, Germany) allowed the visualization of benign and malignant tumors. Liver volume, tumor number, and tumor volume were quantified by OsiriX imaging software (*Version 3.9*).

Glucose / pyruvate tolerance tests (GTT / PTT) and insulin injections

Mice were fasted overnight prior to intraperitoneal administration of glucose (1.5 g/kg) or pyruvate (2 g/kg). Blood glycemia was measured from tail vein during 2h.

Metabolic phenotyping

Indirect calorimetry, locomotor activity, food, and fluid intake were measured for 48 hours using Labmaster metabolic cages (TSE, Bad Homburg, Germany) after 3 days of adaptation prior to recording (mice were housed individually). Energy expenditure and respiratory exchange ratio were calculated as described [2].

Histological analyses

Tissue samples were fixed in 4% paraformaldehyde, embedded in paraffin, and cut into 5µm sections. For morphological investigations, hematoxylin/eosin staining was performed, and images were acquired using AxioScan.Z1 slide scanner (Zeiss, Germany).

Plasma and tissue analyses

Plasma glucose were determined by medical analysis laboratories accredited for the processing of patient samples (TEST Tailored Efficient Swiss Testing SA and AnaBio, Geneva, Switzerland). An automated Abott Architect analyzer (Abott Architect, Paris, France) was used to determine AST and ALT. Plasma insulin, glucagon as well as liver content of triglycerides, cholesteryl esters, and ketone bodies were measured with commercial kits (Supplementary Material and Methods). Hepatic glycogen content was assessed as previously described [3].

RNA extraction and real-time PCR

Trizol Reagent® was used to extract RNA and 1µg of RNA was reverse-transcribed using High-Capacity RNA-to-cDNA Kit (AppliedBiosystems). The quantitative real time PCR analysis was performed with the SYBR® Select Master Mix and the StepOne Plus® PCR system (Life Technologies). Primer sequences are listed in Supplementary Material and Methods. The following settings were used for the PCR amplifications: 40 cycles with: 94°C for 2 min, 60°C for 1 min, 68°C for 2 min. Results were normalized to the mRNA expression of cyclophilin A, β-actin and RPS9 using the geNorm algorithm.

Western blot analysis

Tissues were lysed in ice-cold RIPA buffer supplemented with a cocktail of protease inhibitors (Roche, Rotkreuz, Switzerland) using Tissue Lyser II (Qiagen, Düsseldorf, Germany). Proteins

were resolved by 5-20% gradient SDS-PAGE and blotted onto nitrocellulose membranes (Amersham, GE Healthcare, Buckinghamshire, UK). Detection of proteins was done with specific primary antibodies and HRP-conjugated secondary antibodies using chemoluminescence (Supplementary Material and Methods). The PXi system from SYNGENE (SYNOPTICS group, Cambridge, UK) and the GeneTools Software 4.03.00 were used to detect and quantify the signals.

***In silico* analysis**

Expression of INSR and IGF1R in hepatic cancer in patients

In silico assessment of the expression of INSR and IGF1R was performed in hepatocellular carcinoma samples *vs.* normal hepatic tissue in the cancer genome atlas (TCGA) cohort of patients (LIHC – liver hepatocellular carcinoma subgroup) [4]. Analysis and graphical representation were done by the Gepia2 cancer database (http://gepia2.cancer-pku.cn/#index, accessed 18.07.2023).

Survival of hepatic cancer patients

Gepia2 was used to assess the survival of LIHC patients from the TCGA cohort. Patients from the cohort were segregated into 2 groups – group 1 containing 10% of the patients which exhibit the highest expression of either INSR or IGF1R versus group 2 containing the remaining 90% of the patients with lower expression. Kaplan-Meier representation and statistical analysis were done by the Gepia2 database.

Expression of INSR and IGF1R in HCC at different stages

The Human Protein atlas (https://www.proteinatlas.org/ accessed 20.11.2023) was used to assess the expression levels of *INSR* and *IGF1R* at different stages of HCC in LIHC patients from the TCGA cohort. Patients from the cohort were segregated into 3 groups – stage I (n=102), stage II (n=84) and stage III (n=83). Patients with stage IV HCC were not considered as the number of individuals was very low (n=4). Patients with no staging information were also not considered. The human protein atlas was also used to assess the gene expression data for PTEN, IGF1R and INSR in human liver, on a single-cell level (single cell RNA seq database). RNA expression is expressed in normalized transcript per million (nTPM).

GEOdataset analysis of expression of INSR and IGF1R in HCC vs normal liver

The Gene expression omnibus (GEO) datasets (ncbi.nlm.nih.gov/gds) were accessed 17.11.2023. The expression levels of *INSR* and *IGF1R* were determined in 4 different GEOdatasets of HCC patients (GSE14520, n=19, GSE25097, n=243, GSE60502, n=18, GSE64041, n=60) and the fold change in expression between HCC and non-tumoral biopsy was determined for each patient. Expression data are presented as log10 transformed.

GEOdataset	*Type of dataset*	*INSR ID*	*IGF1R ID*	*PMID*
GSE14520	Array	207851_s_at	203627_at	[5]
GSE25097	Array	100148812_TGI_at	100148401_TGI_at	[6]
GSE60502	Array	207851_s_at	203627_at	[7]
GSE64041	Array	8033362	7986359	[8]

GSEA analysis

The GSEA software was used to assess the GSE36376 dataset containing expression analysis from human HCC (240 human HCC samples, segregated between the 20% most expressing

IGF1R or *INSR* and the rest 80% of the samples). Enrichment was calculated based on 1000 permutations (type of permutation – phenotype), using the number of gene ranged at the bottom or the top of the list of genes associated. The sets of genes presenting a FDR q-value < 0.05, were taken as significant.

Statistics

Statistical significance of the results was determined by ANOVA test followed by a Holm-Sidak post-hoc test. Outlier test was performed (ROUT 1%) and outliers were removed. Values were considered significant when compared to CTRL (*) and to PTENKO (~) */~ p≤ 0.05, **/~~ p≤ 0.01, ***/~~~ p≤ 0.001 or ****/~~~~ p≤ 0.0001. Statistical differences between fed and fasted state were represented as follows: ^ p≤ 0.05, ^^ p≤ 0.01, ^^^ p≤ 0.001 or ^^^^ p≤ 0.0001.

Supplementary references

1. Horie, Y., A. Suzuki, E. Kataoka, et al., *Hepatocyte-specific Pten deficiency results in steatohepatitis and hepatocellular carcinomas.* J Clin Invest, 2004. **113**(12): p. 1774-83.
2. Peyrou, M., L. Bourgoin, A.L. Poher, et al., *Hepatic PTEN deficiency improves muscle insulin sensitivity and decreases adiposity in mice.* J Hepatol, 2015. **62**(2): p. 421-9.
3. Monteillet, L., M. Gjorgjieva, M. Silva, et al., *Intracellular lipids are an independent cause of liver injury and chronic kidney disease in non alcoholic fatty liver disease-like context.* Mol Metab, 2018. **16**: p. 100-115.
4. Tomczak, K., P. Czerwińska, and M. Wiznerowicz, *The Cancer Genome Atlas (TCGA): an immeasurable source of knowledge.* Contemp Oncol (Pozn), 2015. **19**(1a): p. A68-77.
5. Roessler, S., H.L. Jia, A. Budhu, et al., *A unique metastasis gene signature enables prediction of tumor relapse in early-stage hepatocellular carcinoma patients.* Cancer Res, 2010. **70**(24): p. 10202-12.
6. Tung, E.K., C.K. Mak, S. Fatima, et al., *Clinicopathological and prognostic significance of serum and tissue Dickkopf-1 levels in human hepatocellular carcinoma.* Liver Int, 2011. **31**(10): p. 1494-504.
7. Wang, Y.H., T.Y. Cheng, T.Y. Chen, et al., *Plasmalemmal Vesicle Associated Protein (PLVAP) as a therapeutic target for treatment of hepatocellular carcinoma.* BMC Cancer, 2014. **14**: p. 815.
8. Makowska, Z., T. Boldanova, D. Adametz, et al., *Gene expression analysis of biopsy samples reveals critical limitations of transcriptome-based molecular classifications of hepatocellular carcinoma.* J Pathol Clin Res, 2016. **2**(2): p. 80-92.
