# Supplementary material for: Hepatic IR and IGF1R signaling govern distinct metabolic and carcinogenic processes upon PTEN deficiency in the liver
Source: JHEP Rep. 2024 Dec 19;7(4):101305. doi: 10.1016/j.jhepr.2024.101305 (PMC11925173; doi:10.1016/j.jhepr.2024.101305)
Supplement: Multimedia component 2 [file mmc2.docx]

**JHEP Reports**

**CTAT methods**

Tables for a “Complete, Transparent, Accurate and Timely account” (CTAT) are now mandatory for all revised submissions. The aim is to enhance the reproducibility of methods.

- Only include the parts relevant to your study
- Refer to the CTAT in the main text as ‘Supplementary CTAT Table’
- Do not add subheadings
- Add as many rows as needed to include all information
- Only include one item per row

**If the CTAT form is not relevant to your study, please outline the reasons why:**

|  |
| --- |

- 1. **Antibodies**

| **Name** | **Citation** | **Supplier** | **Cat no.** | **Clone no.** |
| --- | --- | --- | --- | --- |
| FAS | Chrysanthi Moschandrea, et. al. Nature, 2023 | Cell Signaling (MA, USA) | 3189 | NA |
| ACC | Lulu Chen, et. al. Nat Chem Biol, 2024 | Cell Signaling (MA, USA) | 3662 | NA |
| SCD1 | Liangkui Li, et. al., J Lipid Res, 2023 | Cell Signaling (MA, USA) | 2438 | NA |
| CPT1 | Marion Adam et al.  FASEB, 2018 | Merck Millipore | ABS65 | NA |
| ERM | Elisabeth Ott, et. al., Sci Rep, 2023 | Cell Signaling (MA, USA) | 3142 | NA |
| UCP1 | Williams et al., J Obes, 2023 | ThermoFisher | PA1-24894 | NA |
| CK19 | Sunami et al., Cells, 2022 | ThermoFisher | MA5-31977 | NA |
| Alexa Fluor 488 |  | Molecular probes | A-11008 | NA |
| HRP-conjugated anti-rabbit |  | Biorad (Cressier, Switzerland) | 170-6515 | NA |

- 1. **Cell lines**

| **Name** | **Citation** | **Supplier** | **Cat no.** | **Passage no.** | **Authentication test method** |
| --- | --- | --- | --- | --- | --- |
| **NA** | **NA** | **NA** | **NA** | **NA** | **NA** |

- 1. **Organisms**

| **Name** | **Citation** | **Supplier** | **Strain** | **Sex** | **Age** | **Overall n number** |
| --- | --- | --- | --- | --- | --- | --- |
| Mouse | NA | University of Geneva animal facility | C57BL/6 | male | 4 mths,  12 mths |  |

- 1. **Sequence based reagents**

| **Target gene** | **Forward primer (5' - 3')** | **Reverse primer (5' - 3')** | **Supplier** |
| --- | --- | --- | --- |
| *Acat1* | GGACATCAGGATGTGATGG | CATATGGTGTTGCTCCTCTG | Microsynth, Switzerland |
| *Acat2* | GCTATAGGCTCCTTCAATGGT | GTCGAGTAGGATTCTGCCC | Microsynth, Switzerland |
| *Acc1* | GGACACCAGTTTTGCATTGA | AGTTTGGGAGGACATCGAAA | Microsynth, Switzerland |
| *Abcg5* | GCTCTTCCAACACTTCGAC | CAGAACACCAACTCTCCG | Microsynth, Switzerland |
| *Acox1* | CATGAATCCCGATCTGCG | TCAAGTTCTCGATTTCTCGAC | Microsynth, Switzerland |
| *Adipoq* | TCCTGGAGAGAAGGGAGAGAAAG | TCATTCCAACATCTCCTGTCTCA | Microsynth, Switzerland |
| *Cd36* | GTCTATCTACGCTGTGTTCG | ACAGGCTTTCCTTCTTTGC | Microsynth, Switzerland |
| *Cyp7a1* | TTCTGCGAAGGCATTTGG | GGTCTCATGACAGATTGGAG | Microsynth, Switzerland |
| *Cpt1a* | ATGGCAGAGGCTCACCAAGC | GATGAACTTCTTCTTCCAGGAGTGC | Microsynth, Switzerland |
| *Dgat1* | GAGATTGGTGGAATGCTGAG | GCATAGGCTTGTAGAAGTGTC | Microsynth, Switzerland |
| *Fasn* | AAGTTGCCCGAGTCAGAGAACC | ATCCATAGAGCCCAGCCTTCCATC | Microsynth, Switzerland |
| *Fxr* | GCTGCAAAGGTTTCTTCC | GTACATGTCCATCACGCAG | Microsynth, Switzerland |
| *G6pc* | CTTCAAGTGGATTCTGTTTGG | CTTTATTATAGGCACGGAGCT | Microsynth, Switzerland |
| *Gck* | GCGGAGATGCTCTTTGAC | GTCCCACGATGTTGTTCC | Microsynth, Switzerland |
| *Gpat1* | AGGGATGATCAGGCTGAC | GAATCAAGGTACTGAAGACGG | Microsynth, Switzerland |
| *Hmgcoar* | GTACATTCTGGGTATTGCTGG | GCACTCGCTCTAGAAAGG | Microsynth, Switzerland |
| *Hmgcs1* | GGCAGAAAGAGGGAAAGGA | CAGGAACATCCGAGCTAGAG | Microsynth, Switzerland |
| *Hmgcs2* | CCTCTTCCCTCTATGGCT | AGAGAAGTTCACCTTGTGG | Microsynth, Switzerland |
| *Igf1r* | CTGATGTCTGGTCCTTCGG | TTCAAACAGCATATCAGGGCA | Microsynth, Switzerland |
| *Insr* | GCCAGTGATGTGTTTCCA | CCTCACCCTTGATGATATCC | Microsynth, Switzerland |
| *Lep* | CACACACGCAGTCGGTATCC | GTCCATCTTGGACAAACTCAGAATG | Microsynth, Switzerland |
| *Ldha* | CTCCAGCAAAGACTACTGTG | CCACTGGATTGGAGACGA | Microsynth, Switzerland |
| *Lxrb* | ATTAAGGAAGAGGGGCAGGA | TGACCACGATGTAGGCAGAG | Microsynth, Switzerland |
| *Mttp* | GAAAGAAGTGCTCCCTCAG | CCTTTGAACTTACTAAGGAGGG | Microsynth, Switzerland |
| *Pck1* | AGCTGCATAATGGTCTGG | CCGTCTTGCTTTCGATCC | Microsynth, Switzerland |
| *Pdha* | GTGACCCTGGAGTAAGCT | CACTTCCACATCAATCTCCT | Microsynth, Switzerland |
| *Ppargc1a* | GAAAACAGGAACAGCAGCAGAGAC | GGGGTCAGAGGAAGAGATAAAGTTG | Microsynth, Switzerland |
| *Pklr* | CTTTGCCTCCTTTGTACGA | TCATCAAACTTCTTCACGCC | Microsynth, Switzerland |
| *Ppara* | ACGATGCTGTCCTCCTTGATG | GTGTGATAAAGCCATTGCCGT | Microsynth, Switzerland |
| *Pparg* | CTCACAATGCCATCAGGT | GCTGGTCGATATCACTGG | Microsynth, Switzerland |
| *Pten* | ACACCGCCAAATTTAACTGC | TACACCAGTCCGTCCCTTTC | Microsynth, Switzerland |
| *Scd1* | TGCTCCAAGAGATCTCCAG | GGAACCAGTATGATCCCG | Microsynth, Switzerland |
| *Srebp1c* | AAGCAAATCACTGAAGGACCTGG | AAAGACAAGGGGCTACTCTGGGAG | Microsynth, Switzerland |
| *Srebp2* | CCGCTCTCGAATCCTCTTAT | CAGCACCTGACTCCAGTGAC | Microsynth, Switzerland |
| *Ppia* | CAAATGCTGGACCAAACACAA | GCCATCCAGCCATTCAGTCT | Microsynth, Switzerland |
| *Rps9* | GACCAGGAGCTAAAGTTGATTGGA | TCTTGGCCAGGGTAAACTTGA | Microsynth, Switzerland |
| *Actb* | CTAAGGCCAACCGTGAAAAGAT | CACAGCCTGGATGGCTACGT | Microsynth, Switzerland |
| *Ucp1* | CGTACCAAGCTGTGCGATGT | AAGCCACAAACCCTTTGAAAAA | Microsynth, Switzerland |
| *Igfbp1* | CTGGACAGCTTCCACCTGAT | GTTGGGCTGCAGCTAATCTC | Microsynth, Switzerland |
| *Igfbp2* | CTTAAGCAGTGCAAGATGTCTC | GTTTACTGCACACTTTGGGC | Microsynth, Switzerland |
| *Fgf21* | CAGTCCAGAAAGTCTCCTG | GATCAAAGTGAGGCGATCC | Microsynth, Switzerland |
| *Lect2* | TAGCAGGACCATGGGCTAAC | GCCCACTATCTTCCCAGTGA | Microsynth, Switzerland |
| *Fetub* | GGCCCTGCTTACTATGTGGA | TCTACGGGTTGGATCAGAGG | Microsynth, Switzerland |
| *Ahsg* | CCTCTCCCAGTGTCTACTC | AGCCATGTTGCTTCTCTG | Microsynth, Switzerland |
| *Angptl4* | CATCCTGGGACGAGATGAACT | TGACAAGCGTTACCACAGGC | Microsynth, Switzerland |
| *Ir-a* | TTTTTGTCCCCAGGCCAT | CCTGTGCTCCTCCTGACTTG | Microsynth, Switzerland |
| *Ir-b* | AACCTCTTCAGGCAATGGTG | AGGAGACGTTGGGGAAATCT | Microsynth, Switzerland |
| *Hnf4a* | CTGCTCCTAGGCAATGAC | CTCACTTGCACCTGTGAC | Microsynth, Switzerland |
| *Vegfa* | GATCATGCGGATCAAACCT | CTTTCTTTGGTCTGCATTCAC | Microsynth, Switzerland |
| *Gpc3* | CAGCCCGGACTCAAATGGG | GCCGTGCTGTTAGTTGGTATTTT | Microsynth, Switzerland |
| *Il6* | GAGGATACCACTCCCAACAGACC | AAGTGCATCGTTGTTCATACA | Microsynth, Switzerland |
| *Il1b* | GACAACTGCACTACAGGC | CATGGAGAATATCACTTGTTGG | Microsynth, Switzerland |

***Mouse genotyping***

Genotyping was performed by PCR using genomic DNA isolated from fingertips of 6-10 days old mice with Kapa genotyping mastermix (KAPA Biosystems, Wilmington, MA, US). The primers for the identification of the Cre recombinase transgene (5`-CCTGCCAGCCATGGATATAA-3` and 5`-GTTGTCCTTTGTGCTGCTGA-3`) and the floxed alleles for *Pten* (5`-ACTCAAGGCAGGGATGAGC-3`; 5`-AATCTAGGGCCTCTTGTGCC-3` and 5`-GCTTGATATCGAATTCCTGCAGC-3`), *Insr* (5`-CTGAATAGCTGAGACCACAG-3`; 5`-CACACACACACGCCTACAC-3`and 5`-TCTCCCTACACCCACTCACA-3`) and *Igf1r* (5`-TGAGACGCGAGATTGCTGTA-3`; 5`-CTTCCCAGCTTGCTACTCTAGG-3` and 5`-CAGGCTTGCAATGAGACATGGG-3`). The primers were used under the following conditions: 1 cycle of 95°C for 3 min, 35 cycles of 95°C for 15 sec, 57°C for 15 sec, and 72°C for 20 sec, followed by one cycle of 72°C for 10 min.

- 1. **Biological samples**

| **Description** | **Source** | **Identifier** |
| --- | --- | --- |
|  |  |  |

- 1. **Deposited data**

| **Name of repository** | **Identifier** | **Link** |
| --- | --- | --- |
| **Yareta** | **Hepatic IR and IGF1R signaling govern distinct metabolic and carcinogenic processes upon PTEN deficiency in the liver.** | 10.26037/yareta:zwyyaxdj3zc3hfo3hykxvwmt6q |

- 1. **Software**

| **Software name** | **Manufacturer** | **Version** |
| --- | --- | --- |
|  |  |  |

- 1. **Other (*e.g*. drugs, proteins, vectors etc.)**

| **Name** | **Provider** | **Catalogue number** |
| --- | --- | --- |
| Nitrocellulose membranes | Amersham (Dübendorf, Switzerland) | RPN303D |
| ECL advance | Amersham (Dübendorf, Switzerland) | RPN2135 |
| Sodium pyruvate powder | Sigma (St Louis, MO, USA) | P5280-25G |
| Sodium chloride | Acros organics (Geel, Belgium) | 20779-0010 |
| Triton™X-100 | Sigma (St. Louis, MO, USA) | X-100 |
| Glucotrend® Active | Roche (Basel, Switzerland) | 04454308 |
| Insulin (Humalog®) | Eli Lilly (Vernier, Switzerland) | - |
| Complete EDTA-free inhibitors | Roche (Basel, Switzerland) | 11873580001 |
| Glucose 20% | Sintetica-Bioren (Couvet, Switzerland) | - |
| High Capacity RNA-to-cDNA Kit | AppliedBiosystems, Thermo Fisher Scientific(Waltham, MA, USA) | 4387406 |
| Isofluorane | Rothacher & Partner (Berne, Switzerland) | ISO250 |
| SYBR® Select Master Mix | Life Technologies, Thermo Fisher Scientific (Waltham, MA, USA) | 4472920 |
| Trizol Reagent | Invitrogen (Carlsbad, CA) | 15596-018 |
| Triglyceride kit | Roche/Hitachi (Rotkreuz, Switzerland) | 12016648-122 |
| Cholesterol/ Cholesteryl ester kit | Merck Calbiochem® (Billerica, MA, USA) | 428901-1 |
| β-Hydroxybutyrate (Ketone Body) Kit | Cayman Chemical (Ann Arbor, ML, USA) | 700190 |
| Insulin UltraSensitive ELISA kit | Mercodia (Uppsala, Sweden) | 10-1247-01 |
| Glucagon ELISA- 10ul | Mercodia (Uppsala, Sweden) | 10-1281-01 |

- 1. **Please provide the details of the corresponding methods author for the manuscript:**

| **Gjorgjieva Monika**  **Monika.gjorgjieva@unige.ch** |
| --- |

**2.0 Please confirm for randomised controlled trials all versions of the clinical protocol are included in the submission. These will be published online as supplementary information.**

| **NA** |
| --- |
