# Supplementary material for: Hepatic IR and IGF1R signaling govern distinct metabolic and carcinogenic processes upon PTEN deficiency in the liver
Source: JHEP Rep. 2024 Dec 19;7(4):101305. doi: 10.1016/j.jhepr.2024.101305 (PMC11925173; doi:10.1016/j.jhepr.2024.101305)
Supplement: Multimedia component 4 [file mmc4.pdf]

Blots under fed conditions

FAS fed

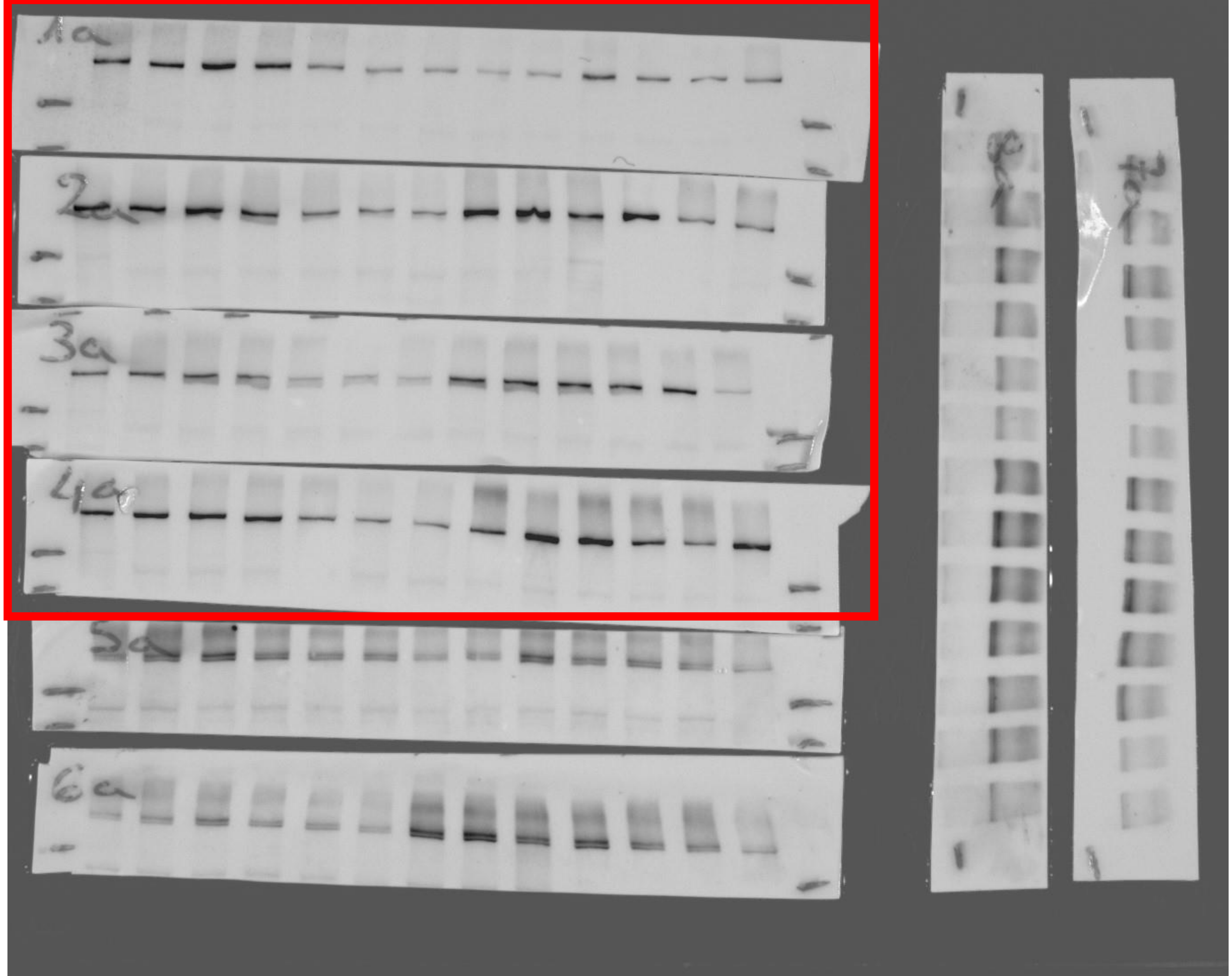

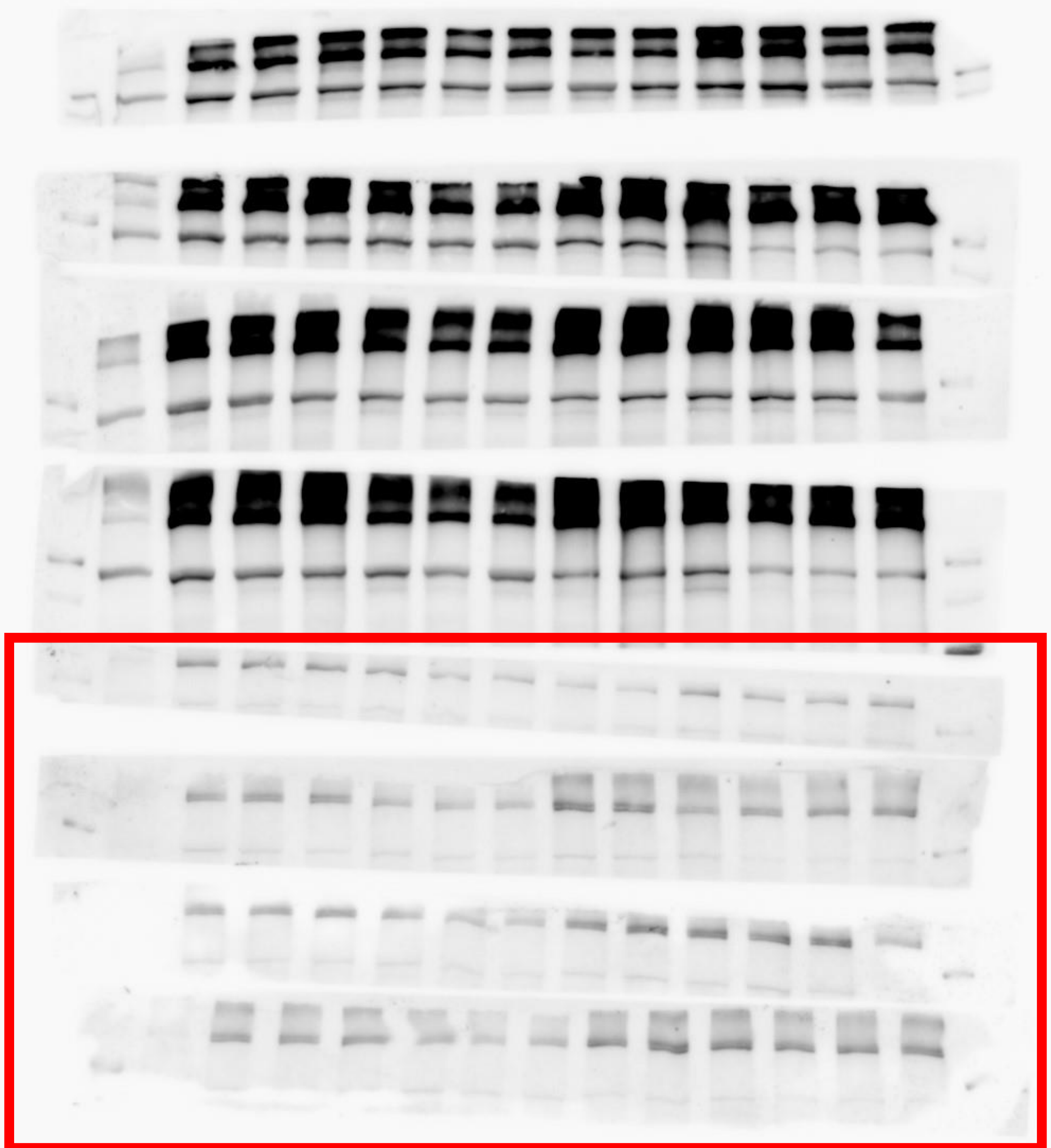

ACC fed

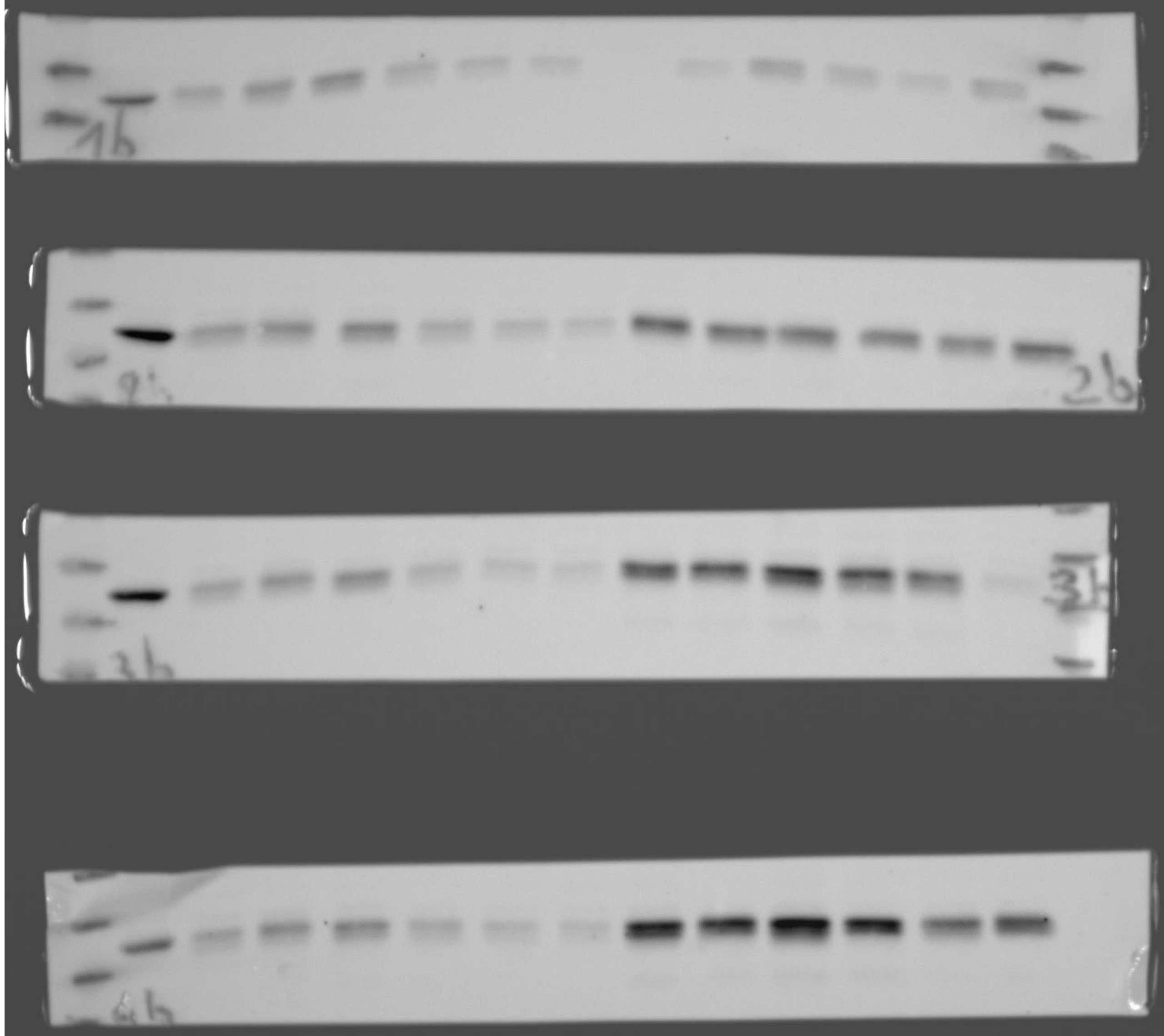
1b
2b
3h
SCD1 fed

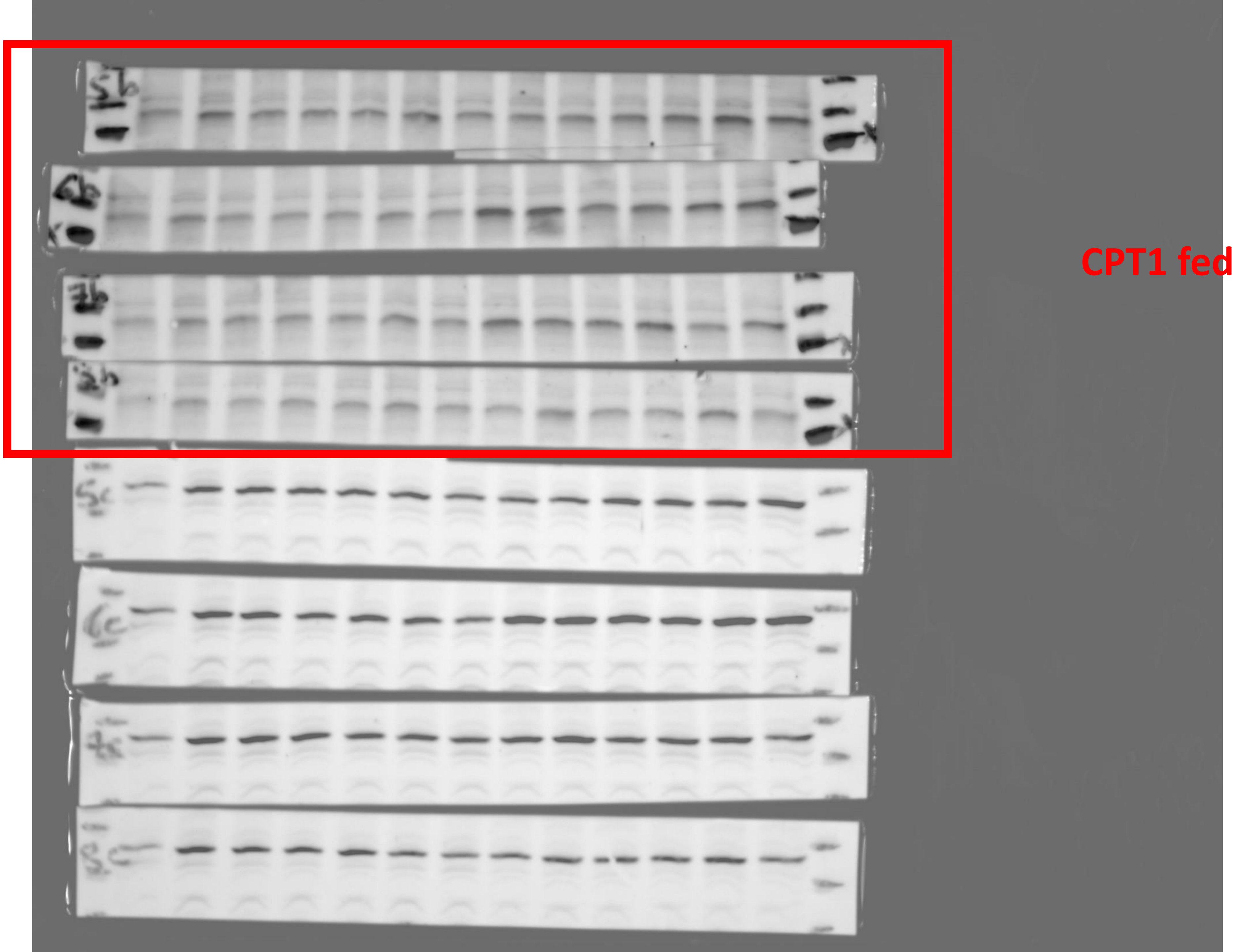
CPT1 fed

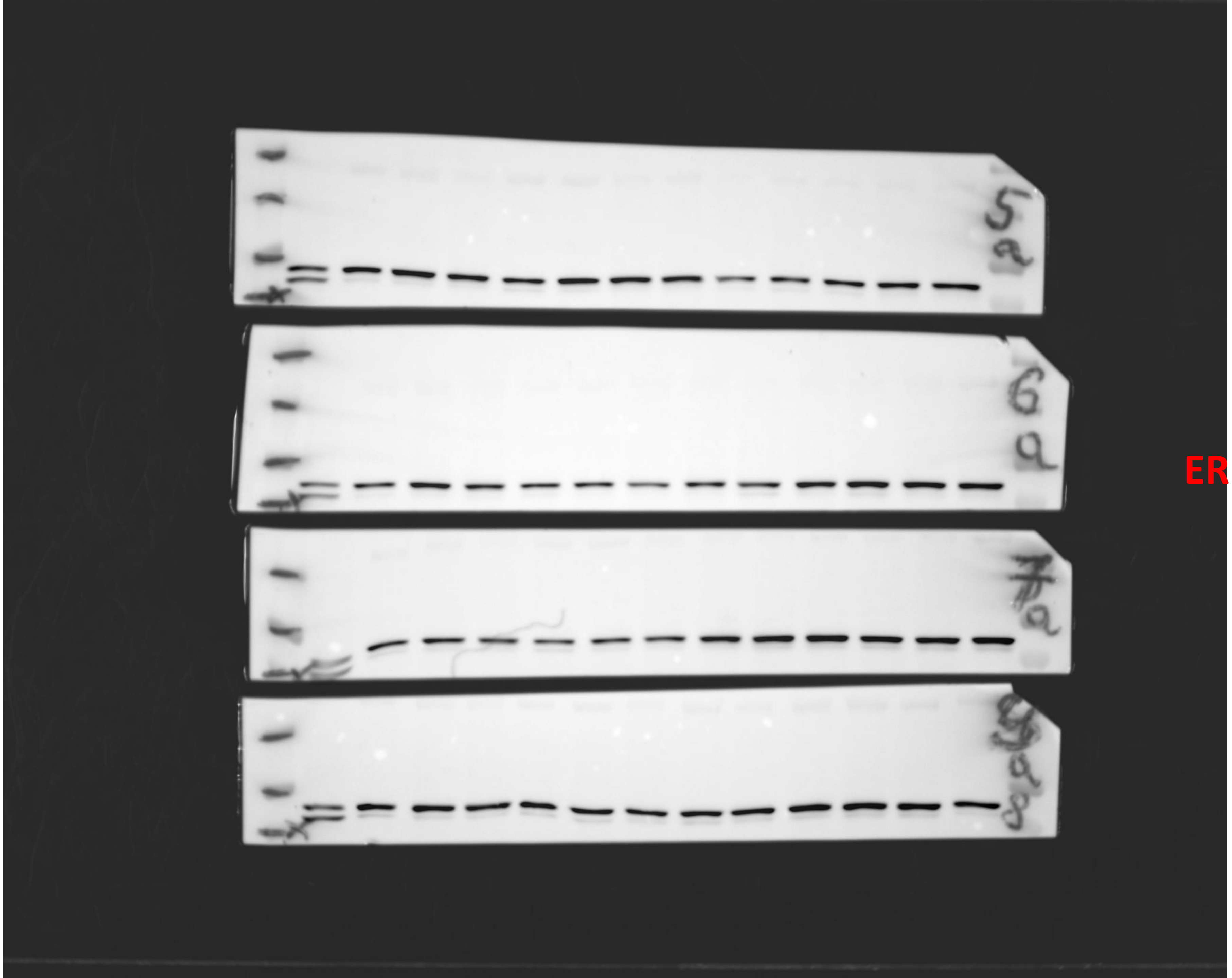

ERM fed

Blots under fasted conditions

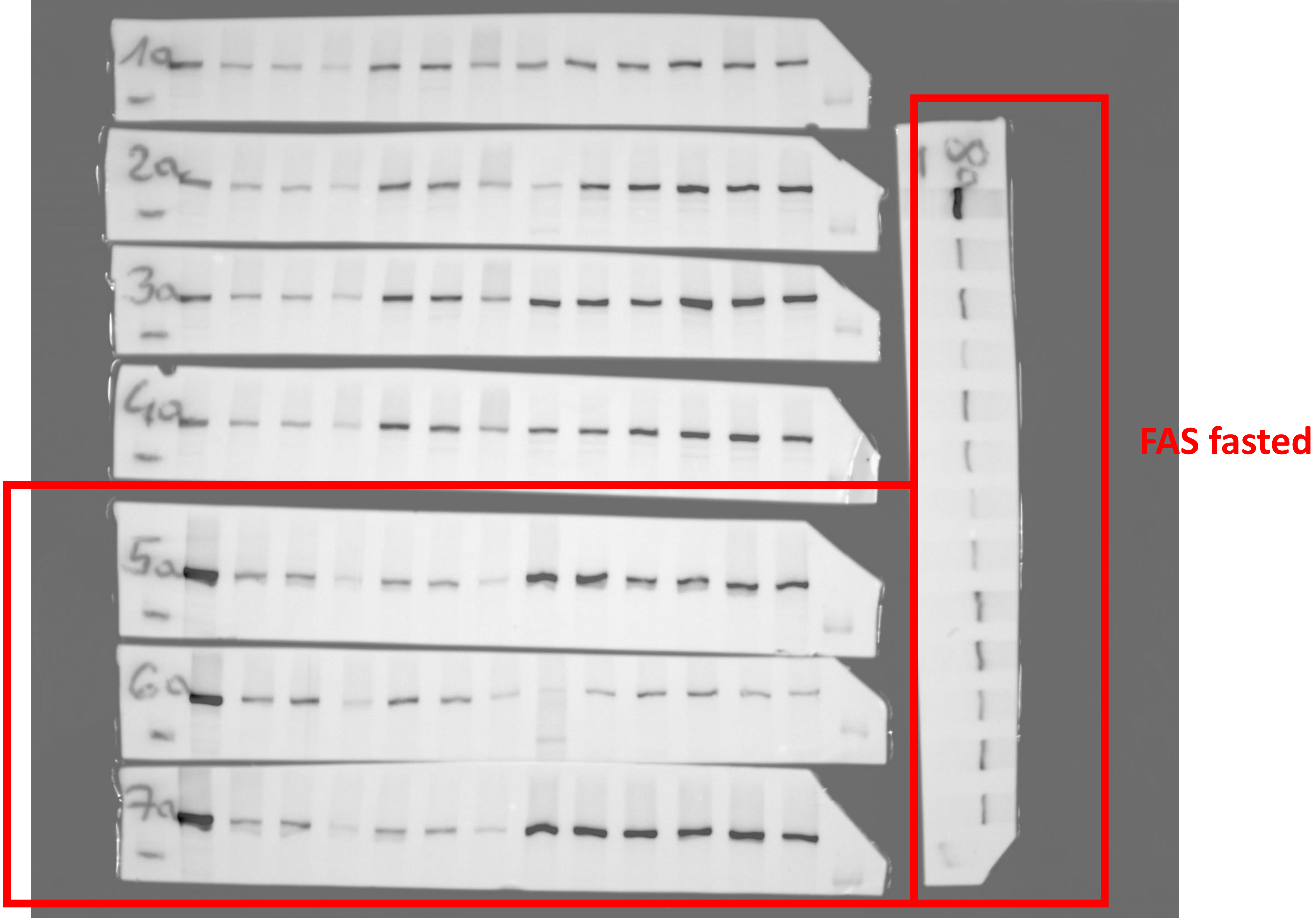

1a
2a
3a
4a
5a
6a
7a
8a
FAS fasted

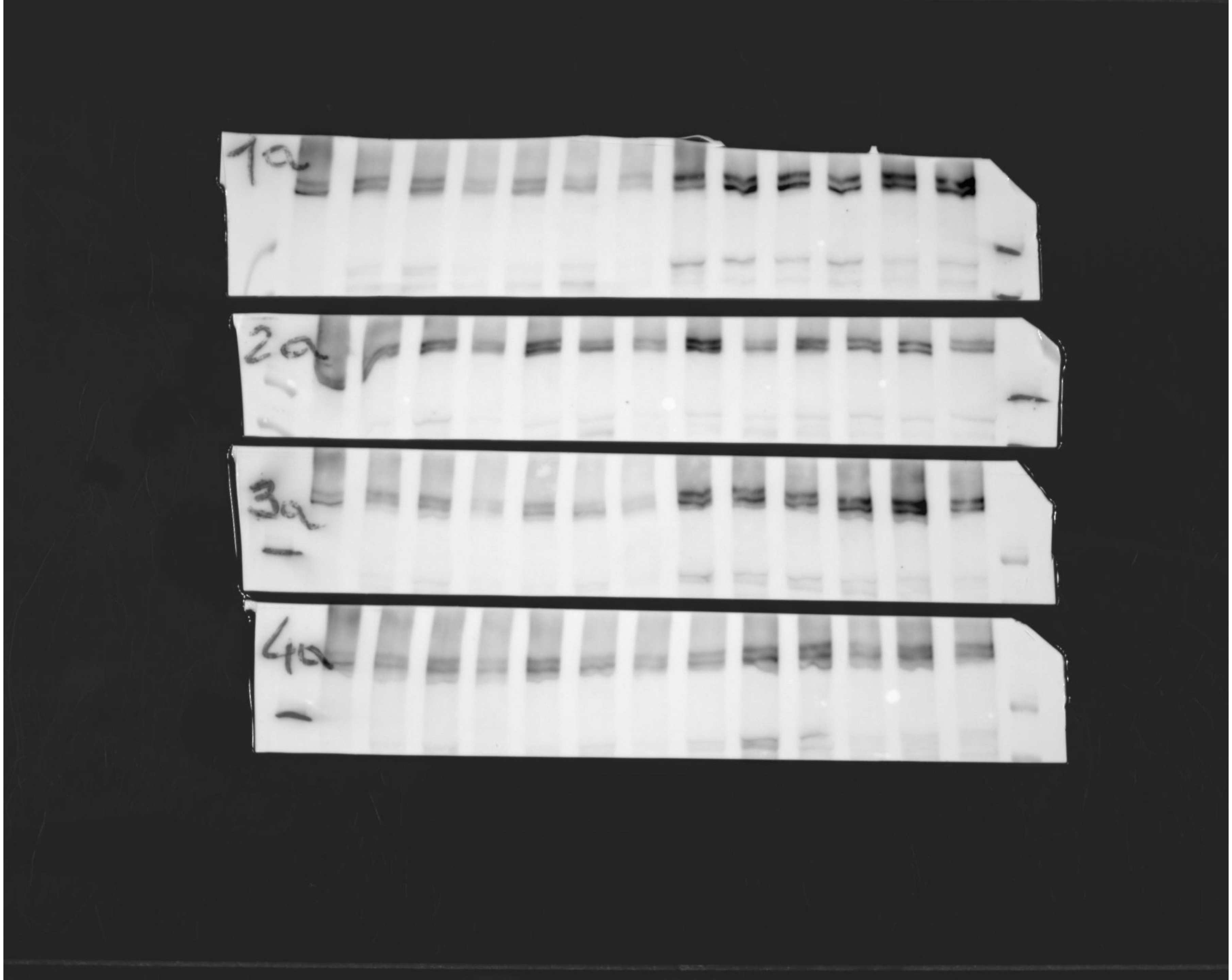

ACC fasted

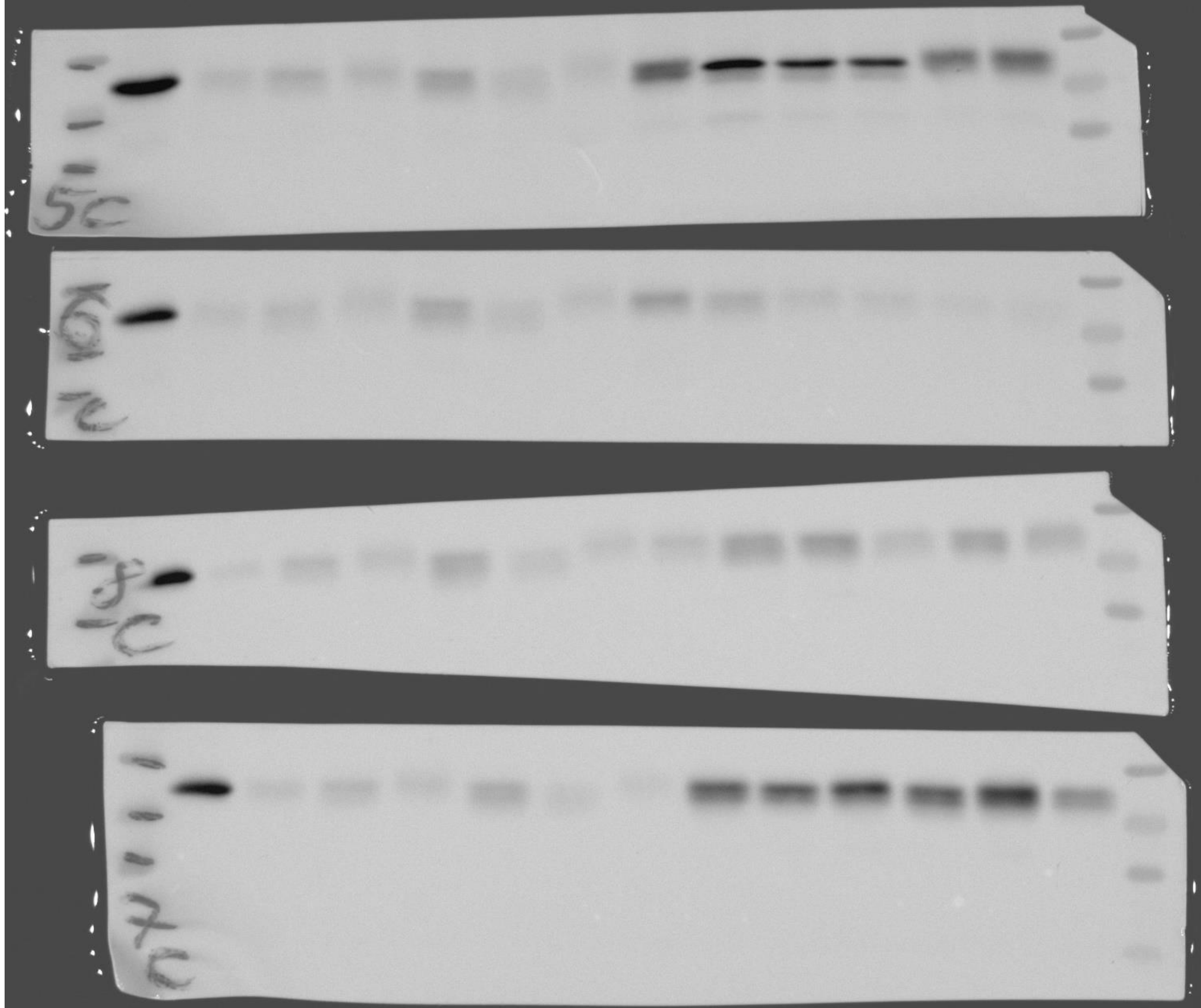

SCD1 fasted

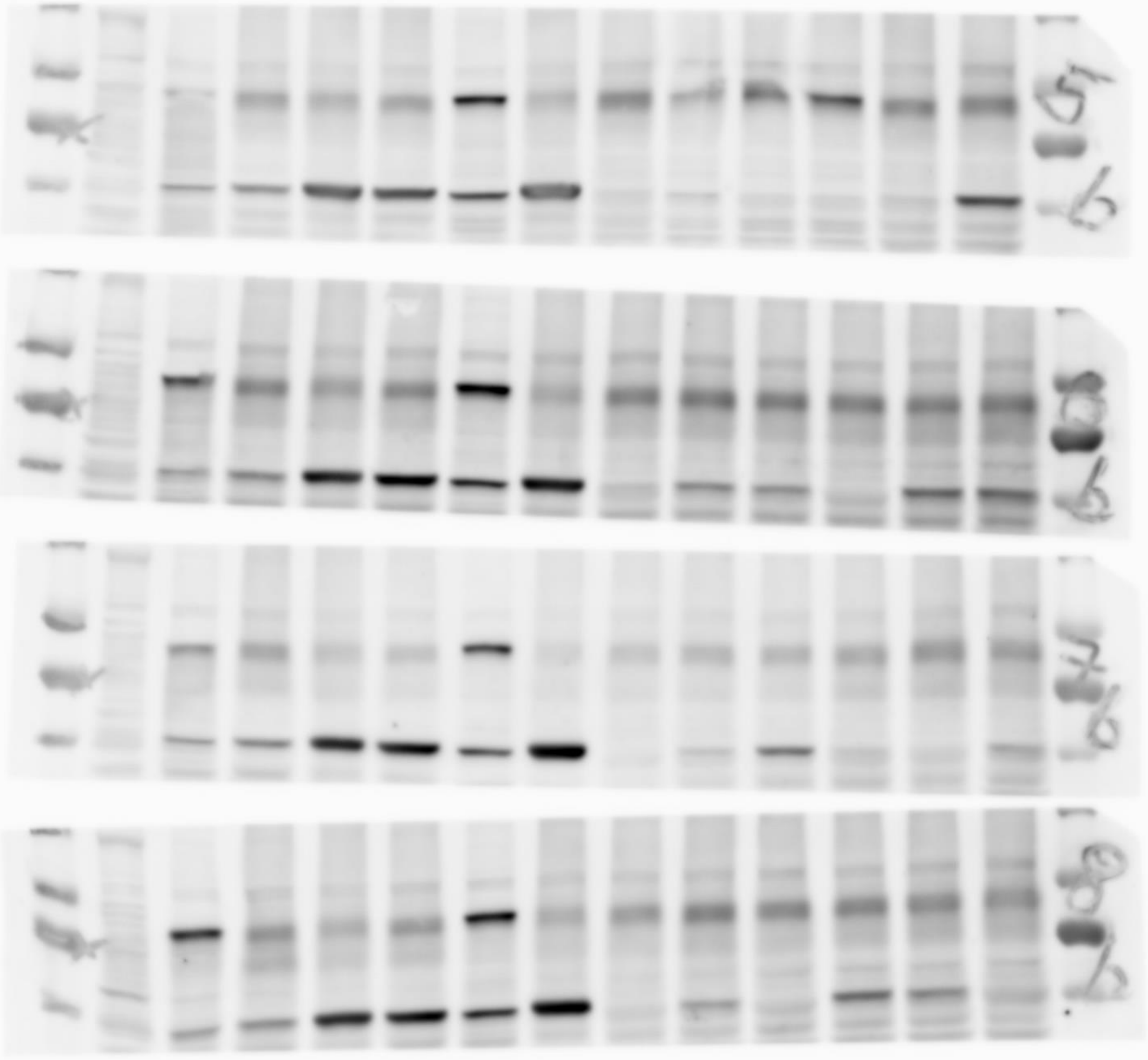

CPT1 fasted

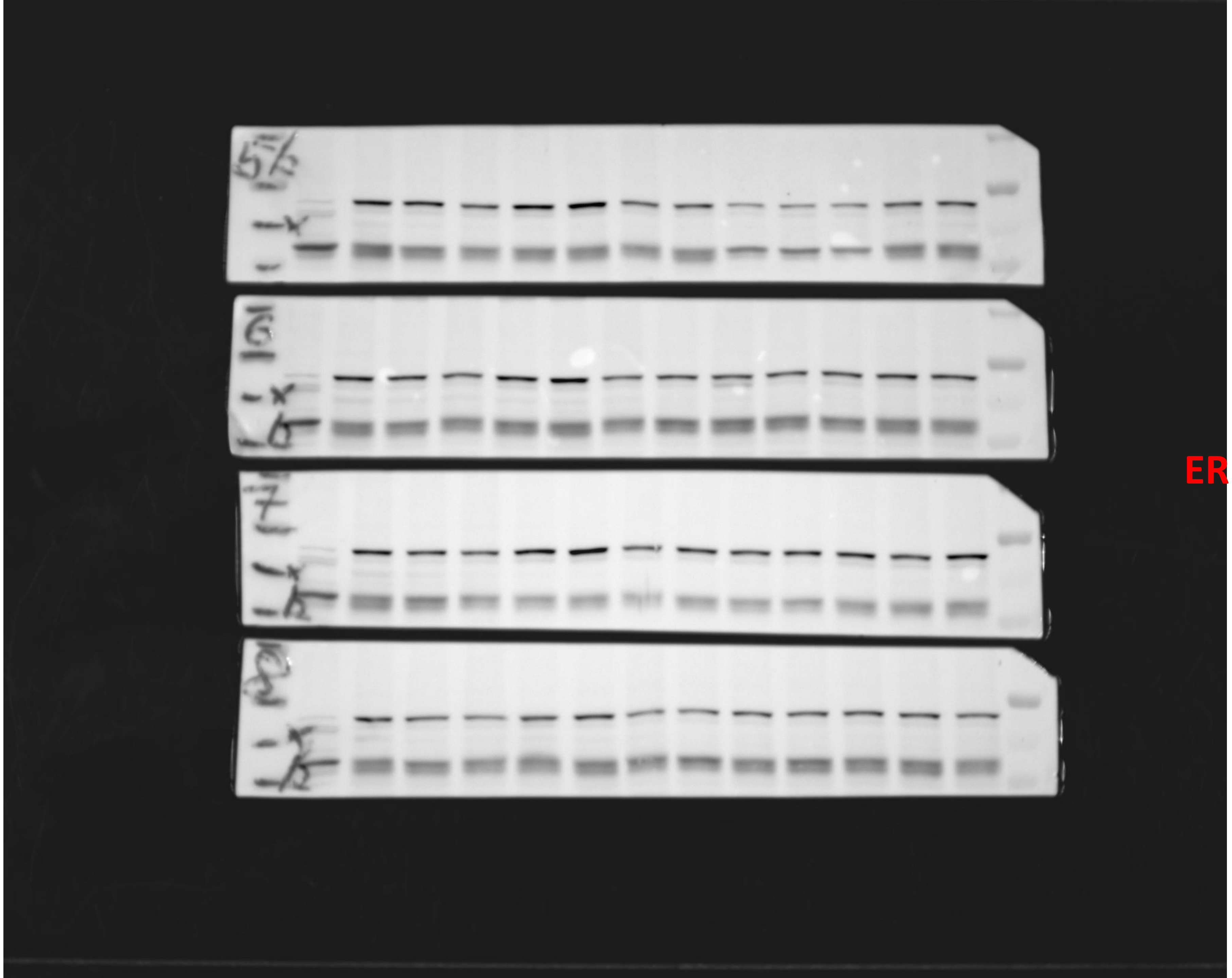

ERM fasted
