# Supplementary material for: Hepatic IR and IGF1R signaling govern distinct metabolic and carcinogenic processes upon PTEN deficiency in the liver
Source: JHEP Rep. 2024 Dec 19;7(4):101305. doi: 10.1016/j.jhepr.2024.101305 (PMC11925173; doi:10.1016/j.jhepr.2024.101305)
Supplement: Multimedia component 5 [file mmc5.pdf]

Hepatic IR and IGF1R signaling govern distinct metabolic and carcinogenic processes upon PTEN deficiency in the liver

Monika Gjorgjieva[1,*,†], **Nicolas Calo**[1,†], Cyril Sobolewski[1], Dorothea Portius[1], Jean-Luc Pitetti[2], Flavien Berthou[1], Anne-Sophie Ay[1], Marion Peyrou[1], Lucie Bourgoin[1], Christine Maeder[1], Margot Fournier[1], Marta Correia de Sousa[1], Etienne Delangre[1], Laurent Vinet[3], Xavier Montet[3], Christine Sempoux[4], Serge Nef[5], Michelangelo Foti[1]

JHEP Reports **2025**. vol. 7 | 1–13

Check for updates

Background & Aims: Hepatocyte-specific deficiency of the phosphatase and tensin homolog (PTEN) triggers steatosis and the development of hepatic tumors. The hepatoprotective effect of PTEN may partly depend on its ability to block insulin receptor (IR) and insulin-like growth factor 1 receptor (IGF1R) signaling. This study aimed to evaluate the individual/combined contributions of IR and IGF1R to hepatic metabolism and tumorigenesis induced by PTEN deficiency.

Methods: Mouse models with hepatocyte-specific deletions of *Insr, Igf1r,* or both, in addition to *Pten*, were used to investigate the distinct/combined roles of IR and IGF1R. Analyses focused on the impact of these deletions on hepatic steatosis and metabolism, whole-body adiposity, and liver tumor incidence.

Results: IR and IGF1R signaling contribute to steatosis induced by *Pten* ablation through distinct mechanisms. Hepatic IGF1R regulates hepatic glucose output and glycogen storage (2.1-fold increase in hepatic glycogen in PTEN-IGF1RKO mice [n = 10], compared with PTENKO mice [n = 7], p <0.0001). In contrast, hepatic IR exerts a stringent regulation on whole-body adiposity (4-fold increase in white adipose tissue volume in PTEN-IRKO mice [n = 5], compared with PTENKO mice [n = 6], p = 0.0004). Interestingly, triple knockout (*Insr, Igf1r*, and *Pten*) in hepatocytes of young adult mice is largely asymptomatic, indicating that PTEN deficiency exerts a major overriding control on the effects of *Insr* and *Igf1r* deletion. Furthermore, the combined loss of IR and IGF1R signaling in PTEN-deficient livers restrains liver carcinogenesis, but both receptors have individually distinct effects on the malignancy of liver cancers, with IR deficiency reducing overall cancer incidence and IGF1R deficiency promoting malignancy.

Conclusions: These findings increase our understanding of the intricate interplay between PTEN, IR, and IGF1R signaling and provide valuable insights into potential therapeutic interventions in hepatic disorders and hepatocellular carcinoma.

© 2024 The Author(s). Published by Elsevier B.V. on behalf of European Association for the Study of the Liver (EASL). This is an open access article under the CC BY license (http://creativecommons.org/licenses/by/4.0/).

Introduction

Metabolic dysfunction-associated steatotic liver disease (MASLD) encompasses hepatic disorders associated with obesity, insulin resistance, type 2 diabetes, and metabolic syndrome.[1] Its development is influenced by genetic, environmental, and lifestyle factors. The disease begins as simple steatosis, characterized by abnormal accumulation of lipid droplets in hepatocytes.[2] Selective hepatic insulin resistance, leading to uncontrolled hepatic glucose production alongside persistent lipogenesis, is commonly associated with steatosis.[3] Over time, lipotoxicity, glucotoxicity, and mitochondrial dysfunction drive hepatocyte apoptosis and inflammation, progressing to metabolic dysfunction-associated steatohepatitis (MASH).[4] This, in turn, activates hepatic stellate cells, leading to fibrosis, which may progress to cirrhosis and eventually hepatocellular carcinoma (HCC).[5]

The dogmatic view of steatosis as a harmful condition associated with insulin resistance is challenged by rodent models in which hepatic steatosis coexists with improved glucose tolerance. This phenomenon is evident in rodents fed a soy-enriched diet,[6] mice treated with corticosteroids,[7] or mice with a genetic deletion of the phosphatase and tensin homolog (PTEN) in hepatocytes.[8] PTEN is a phosphatidylinositol-3,4,5-trisphosphate 3-phosphatase that inhibits insulin receptor (IR) and insulin-like growth factor 1 receptor (IGF1R) signaling. Both receptors activate PI3K/AKT and MAPK signaling cascades, thereby controlling metabolism, mRNA translation, cell growth, and survival.[9] Supporting the negative regulatory role of PTEN in insulin/IGF-1 signaling, PTEN heterozygosity, or conditional deletion of PTEN in muscle or adipose tissue leads to an improved glucose tolerance in mice, which is mostly associated with constitutive activation of downstream effectors of these hormonal factors and an improvement in insulin sensitivity.[10,11] Particularly in the liver, the specific loss of PTEN in hepatocytes results in the sequential development of steatosis, inflammation/fibrosis, and hepatic tumors with age.[12,13] Despite this, these mice exhibit improved systemic glucose tolerance, increased muscle insulin

* Corresponding author. Address: University of Geneva, Faculty of Medicine, 1, Michel Servet, CH-1211, Geneva, Switzerland. Tel.: +41-22-37-95-506.
E-mail address: monika.gjorgjieva@unige.ch (M. Gjorgjieva).
† These authors contributed equally.
https://doi.org/10.1016/j.jhepr.2024.101305

sensitivity, lower insulin levels, and reduced adiposity. These effects have been attributed to a complex inter-organ crosstalk, mediated through hepatokines.[12,14–16]

Consistent with the role of PTEN as a negative regulator of IR signaling, hepatocyte-specific IR deletion produces a metabolic phenotype opposite to that of PTEN ablation. Mice lacking IR in hepatocytes display glucose intolerance, hyperglycemia, and an absence of steatosis.[17] The lack of IR signaling in hepatocytes has also been reported to lead to β-cell hyperplasia and insulin hypersecretion.[18] In contrast, the metabolic impact of IGF1R deletion in hepatocytes remains less explored. Pérez-Matute *et al.*[19] demonstrated that inducible IGF1R knockout mice develop insulin resistance and hepatic steatosis. IGF1R signaling has also been shown to promote hepatic regeneration[20] and cholestatic liver injury.[21] Furthermore, it has been suggested that IGF1R upregulation is an early event in hepatocarcinogenesis, where it maintains high proliferation and cancer stemness in transformed hepatocytes.[22] However, this remains to be confirmed *in vivo*. HCC is one of the leading causes of cancer-related deaths worldwide.[23] The insulin/IGF-1 signaling is one of the most deregulated pathways in human cancers.[24] Both are found overexpressed or deregulated in various *in vitro* and *in vivo* models of HCC.[25–27] Overactivation of the IGF1R is one of the major hallmarks of hepatocarcinogenesis.[28] In support of this, IGF1R blockade results in decreased hepatoma cell migration *in vitro*.[29]

In summary, in hepatic steatosis and HCC, PTEN expression is reduced/lost, whereas signaling through IR and IGF1R is elevated. Understanding the interplay between PTEN, IR, and IGF1R is crucial for elucidating hepatic disease mechanisms. This study examines how IR and IGF1R signaling impact hepatic and systemic metabolism and contribute to the spontaneous development and progression of hepatic steatosis and tumors driven by PTEN deficiency.

Materials and methods

Animals

Generation of mice with hepatocyte-specific deletion of *Insr, Igf1r*, and *Pten* using the Cre/loxP system with *Cre-recombinase* under control of the albumin promoter has been described previously.[12,16,17,20] We crossbred mice carrying loxP sites for *Insr*, *Igf1r*, and *Pten* with mice having the *AlbCre* gene and obtained *AlbCre-Pten*$^{flox/flox}$ (PTENKO), *AlbCre-Pten*$^{flox/flox}$/*Insr*$^{flox/flox}$ (PTEN-IRKO), *AlbCre-Pten*$^{flox/flox}$/*Igf1r*$^{flox/flox}$ (PTEN-IGF1RKO), and *AlbCre-Pten*$^{flox/flox}$/*Insr*$^{flox/flox}$/*Igf1r*$^{flox/flox}$ (PTEN-IR-IGF1RKO; triple-KO). Floxed mice (*Pten*$^{flox/flox}$ and/or *Insr*$^{flox/flox}$ and/or *Igf1r*$^{flox/flox}$, negative for the *AlbCre* gene) were used as control (CTRL). Mice were euthanised at the age of 4 months (metabolic assessment, fed or fasted state) or 12 months (hepatocarcinogenesis assessment, fed state) by decapitation under isoflurane anesthesia. Blood and tissues were collected and stored at -80 °C. All the mice that were used for experimentation were male, as MASLD has a higher prevalence in men. The genetic background of the mice was C57BL/6.

Study approval

Animals were cared for and housed according to the Swiss guidelines for animal experimentation and ethically validated by the Geneva Health head office (experimental authorizations GE/60/14, GE/63/15, and GE/43/14).

Detailed information on the materials and methods used are provided in the Supplementary material.

Results

Hepatic IR signaling, but not IGF1R, regulates liver steatosis status in conjunction with PTEN

We generated C57BL/6 mice with constitutive hepatocyte-specific deletion of *Pten* (PTENKO), combined with deletion of *Insr* (PTEN-IRKO), *Igf1r* (PTEN-IGF1RKO), or both receptors (PTEN-IR-IGF1RKO; triple-KO). The deletions resulted in near complete absence of PTEN expression in the hepatocytes of PTENKO, PTEN-IRKO, PTEN-IGF1RKO, and triple-KO mice; absence of IR expression in hepatocytes of PTEN-IRKO and triple-KO mice; and absence of IGF1R expression in hepatocytes of PTEN-IGF1RKO and triple-KO mice (Fig. S1A). Moreover, PTEN-IRKO and triple-KO mice had a drastic reduction of IR-B isoform in the liver, whereas the IR-A isoform was only slightly decreased (Fig. S1B). Mice carrying floxed alleles, but not the AlbCre recombinase, were used as controls (CTRL).

PTENKO mice develop hepatomegaly associated with extensive steatosis early in adulthood.[12–14,16] *Ex vivo* assessment of hepatomegaly was performed under fed and fasted conditions. Under fed conditions, PTENKO and PTEN-IGF1RKO mice had similar liver weights, significantly heavier than those of CTRL mice, whereas PTEN-IRKO and triple-KO mice had normal liver weights, comparable to those of CTRL mice (Fig. 1A, left panel). Surprisingly, under fasted conditions, PTEN-IRKO, PTEN-IGF1RKO, and triple-KO mice all had significantly lower liver weights compared with PTENKO mice, with livers of the triple-KO group reaching the same weight as CTRL livers (Fig. 1A, left panel). Liver weight in PTEN-IRKO and triple-KO animals remained unchanged between fasted and fed conditions. Similar results were observed when liver weight was expressed as a percentage of body weight (Fig. 1A, right panel). Therefore, both IR and IGF1R signaling can drastically affect liver volume in PTEN-deficient mice, and such outcome is dependent on the nutritional status.

Histological analysis of liver sections in the fed state (Fig. 1B), together with intrahepatic triglyceride (TG) content (Fig. 1C), revealed that the severe steatosis present in PTENKO mice was greatly reduced in the absence of IR (PTEN-IRKO mice) in the fed state; however, deletion of both receptors appears to have cumulative effects on steatosis clearance, as observed in the triple-KO group. In contrast, IGF1R deletion did not reduce hepatic steatosis (Fig. 1B and C). Therefore, in a PTEN deficiency context under fed conditions, IR signaling is a critical mediator of hepatic lipid status. Surprisingly, under fasted conditions, PTENKO mice had hepatic TG levels comparable to those of CTRL mice, whereas PTEN-IGF1RKO and PTEN-IRKO mice had significantly higher hepatic TG levels (Fig. 1C).

Although not significant, a tendency for increased hepatic cholesteryl esters was observed in PTEN-IRKO under fed conditions, suggesting that this lipid species is an important component of the remnant lipid droplets in the hepatocytes of this group (Fig. 1D). In contrast, hepatic ketone bodies were significantly increased in PTENKO and PTEN-IGF1RKO mice,

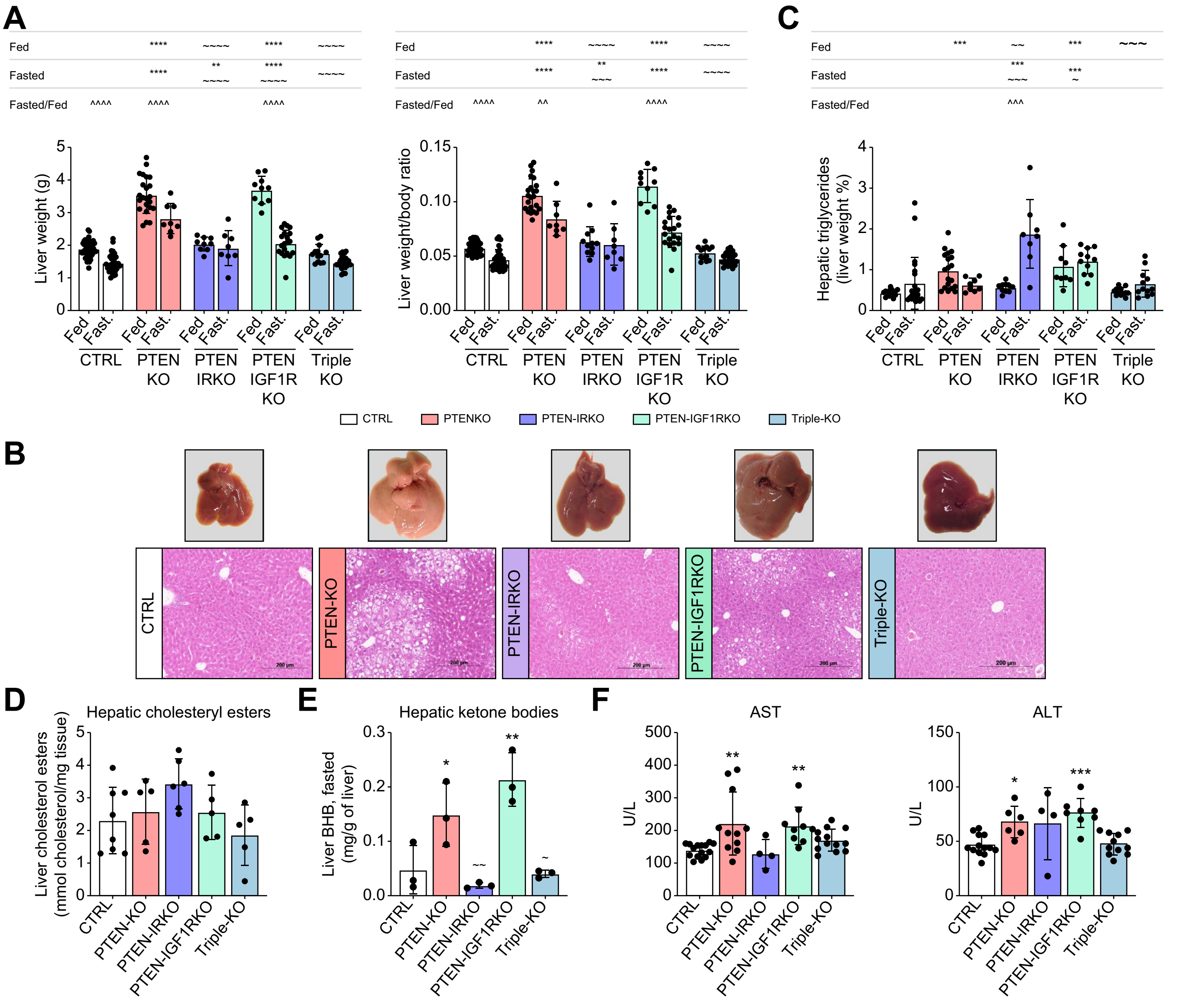

Fig. 1. Hepatic IR signaling contributes to PTEN deficiency-induced steatosis. (A) *Ex vivo* assessment of the liver weight (left panel, fed/fasted state: CTRL n = 48/n = 33, PTENKO n = 23/n = 8, PTEN-IRKO n = 9/n = 8, PTEN-IGF1RKO n = 10/n = 20, and triple-KO n = 13/n = 25) and liver weight presented as ratio to body weight (right panel). (B) Representative anatomy and histology (H&E staining) of hepatic tissues (fed conditions). (C) Hepatic triglyceride content (fed/fasted state: CTRL n = 15/n = 23, PTENKO n = 19/n = 8, PTEN-IRKO n = 10/n = 8, PTEN-IGF1RKO n = 9/n = 11, and triple-KO n = 12/n = 12). (D) Hepatic cholesteryl ester content (fed state, n = 5–6/group). (E) Ketone body (β-hydroxybutyrate) content (n = 3/group). (F) Circulating levels of AST (CTRL n = 15, PTENKO n = 11, PTEN-IRKO n = 4, PTEN-IGF1RKO n = 9, and triple-KO n = 13) and ALT (CTRL n = 13, PTENKO n = 6, PTEN-IRKO n = 4, PTEN-IGF1RKO n = 8, and triple-KO n = 11) from 4-month-old mice analyzed after sacrifice. Values are mean ± SD. One-way ANOVA was performed. Values were considered significant compared with CTRL (*) and PTENKO (~): */~ *p* ≤0.05, **/~~ *p* ≤0.01,***/~~~ *p* ≤0.001, or ****/~~~~ *p* ≤0.0001. ALT, alanine transaminase; AST, aspartate transaminase; IR, insulin receptor; PTEN, phosphatase and tensin homolog.

consistent with steatosis (Fig. 1E). Circulating aspartate transaminase (AST) and alanine transaminase (ALT) were increased in PTENKO and PTEN-IGF1RKO mice, but not in PTEN-IRKO and triple-KO mice, suggesting that disruption of IR signaling might attenuate hepatic injury associated with PTEN deficiency (Fig. 1F), possibly because of the reduction in hepatic steatosis in these IR-deficient groups.

Collectively, these data indicate that the IR-PTEN signaling axis in hepatocytes is a master regulator of hepatic size, steatosis, and injury. In contrast, IGF1R signaling had only a minor effect on these parameters under PTEN-deficient conditions in the fasting state.

Hepatic IR/PTEN signaling controls lipid and cholesterol synthesis in the liver

Further analyses of explanted liver tissue were performed to distinguish the specific processes of hepatic lipid metabolism regulated by IR and IGF1R. As expected, PTEN deletion led to an increase in hepatic *de novo* lipogenesis (DNL) with significant increases in the expression of *Fasn*, *Pparg*, and *Scd1* and increased lipid trafficking (*Cd36*) under fed conditions (Fig. 2A, upper panels). DNL-related genes were no longer significantly upregulated in PTEN-IRKO livers, compared with PTENKO livers, and an even further reduction in expression was

observed in triple-KO livers (Fig. 2A). In contrast, PTEN-IGF1RKO livers showed DNL gene expression comparable to that of PTENKO livers. Similar expression profiles were observed in the fasted state, and the results were confirmed at the protein level (Fig. 2B).

However, deficient insulin signaling in *Pten*-knockout hepatocytes (PTEN-IRKO) led to an increased expression of factors involved in hepatic cholesterol metabolism (compared with CTRL) under fed conditions (*Abcg5, Hmgcr, Lxrb*, and *Hmgcs1*; Fig. S2, upper panels). In contrast, IR loss no longer induced cholesterol synthesis under fasted conditions and led to a striking decrease in *Hmgcs1* expression (Fig. S2, bottom panels). Triple-KO livers showed similar trends to PTEN-IRKO livers in the expression of genes involved in cholesterol metabolism in both fed and fasted conditions. Deletion of IGF1R in *Pten*-deficient hepatocytes (PTEN-IGF1RKO) did not significantly affect cholesterol metabolism in either the fasted or fed state, when compared with PTENKO. These findings indicate that unrepressed IR signaling in the absence of PTEN is the major driver of deregulated lipid and cholesterol metabolism in the liver under these conditions, whereas the absence of IGF1R does not affect these processes.

Finally, assessment of hepatic β-oxidation revealed an upregulation of *Acox1* in PTEN-IRKO livers under fed and fasted conditions, as compared with PTENKO livers (Fig. S3A). PTEN-IGF1RKO mice had more drastic changes in β-oxidation compared with PTENKO mice, as *Acat2* and *Cpt1* were upregulated under fed conditions, and a similar trend was also observed for *Acat1* and *Pgc1a* (Fig. S3A). The increase in CPT1 in PTEN-IGF1RKO livers was confirmed at the protein level (Fig. S3B). This effect of IGF1R on hepatic β-oxidation did not persist under fasted conditions (Fig. S3A and B).

Therefore, IR deficiency led to a decrease in hepatic lipid synthesis, resulting in less hepatic steatosis, whereas IGF1R deficiency increased lipid oxidation and ketogenesis under fed conditions. However, the latter was not sufficient to alleviate hepatic steatosis in PTEN-IGF1RKO mice (Fig. 1B and C).

Both hepatic IR/PTEN and IGF1R/PTEN signaling contribute to the control of peripheral fat storage

Having established that IR and IGF1R signaling are responsible for the regulation of different aspects of hepatic lipid metabolism, we further investigated the effects of these hepatic deletions at the systemic level. No significant changes in body weight were detected in the five groups under fed conditions (Fig. 3A). However, PTEN-IGF1RKO mice had a significantly lower body weight under fasted conditions (Fig. 3A). Furthermore, both PTEN-IGF1RKO and PTEN-IRKO animals showed an exacerbation of body weight loss under fasted conditions, compared with fed animals with the same genotype, indicating that liver-specific IR and IGF1R have the potential to regulate whole-body metabolic homeostasis, particularly under fasted conditions (Fig. 3A). EchoMRI (LLC, Huston, USA http://www.echomri.com/) analysis under fed conditions revealed that triple-KO animals had significantly lower lean mass than PTENKO animals, and a similar trend was observed in the PTEN-IGF1RKO group (Fig. 3B). Intriguingly, when the lean mass was expressed as a percentage of total body mass of the animal, PTEN-IRKO mice had a significantly lower ratio compared with CTRL and PTENKO mice (Fig. S4A). A similar trend was observed in the triple-KO group, whereas the opposite was observed in the PTEN-IGF1RKO group.

Furthermore, quantification of total fat mass by EchoMRI revealed a trend toward a decrease in PTENKO mice, compared with CTRL mice, whereas PTEN-IRKO and triple-KO mice had significantly higher fat mass levels, compared with PTENKO mice (Fig. 3B). When fat mass was expressed as a percentage of body weight, all three mutants (PTEN-IRKO, PTEN-IGF1RKO, and triple-KO) had higher fat mass levels, compared with PTENKO mice (Fig. S4A). Fat mass determined by echoMRI includes the lipids in the liver; therefore, to determine the adiposity levels in the mutants without taking into account hepatic lipid storage, a CT scan was used to measure the volume of all peripheral fat depots.

In vivo CT scan imaging of total white adipose tissue (WAT) showed that the volume of fat depots was significantly reduced in PTENKO mice compared with CTRL mice (Fig. 3C and D and Fig. S4B). Conversely, PTEN-IRKO mice exhibited fat depots similar to those of CTRL mice, indicating that hepatocyte-specific deletion of IR in a PTENKO setting restores WAT volume to wild-type levels (Fig. 3C and D and Fig. S4B). Similar results were obtained in PTEN-IGF1RKO and triple-KO mice; however, the increase in WAT volume was less than that in CTRL and PTEN-IRKO mice (Fig. 3C and D and Fig. S4B).

Furthermore, CT imaging showed that brown adipose tissue (BAT) volume tended to increase in PTENKO and in PTEN-IRKO mice compared with CTRL mice, although not significantly (Fig. S5A). BAT size seems to be primarily dependent on IGF1R signaling, as PTEN-IGF1RKO mice presented a significantly lower volume of BAT (Fig. S5A). Deletion of both receptors led to an intermediate phenotype similar to that of CTRL mice (Fig. S5A). Lipid droplet area quantification in histological sections of BAT stained with H&E confirmed that PTEN-IGF1RKO and triple-KO mice had significantly lower amounts of lipids in BAT (Fig. S5B and C). This striking decrease in adiposity under IGF1R-deficient conditions could be attributed to an increased usage of fatty acids in the BATs of these mice. *Ucp1* (uncoupling protein 1) mRNA levels indicated a tendency for increased expression in the BAT of PTENKO and triple-KO animals, whereas PTEN-IRKO and, more strikingly, PTEN-IGF1RKO mice both had significant fourfold to fivefold increase in the expression of this marker (Fig. S5D). Similar patterns of expression were observed with *Pgc1a* (PPARG coactivator 1α). UCP1 protein increase in PTEN-IRKO mice, and even more in PTEN-IGF1KO mice, was also confirmed through immunofluorescence (Fig. S5E).

Despite the marked effects observed in adipose tissue, LabMaster (TSE systems, Germany https://www.tse-systems.com/) analysis revealed no significant differences in physical activity or energy expenditure between the groups (Fig. 3E and Fig. S6A and B). However, a slight tendency to increase these parameters during the day was observed in PTEN-IGF1RKO and triple-KO mice (Fig. S6A and B). In addition, triple-KO mice consumed more food in 24 h (Fig. 3F and Fig. S6C). Furthermore, PTEN-IGF1RKO and triple-KO mice showed an increase in the respiratory exchange ratio (RER; VCO_2/VO_2), indicating a metabolic shift from fat oxidation to glucose consumption (Fig. 3G and Fig. S6D–F).

Finally, leptin and adiponectin, both decreased in PTENKO mice, were upregulated in the PTEN-IRKO group, indicating an increased endocrine activity of WAT (Fig. 3H). In contrast,

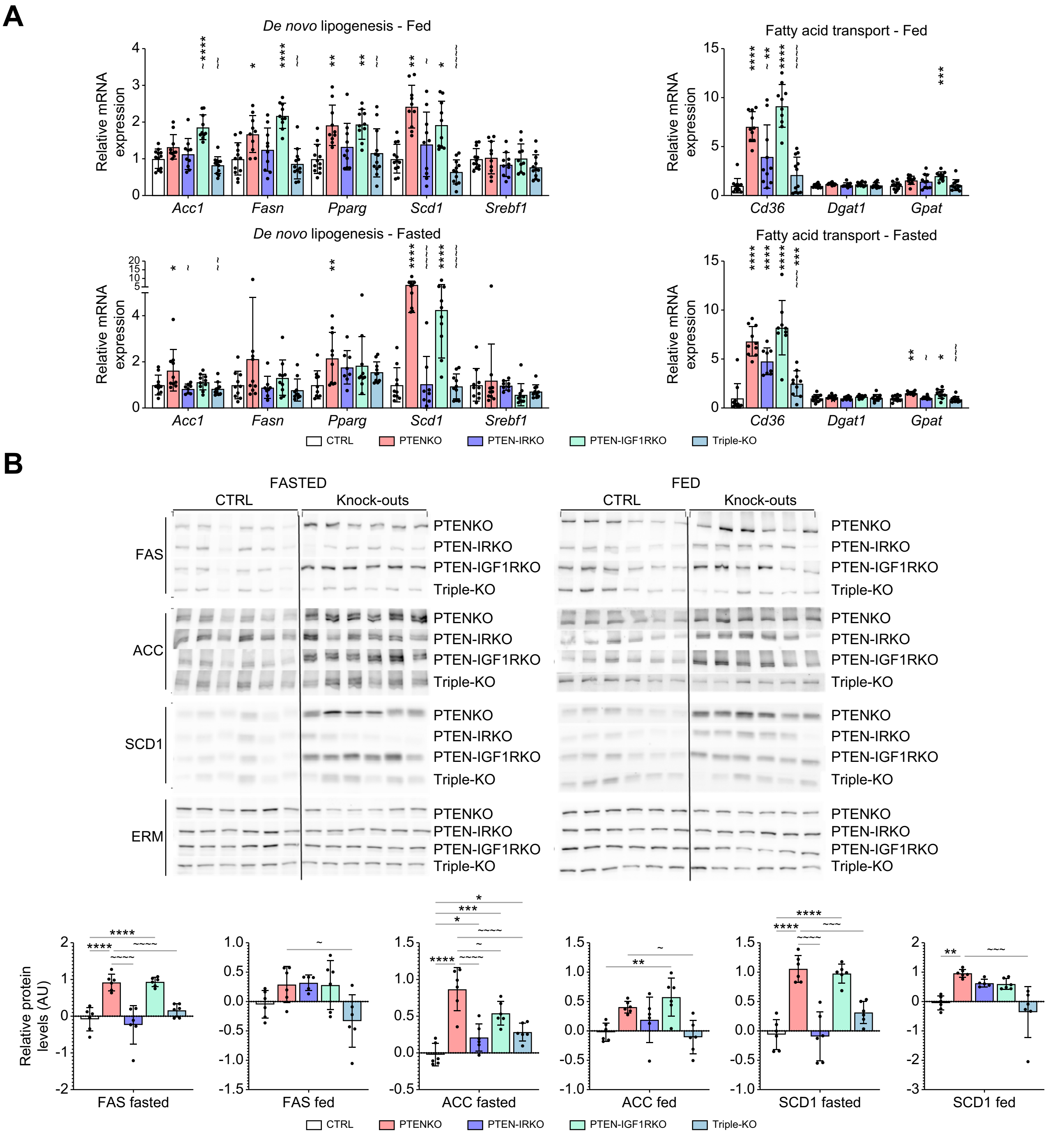

Fig. 2. IR and IGF1R signaling differentially regulate lipid metabolism in the liver. (A) Hepatic mRNA expression of enzymes involved in fatty acid synthesis (*Acc1*, *Fasn*, *Pparg*, *Scd1*, and *Srebf1*) and transport (*Cd36*, *Dgat*, and *Gpat*) (fed/fasted state: CTRL n = 12/n = 10, PTENKO n = 10/n = 10, PTEN-IRKO n = 10/n = 8, PTEN-IGF1RKO n = 10/n = 10, and triple-KO n = 12/n = 10). (B) Western blots and quantifications of hepatic FAS, ACC, and SCD1 protein expression (n = 6/group) and ERM (for loading control and normalization). Values are mean ± SD. One-way ANOVA was performed. Values were considered significant compared with CTRL (*) and PTENKO (~): */~ p ≤0.05, **/~~ p ≤0.01, ***/~~~ p ≤0.001, or ****/~~~~ p ≤0.0001. ACC, acetyl-CoA carboxylase; ERM, ezrin/radixin/moesin; FAS, fatty acid synthase; IGF1R, insulin-like growth factor 1 receptor; IR, insulin receptor; PTEN, phosphatase and tensin homolog; SCD1, stearoyl-CoA desaturase 1.

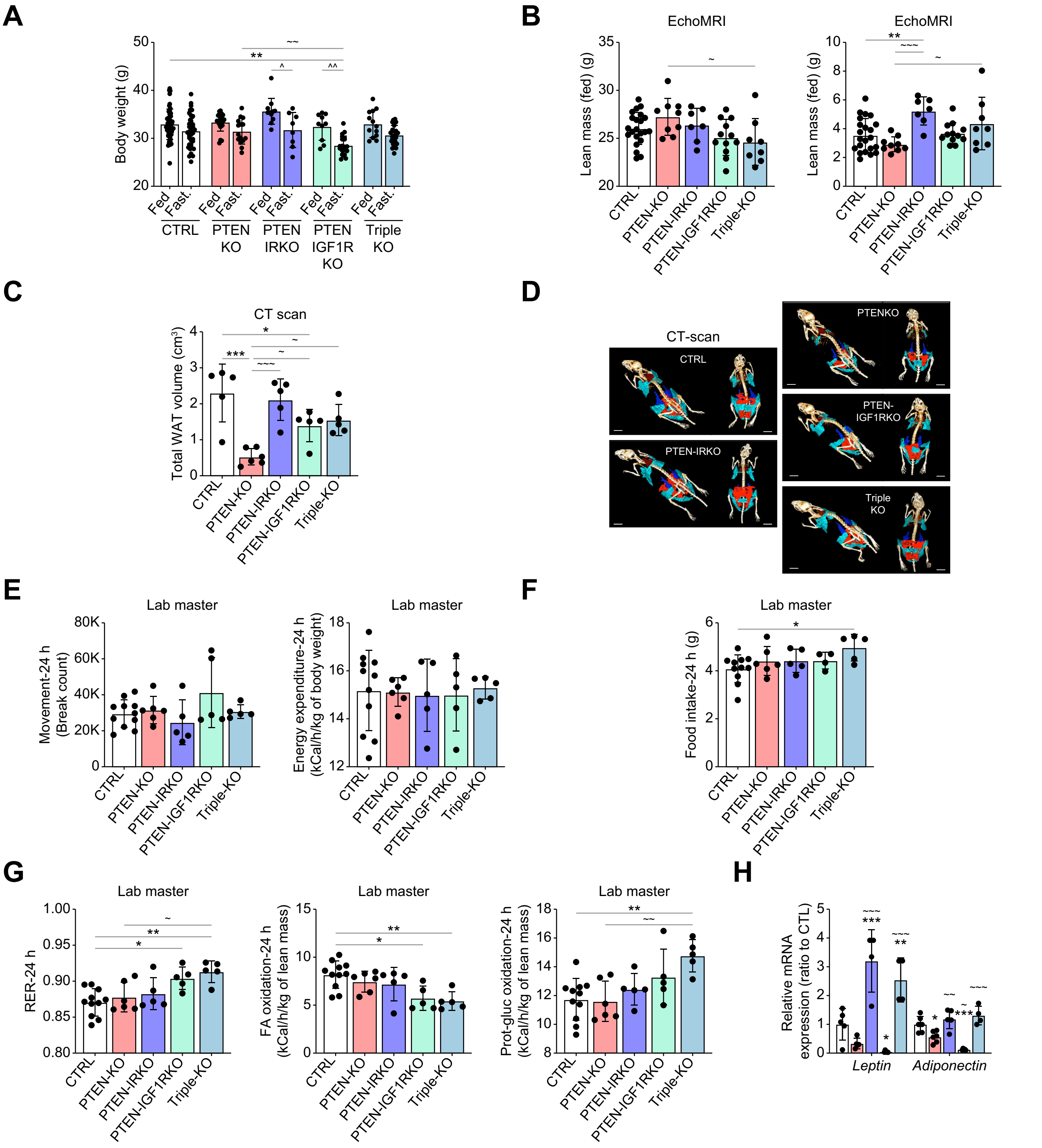

Fig. 3. Hepatic IR and IGF1R signaling differentially influence adipose tissue size. (A) Body weight of 4-month-old mice under fed and fasted conditions (fed/fasted state: CTRL n = 48/n = 38, PTENKO n = 23/n = 15, PTEN-IRKO n = 11/n = 8, PTEN-IGF1RKO n = 10/n = 20, and triple-KO n = 13/n = 25). (B) EchoMRI analysis of the lean mass (left panel) and fat mass (right panel) (CTRL n = 22, PTENKO n = 9, PTEN-IRKO n = 7, PTEN-IGF1RKO n = 12, and triple-KO n = 8). (C) CT scan assessment of total WAT volume (n = 5–6/group) and (D) representative reconstruction of CT scan imaging of the mice under fed conditions. (E) Locomotor movement and energy expenditure, (F) food consumption, (G) RER and substrate (FA *vs.* carbohydrate/protein) oxidation rates assessed via LabMaster metabolic cages in 4-month-old CTRL (n = 11), PTENKO (n = 6), PTEN-IRKO (n = 5), PTEN-IGF1RKO (n = 5), and triple-KO (n = 5) mice under fed conditions. (H) eWAT mRNA expression of the adipokines leptin and adiponectin (n = 4–6/group). Values are represented as mean ± SD. One-way ANOVA was performed. Values were considered significant when compared with CTRL (*) and PTENKO (~): */~ *p* ≤0.05, **/~~ *p* ≤0.01,***/~~~ *p* ≤0.001, or ****/~~~~ *p* ≤0.0001. CT, computed tomography; FA, fatty acid; IGF1R, insulin-like growth factor 1 receptor; IR, insulin receptor; PTEN, phosphatase and tensin homolog; RER, respiratory exchange ratio; WAT, white adipose tissue.

PTEN-IGF1RKO mice showed a drastic reduction in the expression of these genes.

These observations indicate that both IR and IGF1R signaling are required for the crosstalk between the liver and the peripheral adipose tissue; however, IR has a stronger effect on WAT size, whereas IGF1R affects BAT size.

Hepatic IGF1R signaling is an important contributor to whole-body glucose homeostasis under PTEN-deficient conditions

Hepatic glucose output (HGO) is severely inhibited in PTEN-deficient hepatocytes.[14] In addition, hepatic *Pten* deletion induces a crosstalk between the liver and the skeletal muscle that promotes increased muscle insulin sensitivity and glucose uptake.[14,16] These effects of hepatic *Pten* deletion resulted in greatly improved glucose tolerance in PTENKO mice, even though hepatic steatosis is usually associated with insulin and glucose intolerance.[14] Thus, hepatic PTEN deficiency has the capacity to rewire glucose metabolism at the whole-body level.

To investigate the respective roles of IR and IGF1R signaling in this process, glucose tolerance tests (GTTs) were performed. GTTs revealed that deletion of the hepatic IGF1R signaling in both PTEN-IGF1RKO and triple-KO mice prevented the glucose hyper-tolerance observed in PTENKO mice, whereas dual IR/PTEN deletion showed no significant difference from PTEN deficiency alone (Fig. 4A). PTEN deficiency in the liver is associated with hypoglycemia and low insulin secretion (Fig. 4B). Plasmatic glucose levels were normalized in PTEN-IRKO and triple-KO mice under fasted conditions, whereas PTEN-IGF1RKO mice maintained glycemia similar to that of PTENKO mice (Fig. 4B). Insulinemia was increased to levels similar to those in CTRL mice in both the fed and fasted states, with the exception of hepatic IGF1R deficiency, which restored normal circulating insulin levels only in the fed state but not in the fasted state (Fig. 4B). Increased insulinemia in these mutants (compared with PTENKO mice) could explain the increased adiposity. Triple-KO and PTEN-IGF1RKO mice showed increased glucagon levels under fed conditions, whereas there were no statistically significant changes between groups under fasted conditions (Fig. 4B).

Pyruvate tolerance tests (PTTs) were performed to investigate HGO in the absence of IR and/or IGF1R. Consistent with GTT observations, HGO was significantly reduced in PTENKO mice. HGO was slightly but not significantly restored when IR signaling was defective in hepatocytes, but a tendency to increase was observed. However, deletion of the IGF1R increased HGO to levels comparable to those in CTRL mice (Fig. 4C). HGO was also restored to CTRL levels in triple-KO mice. RNA expression levels of the gluconeogenic genes *G6pc* and *Pck1* under fasted conditions remained unaffected, except for *Pck1*, which was decreased in PTEN-IGF1RKO mice under fasted conditions (Fig. S7). No significant changes in gene expression were observed in the fed state (Fig. S7).

Hepatic glycogen content under fed conditions was similar between the CTRL, PTENKO, PTEN-IRKO, and triple-KO groups, except for PTEN-IGF1RKO, which had a higher glycogen concentration compared with PTENKO (Fig. 4D). This increase in glycogen explains, at least in part, the increased liver weight observed under fed conditions (Fig. 1A) and highlights IGF1R as an important player in glycogen metabolism. As expected, glycogen levels were not detectable in mice after overnight fasting (data not shown). Finally, analyses of critical effectors of the glycolysis pathway further indicate that in PTEN-deficient livers, *Pklr* expression is highly upregulated under fasted and fed conditions, along with *Gck* and *Ladha* under fed conditions (Fig. 4E), and a similar expression pattern was found in PTEN-IGF1RKO livers, indicating that IGF1R may not be involved in glycolysis regulation. In contrast, IR deficiency normalized the expression of *Ladha*, *Pdha*, and *Pklr*, demonstrating the relevance of IR in hepatic glycolysis (Fig. 4E).

Therefore, hepatic IGF1R signaling abrogation had a striking effect on glucose tolerance and HGO. Accordingly, similar effects were observed with a combined deletion of both receptors. Furthermore, IGF1R deficiency dramatically increased liver glycogen content. Nevertheless, glycolysis remained unaffected by IGF1R deficiency. However, our data indicate that glycolysis seems to be under the regulation of IR signaling, suggesting that both receptors influence hepatic glucose, but they are involved in the regulation of distinct axes.

Deletion of hepatic IR and IGF1R differentially affects the development of liver cancer induced by hepatic PTEN deletion

The IR and IGF1R signalization axes are activated in various *in vitro* models of HCC and in biopsies from patients with HCC. We summarized the previously published studies on IR and IGF1R and HCC in Table S1, and the main message of this literature review is that both receptors, together with their respective ligands, have a pro-oncogenic role in HCC.

Furthermore, *in silico* analyses of *INSR* and *IGF1R* expression in HCC biopsies (LIHC–TCGA patient cohort) showed a trend toward increased RNA expression of both receptors in the cancerous biopsies compared with noncancerous livers (Fig. 5A). Further investigation of *INSR* and *IGF1R* mRNA expression was performed in four different Gene Expression Omnibus (GEO) datasets with nontumoral hepatic tissue and paired HCC biopsy from patients (Fig. S8A). The pooled analysis from the four datasets revealed that *INSR* expression can be both strongly decreased or increased in HCC biopsy, compared with the patient's nontumoral liver (Fig. S8A and B, left panels). However, the frequency distribution revealed a higher number of patients with increased *INSR* expression (Fig. S8B). The same analysis applied to *IGF1R* expression patterns showed that there was an almost equal number of patients overexpressing and underexpressing *IGF1R* in HCC biopsy (Fig. S8A and B). When the expression of *INSR* and *IGF1R* was separated according to the staging of the HCC samples, no significant changes in *INSR* expression were observed (Fig. 5B). In contrast, *IGF1R* mRNA expression gradually increased at all stages, although this increase did not reach statistical significance (Fig. 5B). Consistently, patients with high *IGF1R* expression (10% of the patients with the highest gene expression *vs*. 90% of the rest of the patients) have a worse survival prognosis (Fig. 5C). Finally, to understand the molecular patterns occurring in patients with high *IGF1R* expression, biopsy data from patients with HCC obtained from the GSE36379 dataset were separated into the top 20% of patients expressing the highest levels of *IGF1R* and the remaining patients with the lowest expression levels (80% of

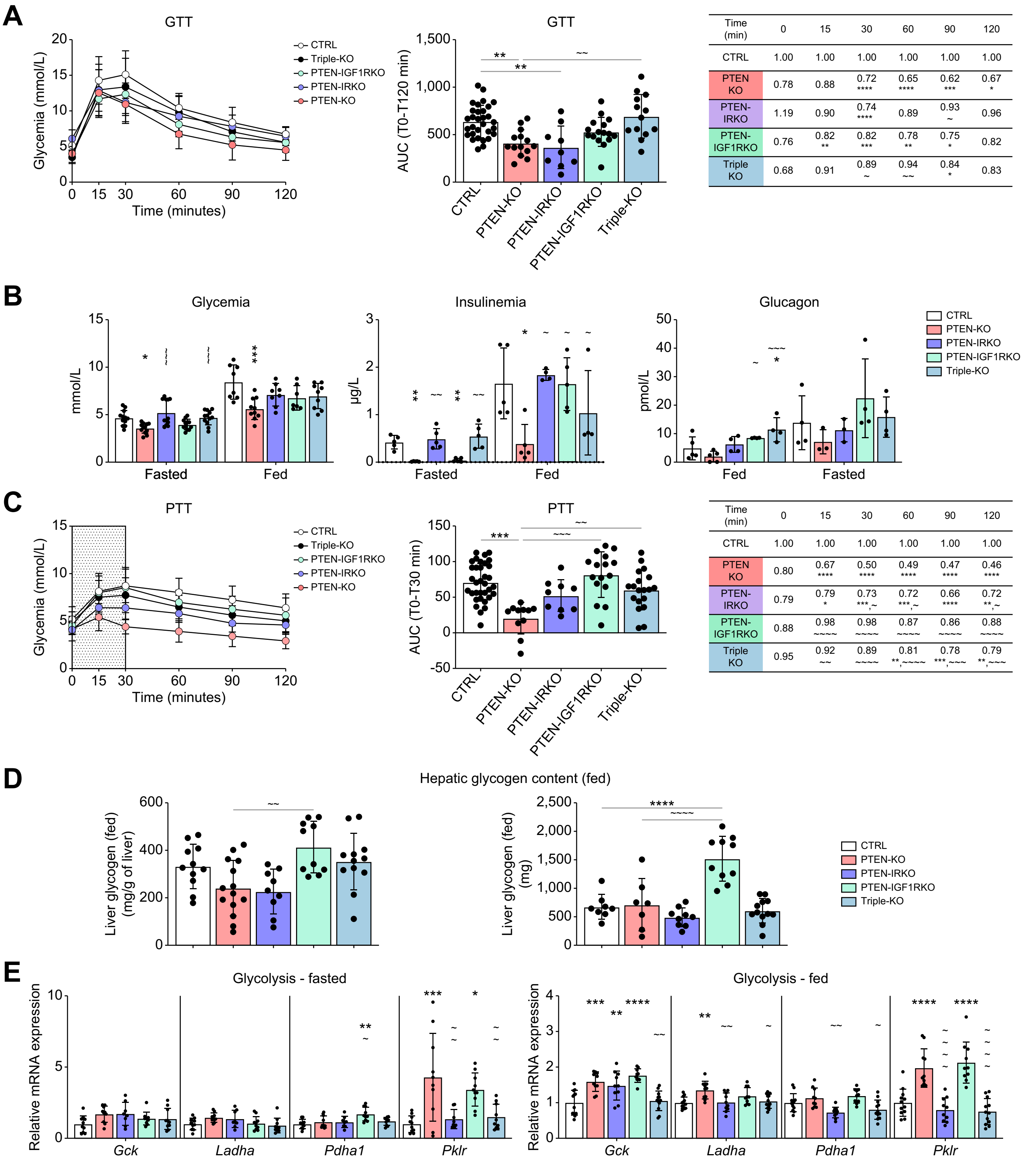

(A)

Time (min)	0	15	30	60	90	120
CTRL	1.00	1.00	1.00	1.00	1.00	1.00
PTEN KO	0.78	0.88	0.72 ****	0.65 ****	0.62 ***	0.67 *
PTEN-IRKO	1.19	0.90	0.74 ****	0.89	0.93 ~	0.96
PTEN-IGF1RKO	0.76	0.82 **	0.82 ***	0.78 **	0.75 *	0.82
Triple KO	0.68	0.91	0.89 ~	0.94 ~~	0.84 *	0.83

(C)

Time (min)	0	15	30	60	90	120
CTRL	1.00	1.00	1.00	1.00	1.00	1.00
PTEN KO	0.80	0.67 ****	0.50 ****	0.49 ****	0.47 ****	0.46 ****
PTEN-IRKO	0.79	0.79 *	0.73 ***,~	0.72 ***,~	0.66 ****	0.72 **,~
PTEN-IGF1RKO	0.88	0.98 ~~~~	0.98 ~~~~	0.87 ~~~~	0.86 ~~~~	0.88 ~~~~
Triple KO	0.95	0.92 ~~	0.89 ~~~~	0.81 **,~~~~	0.78 ***,~~~	0.79 **,~~~

Fig. 4. Hepatic glucose output and systemic glucose tolerance are under the control of the IGF1R/PTEN signaling axis in hepatocytes. (A) GTTs performed in overnight fasted CTRL (n = 31), PTENKO (n = 14), PTEN-IRKO (n = 9), PTEN-IGF1RKO (n = 16), and triple-KO (n = 13) mice. The AUC was calculated for the 120 min after glucose injection. (B) Glycemia (fed/fasted state: CTRL n = 8/n = 12, PTENKO n = 10/n = 12, PTEN-IRKO n = 8/n = 12, PTEN-IGF1RKO n = 7/n = 12, and triple-KO n = 9/n = 12), insulinemia (fed/fasted state: CTRL n = 5/n = 5, PTENKO n = 5/n = 5, PTEN-IRKO n = 4/n = 5, PTEN-IGF1RKO n = 5/n = 6, and triple-KO n = 4/n = 5), and glucagon (fed/fasted state: CTRL n = 4/n = 5, PTENKO n = 3/n = 5, PTEN-IRKO n = 3/n = 4, PTEN-IGF1RKO n = 4/n = 4, and triple-KO n = 4/n = 4) were measured before sacrifice. (C) PTTs performed in overnight fasted CTRL (n = 34), PTENKO (n = 11), PTEN-IRKO (n = 9), PTEN-IGF1RKO (n = 16), and triple-KO (n = 19) mice. For PTTs, the AUC was calculated for the first 30 min after pyruvate injection. (D) Hepatic glycogen content under fed conditions (CTRL n = 8–11, PTENKO n = 7–14, PTEN-IRKO n = 9, PTEN-IGF1RKO n = 10, and triple-KO n = 12) in absolute value (right panel) and normalized to liver weights (left panel). (E) Relative mRNA expression of key

patients). Gene Set Enrichment Analysis (GSEA) was then performed to determine the enriched molecular signatures in the high *IGF1R*-expressing group (Molecular Signatures Database [MSigDB] hallmark gene set collection). Interestingly, the only three hallmarks found to be significantly enriched in low-*IGF1R* tumors (FDR q value <0.05) were bile acid metabolism, fatty acid metabolism, and peroxisome (Fig. S9). These correlations are consistent with the effect of IGF1R knockout on lipid oxidation in our mouse model. Notably, no significant enrichment was found when GSEA was performed in biopsies expressing high INSR *vs.* low INSR. At the protein level, data from the Human Protein Atlas indicated that IR is consistently present in patient HCC samples (n = 7, all samples show intermediate intensity staining), whereas the IGF1R detection showed high variability between samples (n = 11, ranging from undetectable to high levels of IGF1R) (Fig. S10). Altogether, these results demonstrate that IR is present with some consistency in HCC samples and its RNA/protein levels are unlikely to be a good biomarker candidate for patient prognosis. On the contrary, IGF1R has potential for biomarker use, but more extensive analyses are required to consider this receptor in clinical diagnostics.

As PTENKO mice develop hepatic tumors with aging in the context of MASLD,[12,13] we assessed cancer development in all mutant groups at 12 months of age. Consistent with the liver weights at 4 months (Fig. 1), liver weights at 12 months were significantly increased in PTENKO and PTEN-IGF1RKO mice compared with CTRL mice (Fig. S11). Additional deletion of IR in PTENKO livers reduced liver weight, but it remained slightly elevated compared with that of CTRL mice. Genetic ablation of both receptors was required to completely prevent hepatomegaly, as observed in triple-KO mice (Fig. S11). Liver dissection revealed the presence of tumor nodules in all mutant mice, but a nodular liver structure was more prominent in PTENKO and PTEN-IGF1RKO mice than in the other mutant mice (Fig. 5D).

Histological analysis of the liver/tumor sections was performed by a trained pathologist, and tumoral lesions were classified according to Thoolen *et al.*[31] (Fig. S12A). The assessment revealed that in 12-month-old PTENKO mice, the liver appeared nodular and exhibited steatohepatitis features (Figs S12A and S13Ba and b). All the mice in this group developed hepatocellular nodules composed of well-differentiated hepatocytes arranged in regular and thin liver plates. They had a clear, ballooned, or steatotic cytoplasm and corresponded to hepatocellular adenomas (HCAs). A few small biliary lesions with inflammatory cells (cholangiomas) were detected as well. In PTEN-IRKO mice, the liver was much less nodular, and there was no obvious steatohepatitis (Fig. S12A and Ca–f). A few steatotic HCAs were present, smaller than those in PTENKO mice, showing most of the time a combination with biliary elements, giving rise to mixed hepatobiliary tumors. Cholangiomas were also present. PTEN-IGF1RKO mice had a nodular liver and exhibited steatohepatitis. The nodules they developed either were HCAs, as seen in PTENKO mice, or were larger and corresponded to fully developed HCCs, with thick trabeculae, atypical cells, and necrosis (Fig. S12A and Da and b). Some mixed hepatobiliary nodules and cholangiomas were also present. Finally, triple-KO mice did not display nodular parenchyma or steatohepatitis. Nodules corresponded to HCA, HCC, or mixed hepatobiliary nodules (Fig. S12A and Ea–c). A few cholangiomas were found. Additional CK19 was performed to further characterize the biliary lesions seen in the mutants (Fig. S12). Biliary lesions correspond to hyperplasia in some portal tracts (not illustrated), cholangioma (very frequently observed), and hepatocholangioma (not seen in PTENKO mice) without fully developed cholangiocarcinoma or hepatocholangiocarcinoma (Fig. S12). Of note, there was no CK19 expression in HCA or HCC. In general, the biliary lesions observed in our models were all benign looking, well-defined with regular contours, and without formally identified malignancy or overt fully developed cholangiocarcinoma. Nevertheless, this observation is interesting as previous studies indicate an association between intrahepatic cholangiocarcinoma and MASLD/MASH incidence.[32–34] Therefore, these numerous cholangiomas and, in rarer cases, hepatocholangiomas might be linked to biliary carcinogenesis in these MASLD-associated models.

Liver volume, tumor number, and tumor volume quantification were performed using *in vivo* CT scan imaging (Fig. 5E). The quantified liver volume showed the same profile as compared with the liver weight of the mutant mice (Fig. 5F). The liver volumes of PTENKO and PTEN-IGF1RKO mice were increased compared with those of CTRL livers. However, the liver volumes of PTEN-IRKO and triple-KO mice were similar to those of CTRL mice (Fig. 5F). In addition, the inhibition of IR and/or IGF1R signaling in PTENKO mice also tended to reduce tumor number and total tumor volume, whereas the average tumor volume was not statistically different between the mutant mice (Fig. 5G). However, PTEN-IGF1RKO mice displayed a clear tendency to have the highest average tumor volume. These data confirm that the inhibition of IR signaling in PTEN-deficient hepatocytes is necessary to prevent the development of hepatomegaly and the progression of MASLD. Furthermore, inhibition of IR and/or IGF1R reduced tumor formation in PTEN-deficient livers but did not completely prevent the appearance of cancerous foci.

To further characterize the tumors in the different groups, an assessment of different markers was performed via quantitative reverse transcription PCR (RT-qPCR). No changes in expression were observed with the differentiation marker hepatocyte nuclear factor 4a (*Hnf4a*) (Fig. S13). Interestingly, tumors that developed in PTEN-IRKO and triple-KO livers presented a lower average expression of vascular endothelial growth factor a (*Vegfa*, a marker of angiogenesis) and glypican 3 (*Gpc3*, a marker of malignancy), compared with PTENKO and PTEN-IGF1RKO tumors. Nevertheless, the highly variable number of

enzymes regulating glycolysis in 4-month-old CTRL, PTENKO, PTEN-IRKO, PTEN-IGF1RKO, and triple-KO mice under fasted conditions (left panel; CTRL n = 10, PTENKO n = 10, PTEN-IRKO n = 8, PTEN-IGF1RKO n = 10, and triple-KO n = 10) and fed conditions (right panel; CTRL n = 12, PTENKO n = 10, PTEN-IRKO n = 10, PTEN-IGF1RKO n = 10, and triple-KO n = 12). Values are represented as mean ± SD. One-way ANOVA was performed. Values were considered significant when compared with CTRL (*) and PTENKO (~): */~ $p \le 0.05$, **/~~ $p \le 0.01$, ***/~~~ $p \le 0.001$, or ****/~~~~ $p \le 0.0001$. GTT, glucose tolerance test; IGF1R, insulin-like growth factor 1 receptor; PTEN, phosphatase and tensin homolog; PTT, pyruvate tolerance test.

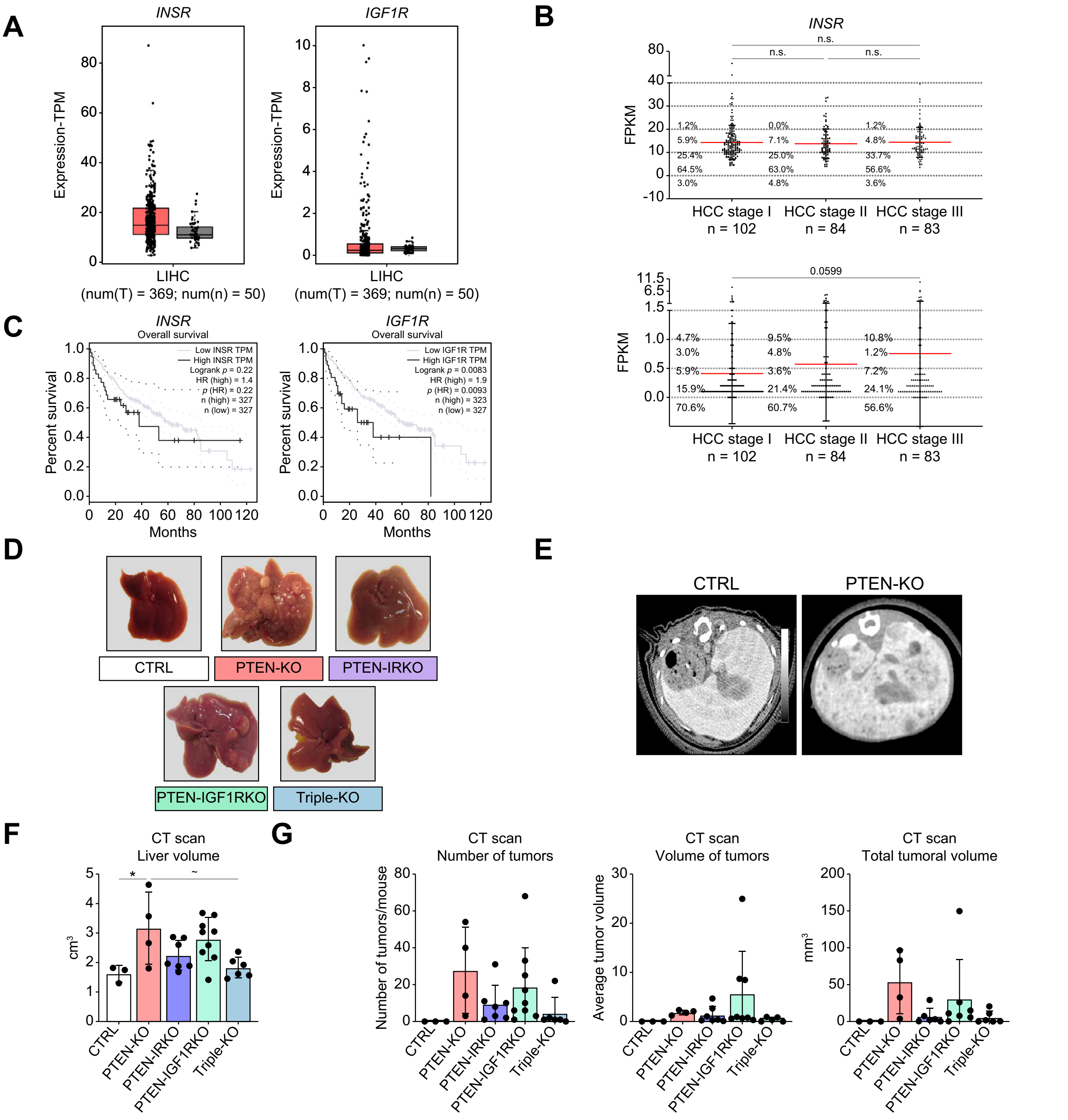

Fig. 5. Hepatic IR and IGF1R deletion trigger the formation of different liver cancers induced with PTEN deletion. (A) Relative mRNA expression of the *INSR* and *IGF1R* genes in nontumoral liver (gray box, n = 50) *vs*. HCC (black box, n = 369) samples from patients in the TCGA cohort (liver hepatocellular carcinoma subgroup—LIHC).[30] (B) Relative mRNA expression of the *INSR* and *IGF1R* genes in HCC samples from patients in the TCGA cohort, segregated by the stage of the tumor. (C) Kaplan–Meier survival representation of the LIHC patients in the TCGA cohort regarding the level of *INSR* or *IGF1R* gene expression (10% of the patients with the highest gene expression *vs*. 90% of the rest of the patients). Graphical representation and statistical assessment for (A) and (C) were done through the Gepia2 cancer database (http://gepia2.cancer-pku.cn/#index). (D) Representative liver macroscopic morphology. (E) CT scan micrographs, (F) liver volume determined by CT scan, and (G) hepatic tumor number, average tumor volume, and total tumoral volume in 12-month-old CTRL (n = 3), PTENKO (n = 4), PTEN-IRKO (n = 7), PTEN-IGF1RKO (n = 7–9), and triple-KO (n = 5–6) mice under fed conditions. Values are represented as mean ± SD. One-way ANOVA was performed. Values were considered significant when compared with CTRL (*) and PTENKO (~): */~ $p \le 0.05$, **/~~ $p \le 0.01$, ***/~~~ $p \le 0.001$, or ****/~~~~ $p \le 0.0001$. CT, computed tomography; HCC, hepatocellular carcinoma; IGF1R, insulin-like growth factor 1 receptor; IR, insulin receptor; PTEN, phosphatase and tensin homolog.

tumors per group did not allow statistical assessment of these data. Finally, measurement of interleukin 6 (*Il6*) and interleukin 1b (*Il1b*) revealed increased expression of these cytokines in PTEN-IRKO and PTEN-IGF1KO tumors, in comparison with PTENKO tumors (Fig. S13). Again, these data were not statistically significant because of the variable distribution of available tumoral samples per group.

Interestingly, all mutant livers at 12 months of age presented some bile duct malformations, suggesting that PTEN depletion induces specific processes leading to alterations in the biliary tract. Unexpectedly, the absence of hepatic IGF1R signaling promoted the malignant transformation of some nodules. Indeed, PTEN-IGF1RKO and triple-KO mice had clear HCC, composed of malignant hepatocytes, unlike PTENKO and PTEN-IRKO mice. Therefore, IR and IGF1R signalizations have distinct roles in PTEN deficiency-induced hepatocarcinogenesis.

Discussion

The IR and IGF1R are closely related receptors sharing a high degree of structural similarity. Upon stimulation by their ligands, both receptors transduce signaling through the PI3K/AKT and MAPK pathways, albeit with different terminal outputs. Genetic studies investigating mouse phenotypes associated with the single constitutive deletion of each receptor have suggested that the IR is mainly involved in metabolic signaling, whereas the IGF1R triggers principally mitogenic signaling.

Our data indicate that in the liver, signaling through the IR and IGF1R controls distinct processes that regulate hepatic glucose and lipid homeostasis, which are disrupted by PTEN deficiency. Loss of PTEN expression/activity in hepatocytes rapidly induces an aberrant accumulation of lipids in the hepatocytes.[8,14,35] Herein, we found that signaling through the IR is critical to activate DNL and induce steatosis in the absence of PTEN. Indeed, PTEN-IRKO mice showed reduced levels of hepatic DNL and steatosis compared with PTENKO mice. In agreement, IR knockdown in *ob/ob* mice decreases the expression of DNL genes and ameliorates hepatic steatosis.[36] Deletion of the IR in PTEN-deficient hepatocytes did not completely prevent the accumulation of lipid droplets. Our biochemical analyses of the lipid content in the liver of these mice indicated that these remaining lipid droplets could accumulate cholesteryl esters, consistent with the observed upregulation of key enzymes involved in cholesterol metabolism. Biddinger *et al.*[37] previously reported that hepatic insulin resistance induced by deletion of the IR in the liver of mice under dietary challenge was associated with increased cholesterol metabolism and the presence of cholesterol gallstones.[37] Furthermore, in our mutant mice, the additional deletion of PTEN in hepatocytes mimics a state of hepatic insulin hypersensitivity, suggesting that AKT-independent IR signaling inhibits cholesterol metabolism.

In addition to the presence of steatosis in PTENKO mice, these animals are hypoinsulinemic and have impaired HGO, as well as increased insulin sensitivity and glucose uptake in skeletal muscle.[14] In contrast, LIRKO mice (hepatocyte-specific deletion of IR) develop hyperglycemia and hyperinsulinemia associated with increased hepatic glucose production, but not steatosis.[17] HGO was still partially inhibited in PTEN-IRKO mice, indicating that this mechanism is not strictly dependent on IR signaling upstream of PTEN. In contrast, the absence of IGF1R in hepatocytes reverses the inhibition of HGO associated with PTEN deficiency, which appears to abolish the benefit of PTEN deletion in improving glucose tolerance. However, how hepatic IGF1R signaling modulates glucose metabolism in the liver remains unclear. Nevertheless, our findings corroborate previously published reports demonstrating that in the absence of IR, IGF-1 can mimic the function of insulin and inhibit hepatic gluconeogenesis through its receptor IGF1R.[38]

Our study further demonstrated that hepatic IR and IGF1R signaling, in concert with PTEN, differentially affect adipose tissue metabolic homeostasis. The molecular mechanisms by which impaired hepatic IR/IGF1R/PTEN activity affects peripheral organs are poorly characterized. Accumulating evidence suggests that the liver can release circulating endocrine factors called hepatokines,[16] which play key roles in metabolic homeostasis and energy expenditure under physiological and pathological conditions. Among them, we have previously identified a myriad of hepatokines (*e.g.* ANGPTL4, FGF-21, FETUA, FETUB, LECT2, IGFBP1, and IGFBP2), deregulated in the livers of PTENKO mice and involved in muscle insulin sensitivity, glucose uptake, adipogenesis, energy expenditure, and browning of WAT.[14,16] Together, these hepatokines may contribute to the liver-to-peripheral organ crosstalk and the metabolic phenotype observed in our mouse models. The hepatic expression of *Ahsg* (FETUA), *Fetub*, and *Igfbp2* was decreased in PTENKO mice compared with CTRL mice, and it remained decreased in the PTEN-IGF1RKO group (Fig. S14). The mRNA levels of these hepatokines seem dependent on the presence of IR, as in both PTEN-IRKO and triple-KO livers, the expression was rescued to the CTRL level. A similar pattern of expression was observed for *Lect2*, although the differences were not significant. Intriguingly, *Igfbp1* expression presented a tendency to decrease in the PTENKO group compared with that in the CTRL group, whereas the PTEN-IGF1RKO group presented a significant increase in expression (Fig. S14). *Fgf21* expression presented a tendency to increase in the PTENKO group, and a further increase was observed in the PTEN-IGF1RKO and triple-KO groups (Fig. S14). This hepatokine is known to exert beneficial effects on muscle insulin sensitivity and glucose uptake in pathological states. Similarly, *Angptl4* presented a tendency to increase in PTENKO livers, compared with CTRL livers; however, only the triple-KO group presented a significant increase in the expression of this hepatokine.

Finally, a major finding of this study is that the triple knockout of IR, IGF1R, and PTEN in hepatocytes induces only minor metabolic changes in young adult mice. Indeed, triple-KO mice have a completely normal liver, without any steatosis and with a gene expression profile comparable to that of control mice. How these mice cope with the lack of these three master metabolic regulators under stress conditions remains to be investigated; however, under normal breeding conditions, triple-KO mice surprisingly do not display any abnormal metabolic phenotype. This observation suggests that PTEN deficiency exerts an important overriding control over the effects of IR and IGF1R deletion on hepatic metabolism.

PTEN deficiency in hepatocytes was also shown to promote the development of hepatic nodular lesions in 1-year-old mice, which can progress to HCC. This genetic model of HCC mimics the development of HCC in patients with steatohepatitis, which has a poor prognosis.[12] MASLD and diabetes, as well as abnormal insulin and IGF-1 signaling, have been identified as important risk factors and contributors to HCC development.[39–41] This study

provides evidence that IR and IGF1R are components in the process of hepatocarcinogenesis induced by hepatic PTEN deletion. We demonstrate that the deletion of IR and/or IGF1R does not prevent the formation of liver tumors in PTENKO mice at 12 months of age. Nevertheless, the number of tumors and the average tumor volume were reduced in PTEN-IRKO and triple-KO mice, as compared with PTENKO mice. IR signaling induced the formation of nodules with severe steatosis in PTENKO and PTEN-IGF1RKO mice. In addition, both mutant mice displayed HCA. In contrast, steatohepatitis was greatly reduced or even absent in tumor nodules of PTEN-IRKO and triple-KO mice. However, the tumoral lesions in both mutant mice were mixed hepatobiliary tumors of unknown malignant potential, characterized by distinct bile duct malformations lined by liver-cell nests or plates. Some PTEN-IRKO mice also developed pure cholangioma. It is noteworthy that the Cre-recombinase under the albumin promoter (Alb-Cre) used to excise flox sites in PTEN, IR, and/or IGF1R in this study is active in cholangiocytes during development, and therefore, all the mutants lack the expression of these three genes in these cells. Of note, PTEN, IGF1R, and IR are all expressed in hepatocytes and cholangiocytes (Fig. S15A and B). Therefore, the cholangiomas we observed could originate from cholangiocytes lacking PTEN/IR/IGF1R expression in our models. Nevertheless, the origin of these cholangiomas could also be transformed hepatocytes, as previous studies have reported hepatocyte-driven ICC development. For example, this has been observed *in vivo* in the AKT-NICD model of liver carcinogenesis.[42,43]

Unexpectedly, the inhibition of IGF1R signaling induced malignant transformation of tumor foci in PTEN-IGF1RKO and triple-KO mice. These data suggest that neither IR nor IGF1R are critical for the oncogenic transformation in PTENKO livers. Rather, PTEN deletion in combination with the IGF1R silencing may trigger mitogenic processes that ultimately lead to tumor malignancy. Notably, other studies have shown that the inhibition of IGF1R signaling does not prevent the induction of hepatocarcinogenesis in mice.[44,45] Moreover, in some cases, IGF1R inhibition and downregulation still induced IGF1R downstream signaling despite an apparent inhibition of the receptor phosphorylation, which is reviewed in detail elsewhere.[46] The ineffectiveness of a single IGF1R anticancer therapy is often explained by the increased formation of IGF/IR heteroreceptors[25,26] and by the increased abundance of the oncogenic IR-A isoform in tumoral tissues,[47] which efficiently activates the PI3K/AKT and RAS/RAF/MAPK signaling cascades. IR-A is generated by alternative splicing and lacks exon 11 compared with IR-B, which accounts for the relevant functional differences between the two IR isoforms.[47] IGF-2 strongly activates IR-A, thereby promoting proliferation, migration, and inhibition of apoptosis. Interestingly, tumors that overexpress IR-A and IGF-2, were resistant to IGF1R anticancer therapy alone.[47,48] To our surprise, IR-A expression levels were significantly lower in PTEN-IGF1RKO tumors than in PTENKO tumors, whereas IR-B expression was unchanged (Fig. S16). This, in turn, resulted in lower IR-A/IR-B ratios in PTEN-IGF1RKO tumors than in PTENKO tumors (Fig. S16). Therefore, an increased IR-A/IR-B ratio can be excluded as a possible favored mechanism of malignancy in these mouse groups. Taken together, these findings suggest that PTEN may not only counteract the proliferative and mitogenic properties of IR and IGF1R signaling. Instead, PTEN may also regulate the expression of components within the insulin/IGF system. This would add a new layer to the tumor-suppressive activities of PTEN.

Affiliations

[1]*Department of Cell Physiology and Metabolism, Faculty of Medicine, University of Geneva, Geneva, Switzerland;* [2]*Animal Sciences Department, Faculty of Medicine, University of Geneva, CH-1211 Geneva, Switzerland;* [3]*Department of Radiology, Faculty of Medicine, University of Geneva, Geneva, Switzerland;* [4]*Service of Clinical Pathology, Institute of Pathology, Lausanne University Hospital, University of Lausanne, Lausanne, Switzerland;* [5]*Department of Genetic Medicine and Development, Faculty of Medicine, University of Geneva, CH-1211 Geneva, Switzerland*

Abbreviations

ALT, alanine transaminase; AST, aspartate transaminase; BAT, brown adipose tissue; CT, computed tomography; DNL, *de novo* lipogenesis; GEO, Gene Expression Omnibus; GSEA, Gene Set Enrichment Analysis; GTT, glucose tolerance test; HCA, hepatocellular adenoma; HCC, hepatocellular carcinoma; HGO, hepatic glucose output; IGF1R, insulin-like growth factor 1 receptor; IR, insulin receptor; LIHC, liver hepatocellular carcinoma; MASH, metabolic dysfunction-associated steatohepatitis; MASLD, metabolic dysfunction-associated steatotic liver disease; MSigDB, Molecular Signatures Database; PTEN, phosphatase and tensin homolog; PTT, pyruvate tolerance test; RER, respiratory exchange ratio; TG, triglyceride; WAT, white adipose tissue.

Financial support

This work was supported by the Bo & Kerstin Hjelt Foundation and the Swiss National Science Foundation (grant no. 310030_172862), both attributed to MiF.

Conflicts of interest

The authors have no conflict of interest to declare.

Please refer to the accompanying ICMJE disclosure forms for further details.

Authors' contributions

Conceptualization: MiF. Data curation: MG, NC, CyS, DP, J-LP, FB, CM, MaF, A-SA, MCdS, ED, LV, XM, ChS, SN, MiF. Investigation: MG, NC, CyS, DP, CM, MaF, ChS, MiF. Funding acquisition: MiF. Project administration: MiF. Resources: MiF. Supervision: MiF. Visualization: NC, MG. Writing: MG, NC, DP, MiF. Have read and agreed to the published version of the manuscript: all authors.

Data availability statement

Data presented in this study are openly available in the Yareta data repository at the following link: 10.26037/yareta:zwyyaxdj3zc3hfo3hykxvwmt6q.111

Acknowledgements

The authors are deeply saddened by the untimely death of Michelangelo Foti, our most valued colleague and dear friend. The authors thank Prof. Pierre Maechler for the support provided during the completion of this study, following Prof. Michelangelo Foti's death. We warmly thank Prof. A. Efstratiadis (New York, NY) for providing us with $Insr^{flox}$ ($Insr^{fx/fx}$) and $Igf1r^{flox}$ ($Igf1r^{fx/fx}$) mice.

Declaration of Generative AI and AI-assisted technologies in the writing process

During the preparation of this work, the authors used ChatGPT to improve language and readability. After using this tool/service, the authors reviewed and edited the content as needed and take full responsibility for the content of the publication.

Supplementary data

Supplementary data to this article can be found online at https://doi.org/10.1016/j.jhepr.2024.101305.

References

Author names in bold designate shared co-first authorship

[1] Chan WK, Chuah KH, Rajaram RB, et al. Metabolic dysfunction-associated steatotic liver disease (MASLD): a state-of-the-art review. J Obes Metab Syndr 2023;32:197–213.
[2] Ando Y, Jou JH. Nonalcoholic fatty liver disease and recent guideline updates. Clin Liver Dis 2021;17:23–28.
[3] Kitade H, Chen G, Ni Y, et al. Nonalcoholic fatty liver disease and insulin resistance: new insights and potential new treatments. Nutrients 2017;9:387.
[4] Wang S, Friedman SL. Found in translation—fibrosis in metabolic dysfunction-associated steatohepatitis (MASH). Sci Transl Med 2023;15:eadi0759.
[5] Pinyopornpanish K, Khoudari G, Saleh MA, et al. Hepatocellular carcinoma in nonalcoholic fatty liver disease with or without cirrhosis: a population-based study. BMC Gastroenterol 2021;21:394.
[6] Zimmermann C, Cederroth CR, Bourgoin L, et al. Prevention of diabetes in *db/db* mice by dietary soy is independent of isoflavone levels. Endocrinology 2012;153:5200–5211.
[7] Courty E, Besseiche A, Do TTH, et al. Adaptive β-cell neogenesis in the adult mouse in response to glucocorticoid-induced insulin resistance. Diabetes 2019;68:95–108.
[8] Stiles B, Wang Y, Stahl A, et al. Liver-specific deletion of negative regulator Pten results in fatty liver and insulin hypersensitivity [corrected]. Proc Natl Acad Sci U S A 2004;101:2082–2087.
[9] Dayyani F, Parikh NU, Varkaris AS, et al. Combined inhibition of IGF-1R/IR and Src family kinases enhances antitumor effects in prostate cancer by decreasing activated survival pathways. PLoS One 2012;7:e51189.
[10] Weng LP, Smith WM, Brown JL, et al. PTEN inhibits insulin-stimulated MEK/MAPK activation and cell growth by blocking IRS-1 phosphorylation and IRS-1/Grb-2/Sos complex formation in a breast cancer model. Hum Mol Genet 2001;10:605–616.
[11] Wijesekara N, Konrad D, Eweida M, et al. Muscle-specific Pten deletion protects against insulin resistance and diabetes. Mol Cel Biol 2005;25:1135–1145.
[12] Horie Y, Suzuki A, Kataoka E, et al. Hepatocyte-specific Pten deficiency results in steatohepatitis and hepatocellular carcinomas. J Clin Invest 2004;113:1774–1783.
[13] **Peyrou M**, **Bourgoin L**, **Foti M**. PTEN in liver diseases and cancer. World J Gastroenterol 2010;16:4627–4633.
[14] Peyrou M, Bourgoin L, Poher AL, et al. Hepatic PTEN deficiency improves muscle insulin sensitivity and decreases adiposity in mice. J Hepatol 2015l;62:421–429.
[15] Wong JT, Kim PT, Peacock JW, et al. Pten (phosphatase and tensin homologue gene) haploinsufficiency promotes insulin hypersensitivity. Diabetologia 2007;50:395–403.
[16] Berthou F, Sobolewski C, Abegg D, et al. Hepatic PTEN signaling regulates systemic metabolic homeostasis through hepatokines-mediated liver-to-peripheral organs crosstalk. Int J Mol Sci 2022;23:3959.
[17] Michael MD, Kulkarni RN, Postic C, et al. Loss of insulin signaling in hepatocytes leads to severe insulin resistance and progressive hepatic dysfunction. Mol Cel 2000;6:87–97.
[18] El Ouaamari A, Kawamori D, Dirice E, et al. Liver-derived systemic factors drive beta cell hyperplasia in insulin-resistant states. Cell Rep 2013;3:401–410.
[19] Pérez-Matute P, López IP, Íñiguez M, et al. IGF1R is a mediator of sex-specific metabolism in mice: effects of age and high-fat diet. Front Endocrinol 2022;13:1033208.
[20] Desbois-Mouthon C, Wendum D, Cadoret A, et al. Hepatocyte proliferation during liver regeneration is impaired in mice with liver-specific IGF-1R knockout. FASEB J 2006;20:773–775.
[21] Cadoret A, Rey C, Wendum D, et al. IGF-1R contributes to stress-induced hepatocellular damage in experimental cholestasis. Am J Pathol 2009;175:627–635.
[22] Aleem E, Nehrbass D, Klimek F, et al. Upregulation of the insulin receptor and type I insulin-like growth factor receptor are early events in hepatocarcinogenesis. Toxicol Pathol 2011;39:524–543.
[23] McGlynn KA, Petrick JL, El-Serag HB. Epidemiology of hepatocellular carcinoma. Hepatology 2021;73(Suppl. 1):4–13. Suppl. 1.
[24] **Streba LA**, **Vere CC**, **Rogoveanu I**, et al. Nonalcoholic fatty liver disease, metabolic risk factors, and hepatocellular carcinoma: an open question. World J Gastroenterol 2015;21:4103–4110.
[25] Massoner P, Ladurner-Rennau M, Eder IE, et al. Insulin-like growth factors and insulin control a multifunctional signalling network of significant importance in cancer. Br J Cancer 2010;103:1479–1484.
[26] Singh P, Alex JM, Bast F. Insulin receptor (IR) and insulin-like growth factor receptor 1 (IGF-1R) signaling systems: novel treatment strategies for cancer. Med Oncol 2014;31:805.
[27] Tognon CE, Sorensen PH. Targeting the insulin-like growth factor 1 receptor (IGF1R) signaling pathway for cancer therapy. Expert Opin Ther Targets 2012;16:33–48.
[28] **Ngo MT**, **Jeng HY**, Kuo YC, et al. The role of IGF/IGF-1R signaling in hepatocellular carcinomas: stemness-related properties and drug resistance. Int J Mol Sci 2021;22:1931.
[29] **D'Alessandro R**, **Refolo MG**, Lippolis C, et al. Strong enhancement by IGF1-R antagonists of hepatocellular carcinoma cell migration inhibition by Sorafenib and/or vitamin K1. Cel Oncol (Dordr) 2018;41:283–296.
[30] Tomczak K, Czerwińska P, Wiznerowicz M. The Cancer Genome Atlas (TCGA): an immeasurable source of knowledge. Contemp Oncol (Pozn) 2015;19:A68–A77.
[31] Thoolen B, Maronpot RR, Harada T, et al. Proliferative and nonproliferative lesions of the rat and mouse hepatobiliary system. Toxicol Pathol 2010;38(7 Suppl.):5s–81s.
[32] Izquierdo-Sanchez L, Lamarca A, La Casta A, et al. Cholangiocarcinoma landscape in Europe: diagnostic, prognostic and therapeutic insights from the ENSCCA Registry. J Hepatol 2022;76:1109–1121.
[33] Pascale A, Rosmorduc O, Duclos-Vallée JC. New epidemiologic trends in cholangiocarcinoma. Clin Res Hepatol Gastroenterol 2023;47:102223.
[34] Petrick JL, Yang B, Altekruse SF, et al. Risk factors for intrahepatic and extrahepatic cholangiocarcinoma in the United States: a population-based study in SEER-Medicare. PLoS One 2017;12:e0186643.
[35] Clement S, Peyrou M, Sanchez-Pareja A, et al. Down-regulation of phosphatase and tensin homolog by hepatitis C virus core 3a in hepatocytes triggers the formation of large lipid droplets. Hepatology 2011;54:38–49.
[36] **Haas JT**, **Miao J**, Chanda D, et al. Hepatic insulin signaling is required for obesity-dependent expression of SREBP-1c mRNA but not for feeding-dependent expression. Cell Metab 2012;15:873–884.
[37] Biddinger SB, Haas JT, Yu BB, et al. Hepatic insulin resistance directly promotes formation of cholesterol gallstones. Nat Med 2008;14:778–782.
[38] Di Cola G, Cool MH, Accili D. Hypoglycemic effect of insulin-like growth factor-1 in mice lacking insulin receptors. J Clin Invest 1997;99:2538–2544.
[39] Orgel E, Mittelman SD. The links between insulin resistance, diabetes, and cancer. Curr Diab Rep 2013;13:213–222.
[40] Weroha SJ, Haluska P. The insulin-like growth factor system in cancer. Endocrinol Metab Clin North Am 2012;41:335–350 [vi].
[41] LeRoith D, Roberts Jr CT. The insulin-like growth factor system and cancer. Cancer Lett 2003;195:127–137.
[42] **Fan B**, **Malato Y**, **Calvisi DF**, et al. Cholangiocarcinomas can originate /from hepatocytes in mice. J Clin Invest 2012;122:2911–2915.
[43] Hu S, Molina L, Tao J, et al. NOTCH-YAP1/TEAD-DNMT1 axis drives hepatocyte reprogramming into intrahepatic cholangiocarcinoma. Gastroenterology 2022;163:449–465.
[44] Cadoret A, Desbois-Mouthon C, Wendum D, et al. c-myc-induced hepatocarcinogenesis in the absence of IGF-I receptor. Int J Cancer 2005;114:668–672.
[45] Chen YW, Boyartchuk V, Lewis BC. Differential roles of insulin-like growth factor receptor- and insulin receptor-mediated signaling in the phenotypes of hepatocellular carcinoma cells. Neoplasia 2009;11:835–845.
[46] Girnita L, Worrall C, Takahashi S, et al. Something old, something new and something borrowed: emerging paradigm of insulin-like growth factor type 1 receptor (IGF-1R) signaling regulation. Cell Mol Life Sci 2014;71:2403–2427.
[47] Belfiore A, Malaguarnera R. Insulin receptor and cancer. Endocr Relat Cancer 2011;18:R125–R147.
[48] Enguita-German M, Fortes P. Targeting the insulin-like growth factor pathway in hepatocellular carcinoma. World J Hepatol 2014;6:716–737.

Keywords: Hepatic metabolism; Hepatocarcinogenesis; Insulin receptor; IGF-1 receptor; Steatosis.
Received 14 March 2024; received in revised form 5 December 2024; accepted 10 December 2024; Available online 19 December 2024

Journal of Hepatology, Volume *7*

Supplemental information

Hepatic IR and IGF1R signaling govern distinct metabolic and carcinogenic processes upon PTEN deficiency in the liver

Monika Gjorgjieva, Nicolas Calo, Cyril Sobolewski, Dorothea Portius, Jean-Luc Pitetti, Flavien Berthou, Anne-Sophie Ay, Marion Peyrou, Lucie Bourgoin, Christine Maeder, Margot Fournier, Marta Correia de Sousa, Etienne Delangre, Laurent Vinet, Xavier Montet, Christine Sempoux, Serge Nef, and Michelangelo Foti

Hepatic IR and IGF1R signaling govern distinct metabolic and carcinogenic processes upon PTEN deficiency in the liver

Monika Gjorgjieva, Nicolas Calo, Cyril Sobolewski, Dorothea Portius, Jean-Luc Pitetti, Flavien Berthou, Anne-Sophie Ay, Marion Peyrou, Lucie Bourgoin, Christine Maeder, Margot Fournier, Marta Correia de Sousa, Etienne Delangre, Laurent Vinet, Xavier Montet, Christine Sempoux, Serge Nef, Michelangelo Foti

Table of contents

Supplementary figures………………………………………………………………………....2

Table S1………………………………………………………………………………….....24

Supplementary materials and methods……………………………………………….…25

Supplementary references……………………………………………………………...30

Supplementary figures

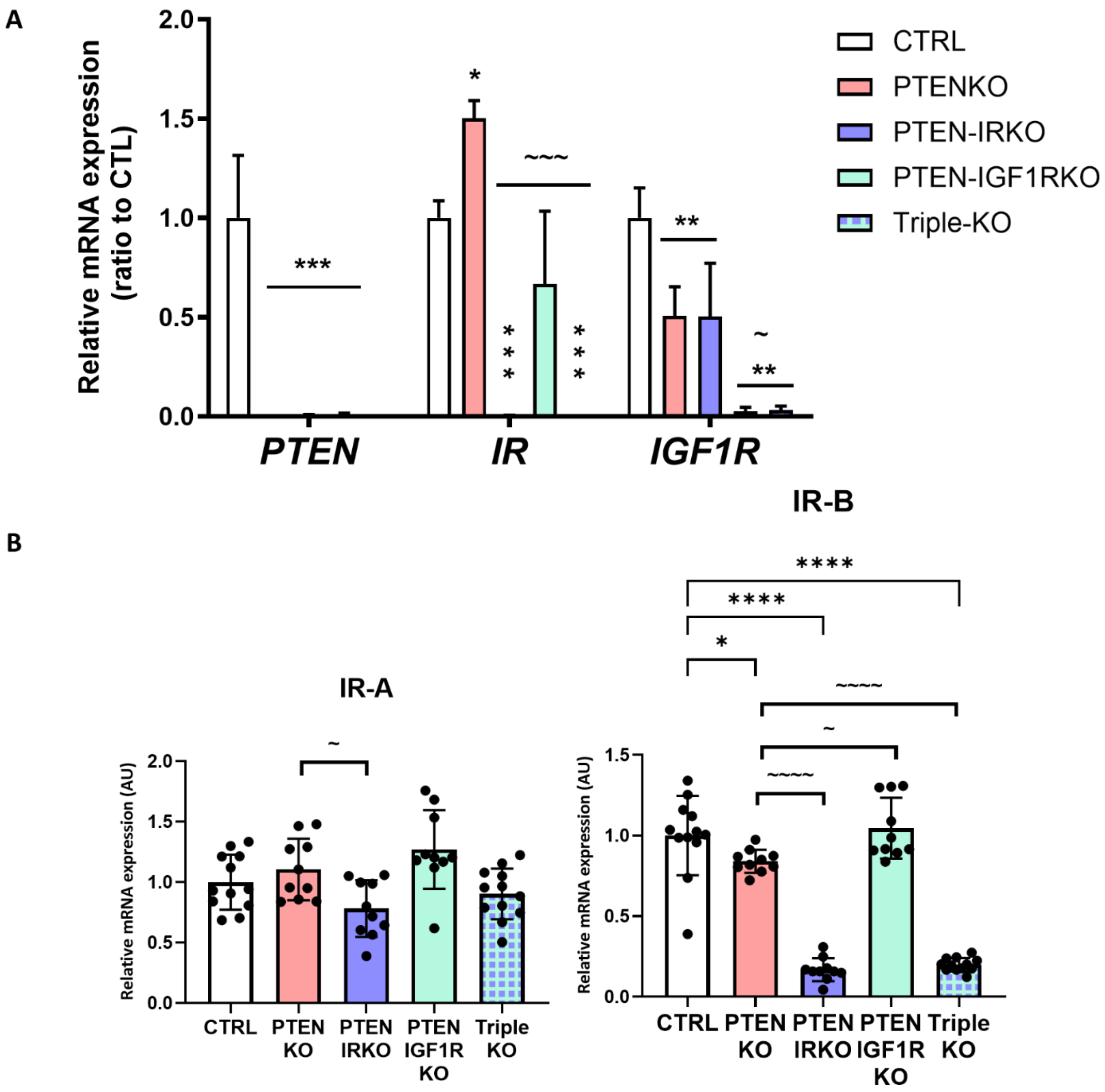

Fig. S1: Validation of hepatocyte-specific deletions of IR, IGF1R and PTEN in the different mutants.

(A) Specific genomic ablations of *Pten*, *Insr* and *Igf1r* were confirmed by RT-qPCR on isolated primary mouse hepatocytes. Values are mean ± SD of 3 mice per group. Primary mouse hepatocytes were isolated and seeded as previously described in Horie et al. [1]. After 24h, supernatant, protein and RNA were collected and flash frozen at -80°C (one-way ANOVA used for statistical analysis).

(B) Insulin receptor (IR) isoform A and B mRNA expression was investigated under fed conditions in the livers of CTRL, PTENKO, PTEN-IRKO, PTEN-IGF1RKO and triple-KO mice at 4 months of age *via* RT-qPCR. Values are mean +/- SD of 10-12 mice per group (2-way ANOVA used for statistical analysis).

Values were considered significant when compared to CTRL (*) or to PTENKO (~):
*/~ p≤ 0.05, **/~~ p≤ 0.01,***/~~~ p≤ 0.001 or ****/~~~~ p≤ 0.0001.

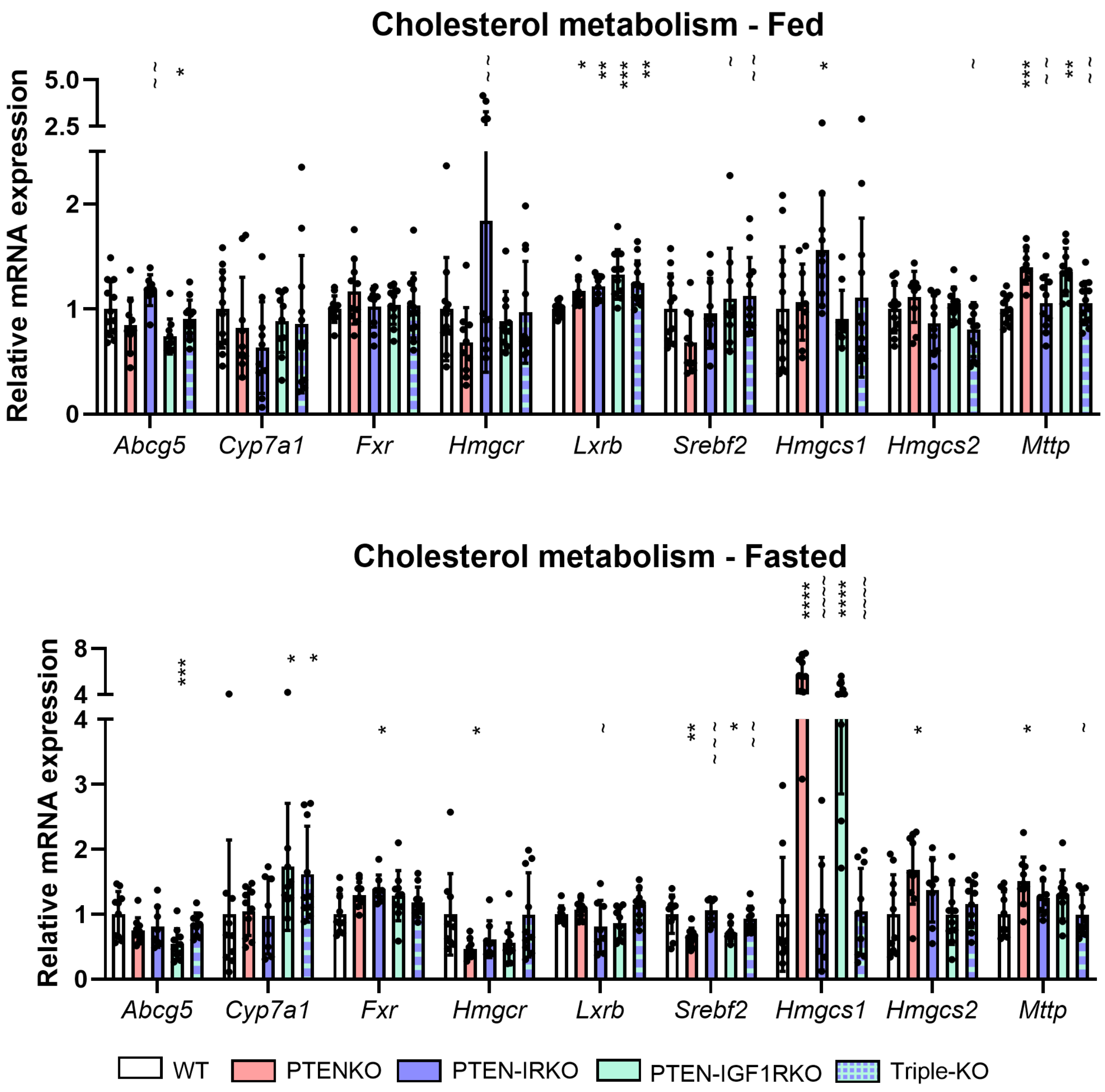

Fig. S2: Hepatic expression of key enzymes involved in cholesterol metabolism.

mRNA expressions of key genes involved in cholesterol metabolism (fed state: CTRL n=12, PTENKO n=10, PTEN-IRKO n=10, PTEN-IGF1RKO n=10, triple-KO n=12; fasted state: CTRL n=10, PTENKO n=10, PTEN-IRKO n=8, PTEN-IGF1RKO n=10, triple-KO n=10) in the livers of 4 months old mice under fed (upper panels) and overnight fasted conditions (bottom panels). Outliers were removed following ROUT (Q=1%) test and one-way ANOVA was performed. Values are represented as mean +/- SD. Values were considered significant when compared to CTRL (*) and to PTENKO (~):
*/~ p≤ 0.05, **/~~ p≤ 0.01,***/~~~ p≤ 0.001 or ****/~~~~ p≤ 0.0001.

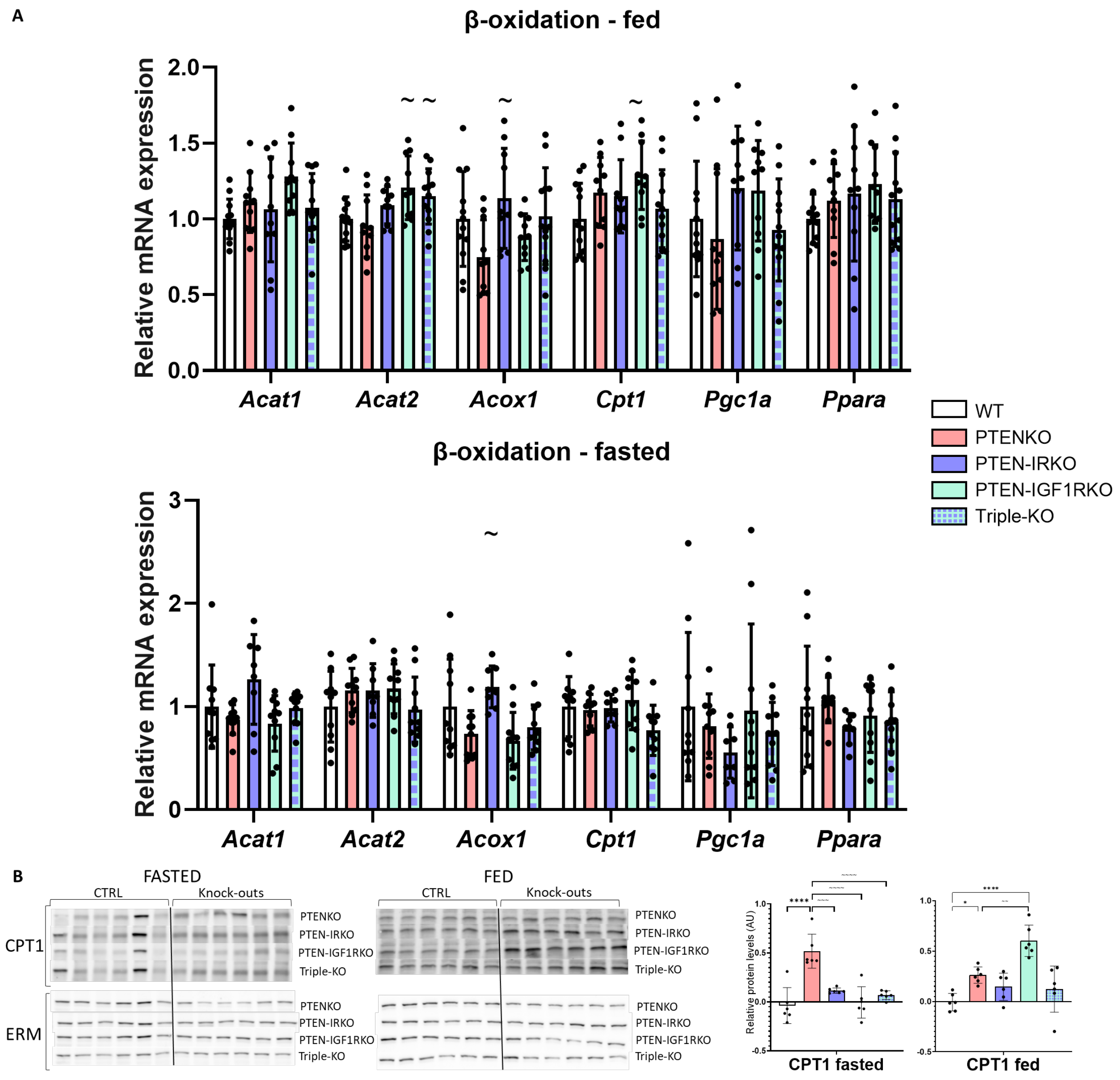

Fig. S3: Hepatic expression of key enzymes involved in β-oxidation.

(A) Hepatic β-oxidation was investigated under fasted or fed conditions in CTRL, PTENKO, PTEN-IRKO, PTEN-IGF1RKO and triple-KO mice at 4 months of age *via* RT-qPCR of *Acat1, Acat2, Acox1, Cpt1, Pgc1a* and *Ppara*. Values are represented as mean ± SD of 8-12 mice per group.

(B) CPT1 protein levels were determined *via* Western blot in CTRL, PTENKO, PTEN-IRKO, PTEN-IGF1RKO and triple-KO livers under fasted or fed conditions. ERM was used as standardization protein (run at the same time as Figure 2B – same normalization blot, 6 mice per group). Values are represented as mean ± SD.

Outliers were removed following ROUT (Q=1%) test and one-way ANOVA was performed. Values were considered significant when compared to CTRL (*) and to PTENKO (~):
*/~ p≤ 0.05, **/~~ p≤ 0.01, ***/~~~ p≤ 0.001 or ****/~~~~ p≤ 0.0001.

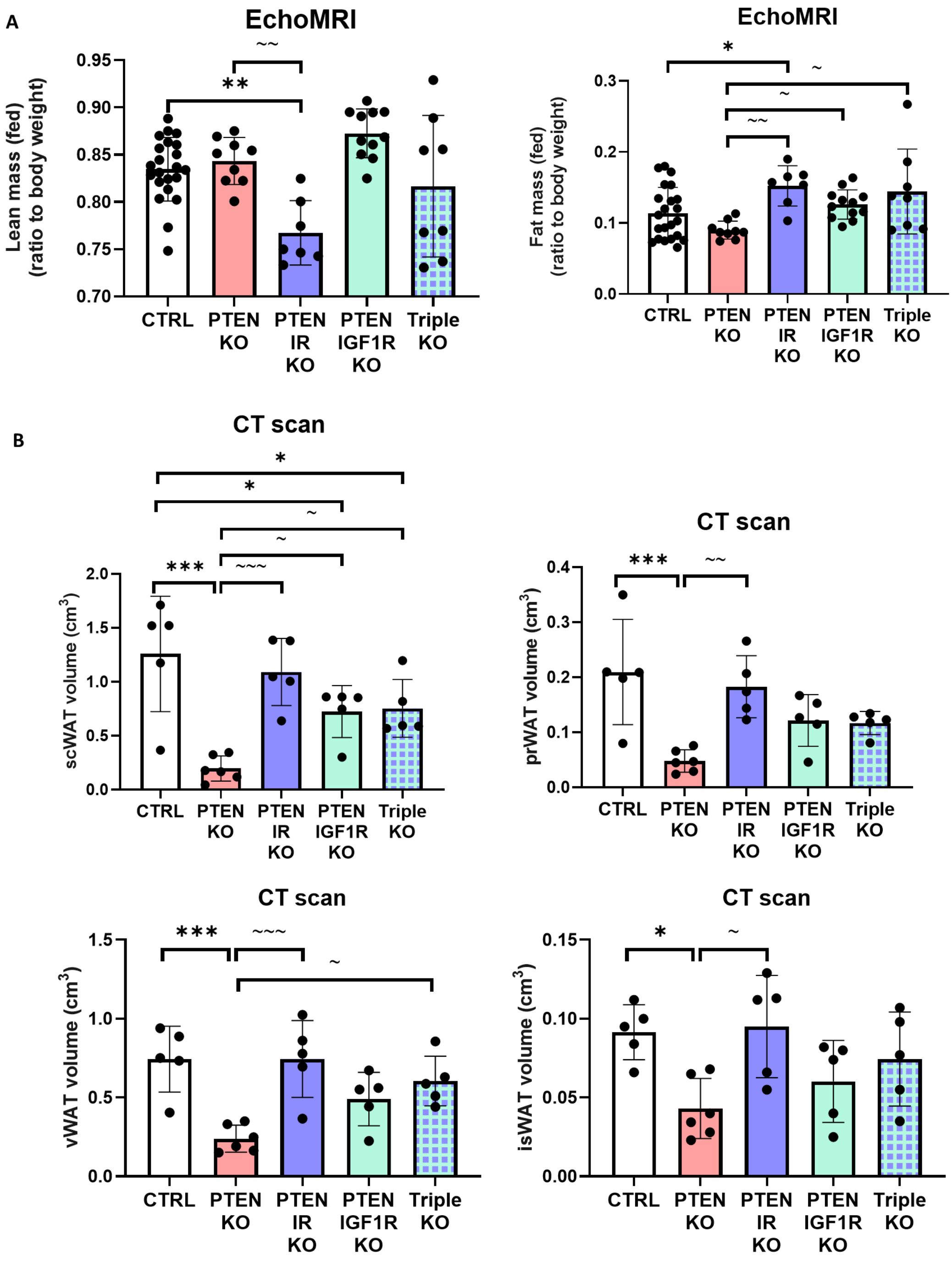

Fig. S4: Echo-MRI analysis and CT-scan analysis of different white adipose tissue depots.

(A) Total lean mass and fat mass determined through Echo-MRI.

(B) scWAT – subcutaneous white adipose tissue, prWAT – perirenal white adipose tissue, vWAT – visceral white adipose tissue and isWAT – inguinal subcutaneous white adipose tissue volumes in CTRL, PTENKO, PTEN-IRKO, PTEN-IFG1RKO and PTEN-IR-IFG1RKO (triple-KO) mice under fed conditions determined through CT-scan (n=5-6/group).

Outliers were removed following ROUT (Q=1%) test and one-way ANOVA was performed. Values are represented as mean ±SD and were considered significant when compared to CTRL (*) and to PTENKO (~):
*/~ p≤ 0.05, **/~~ p≤ 0.01, ***/~~~ p≤ 0.001 or ****/~~~~ p≤ 0.0001.

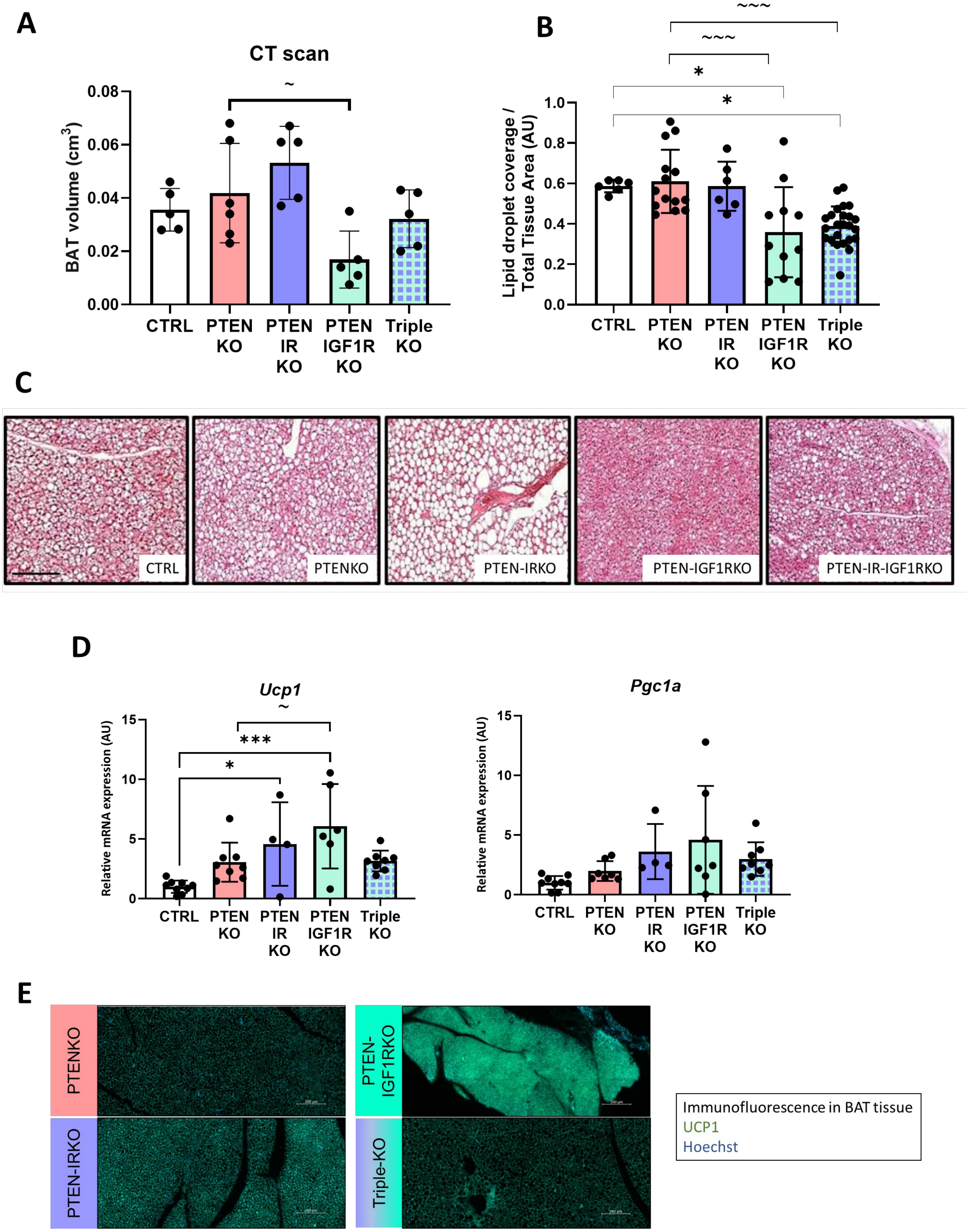

Fig. S5: Analysis of the brown adipose tissue in the different mutants.

(A) Volume of interscapular brown adipose tissue (BAT) as determined by CT-scan analyses in CTRL (n=5), PTENKO (n=6), PTEN-IRKO (n=5), PTEN-IGF1RKO (n=5), and triple-KO (n=5) mice.

(B) Quantification of lipid droplet area over total BAT area in CTRL (n=6), PTENKO (n=14), PTEN-IRKO (n=6), PTEN-IGF1RKO (n=11), and triple-KO (n=24) mice. H&E stained sections from BAT were scanned using AxioScan and analyzed using the QuPath software. Following tissue manual detection, the pixel classification tool was used to classify positive areas within the established ROI (channel: red; prefilter: gaussian; smoothing sigma: 2). Lipid droplets were considered as empty spaces (negative areas) within the region of interest (ROI) and calculated based on the following: 1- (positive area/total tissue area).

(C) Representative histological sections (H&E staining) of BAT sections.

(D) mRNA expressions of UCP1 and PGC1a (CTRL n=9, PTENKO n=8, PTEN-IRKO n=4, PTEN-IGF1RKO n=6, triple-KO n=8) analyzed through RT-qPCR.

(E) Representative images of UCP1 immunofluorescence in the BAT of CTRL, PTENKO, PTEN-IRKO, PTEN-IGF1RKO, and triple-KO mice (green-UCP1, blue – Hoechst).

Outliers were removed following ROUT (Q=1%) test and one-way ANOVA was performed. Values were considered significant when compared to CTRL (*) and to PTENKO (~):
*/~ p≤ 0.05, **/~~ p≤ 0.01, ***/~~~ p≤ 0.001 or ****/~~~~ p≤ 0.0001.

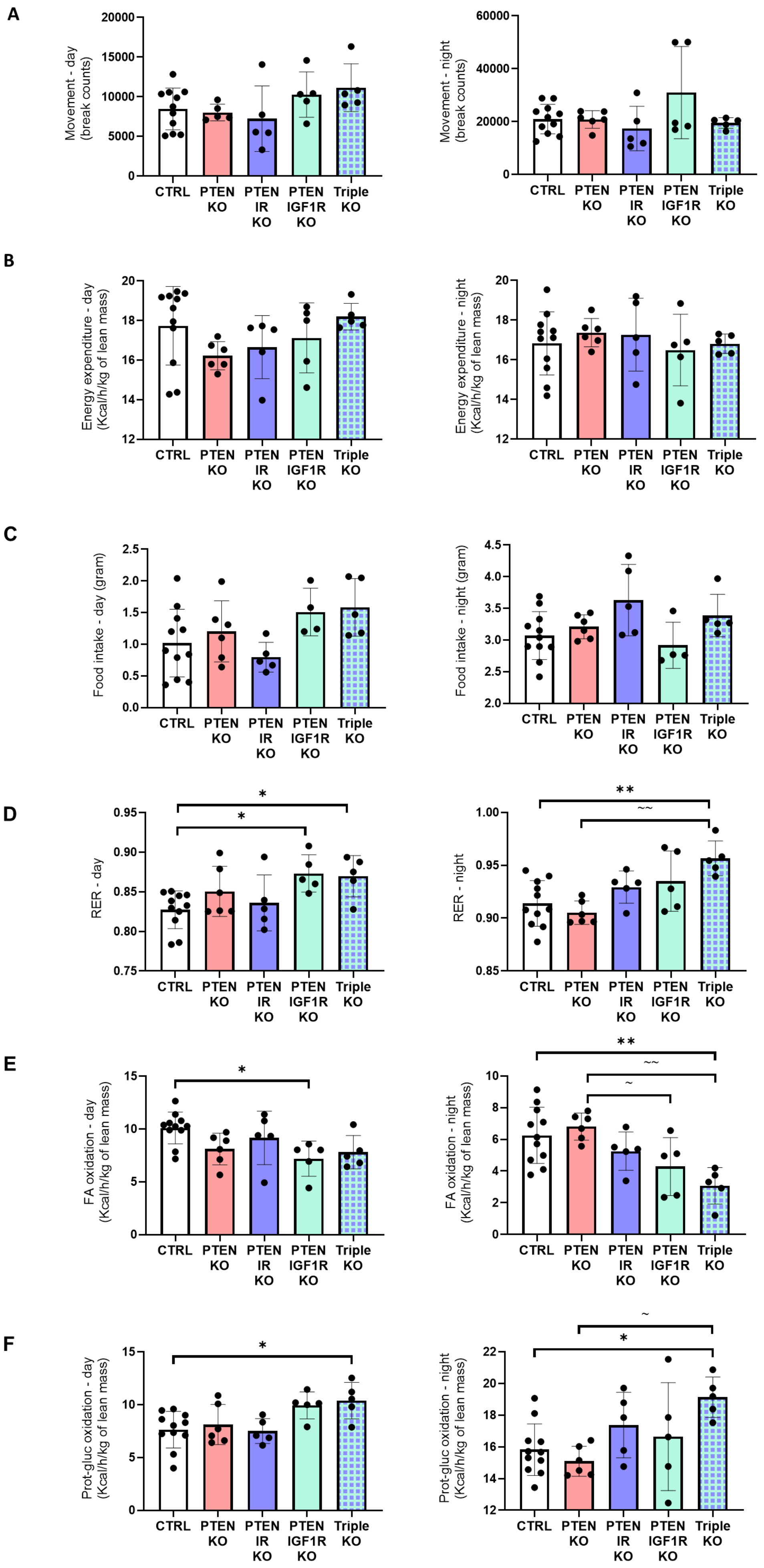

A
Movement - day (break counts)
Movement - night (break counts)
B
Energy expenditure - day (Kcal/h/kg of lean mass)
Energy expenditure - night (Kcal/h/kg of lean mass)
C
Food intake - day (gram)
Food intake - night (gram)
D
RER - day
RER - night
E
FA oxidation - day (Kcal/h/kg of lean mass)
FA oxidation - night (Kcal/h/kg of lean mass)
F
Prot-gluc oxidation - day (Kcal/h/kg of lean mass)
Prot-gluc oxidation - night (Kcal/h/kg of lean mass)
CTRL
PTEN KO
PTEN IR KO
PTEN IGF1R KO
Triple KO

Fig. S6: Metabolic phenotyping of the different mutants.

(A) Physical activity - movement, (B) energy expenditure, (C) food consumption, (D) Respiratory Exchange Ratio (RER) (E) fatty acid consumption and (F) protein and glucose oxidation were recorded for 24 hours using Labmaster metabolic cages. Graphs represent values obtained during the day (left panels) vs during the night (right panels). Values are mean (± SD) of 5-11 mice per group. Outliers were removed following ROUT (Q=1%) test and one-way ANOVA was performed. Values were considered significant when compared to CTRL (*) and to PTENKO (~):
*/~ p≤ 0.05, **/~~ p≤ 0.01, ***/~~~ p≤ 0.001 or ****/~~~~ p≤ 0.0001.

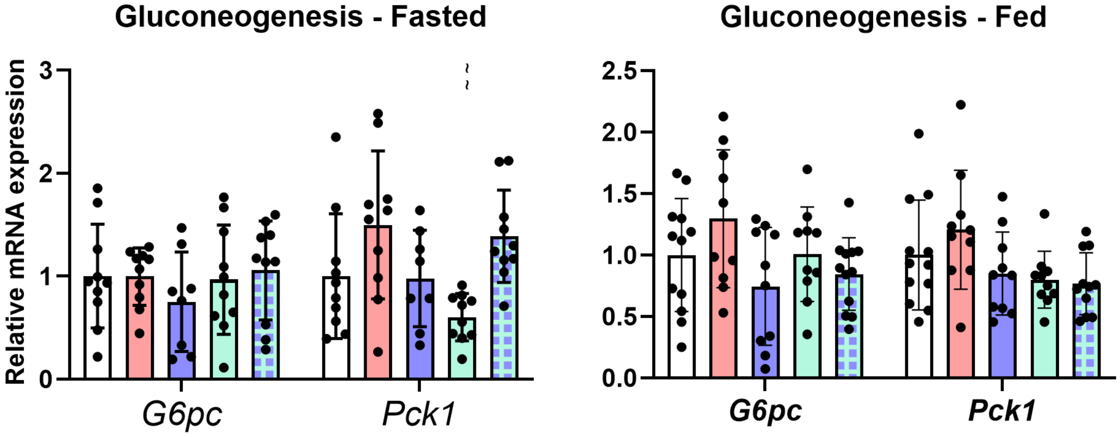

Fig. S7: Hepatic gluconeogenic gene expression in the different mutants in fasted and fed conditions.

Hepatic gluconeogenic gene expression levels were investigated in fasted conditions (left panel, CTRL n=10, PTENKO n=10, PTEN-IRKO n=8, PTEN-IGF1RKO n=10, and triple-KO n=10), as well as in fed conditions (right panel, CTRL n=12, PTENKO n=10, PTEN-IRKO n=10, PTEN-IGF1RKO n=10, and triple-KO n=12), at 4 months of age *via* RT-qPCR. Values are represented as fold-change to CTRL (± SD). Outliers were removed following ROUT (Q=1%) test and one-way ANOVA was performed. Values were considered significant when compared to CTRL (*) and to PTENKO (~):
*/~ p≤ 0.05, **/~~ p≤ 0.01, ***/~~~ p≤ 0.001 or ****/~~~~ p≤ 0.0001.

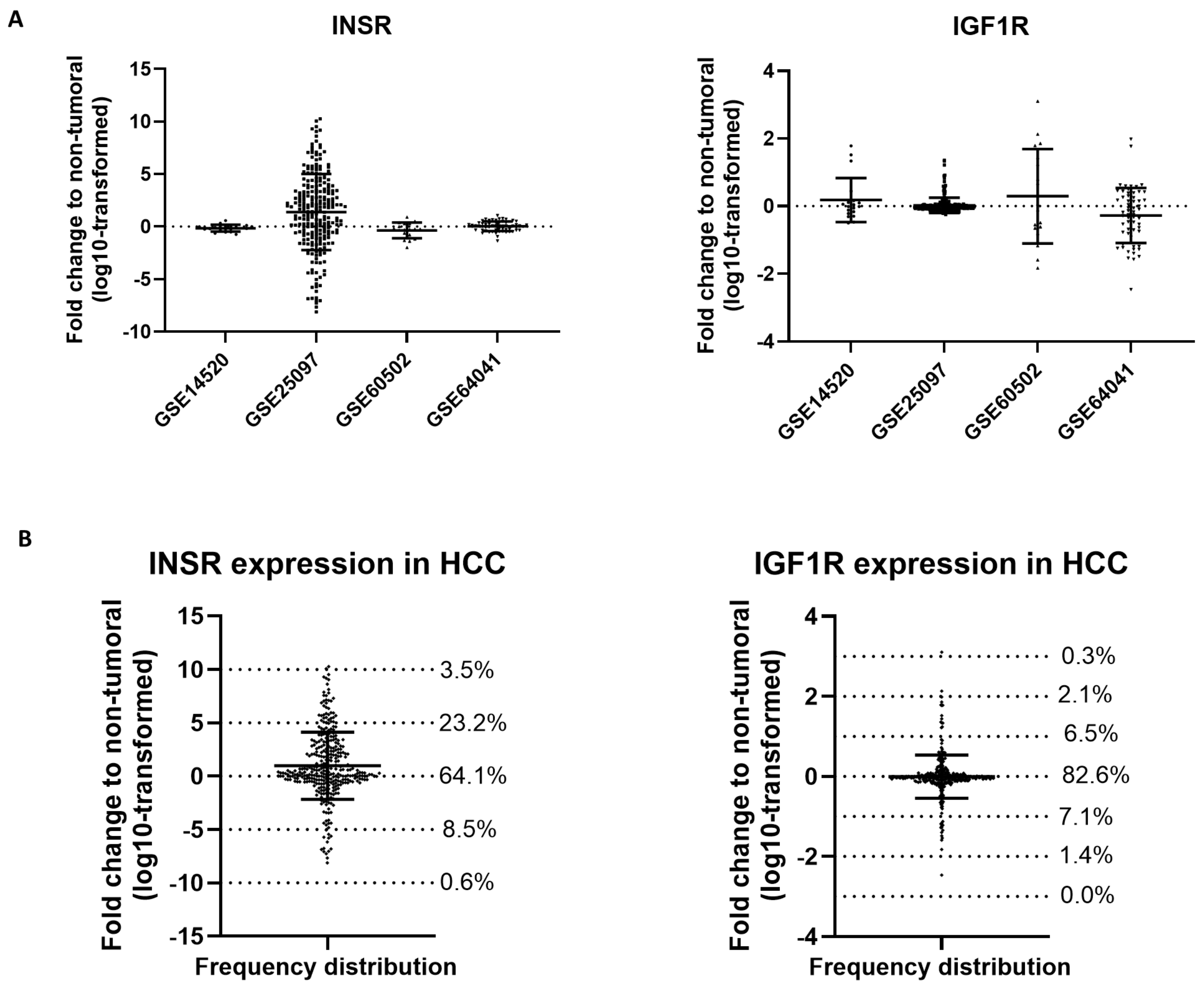

Fig. S8: INSR and IGF1R mRNA expression in HCC *versus* non-tumoral liver.

(A) The mRNA expression levels of INSR and IGF1R were determined in 4 different GEOdatasets of HCC patients (GSE14520, n=19, GSE25097, n=243, GSE60502, n=18, GSE64041, n=60) and the fold change in expression between HCC and non-tumoral biopsy was determined for each patient. Expression data are presented as log10 transformed.

(B) The data from the 4 GEOdatasets were pooled together and frequency distribution was calculated (INSR: relative frequency percentages, bin=5 [-10,10]; IGF1R: relative frequency-percentages, bin=1 [-3,3].

Data is represented as mean+/-SD.

	GS follow link to MSigDB	GS DETAILS	SIZE	ES	NES	NOM p-val	FDR q-val	FWER p-val	RANK AT MAX	LEADING EDGE
1	HALLMARK_BILE_ACID_METABOLISM	Details ...	112	-0.78	-1.69	0.000	0.020	0.031	1512	tags=50%, list=7%, signal=54%
2	HALLMARK_FATTY_ACID_METABOLISM	Details ...	153	-0.70	-1.66	0.002	0.021	0.060	2118	tags=57%, list=10%, signal=63%
3	HALLMARK_PEROXISOME	Details ...	101	-0.65	-1.58	0.000	0.044	0.149	2026	tags=48%, list=10%, signal=52%
4	HALLMARK_XENOBIOTIC_METABOLISM	Details ...	196	-0.70	-1.52	0.010	0.073	0.286	1442	tags=49%, list=7%, signal=52%
5	HALLMARK_ADIPOGENESIS	Details ...	193	-0.50	-1.40	0.063	0.166	0.533	2525	tags=39%, list=12%, signal=45%
6	HALLMARK_OXIDATIVE_PHOSPHORYLATION	Details ...	194	-0.56	-1.40	0.107	0.141	0.537	2492	tags=35%, list=12%, signal=40%
7	HALLMARK_COAGULATION	Details ...	136	-0.56	-1.31	0.102	0.211	0.710	1285	tags=34%, list=6%, signal=36%
8	HALLMARK_INTERFERON_ALPHA_RESPONSE	Details ...	97	-0.58	-1.23	0.253	0.287	0.844	2544	tags=48%, list=12%, signal=55%
9	HALLMARK_KRAS_SIGNALING_DN	Details ...	192	-0.32	-1.23	0.128	0.258	0.847	3081	tags=19%, list=15%, signal=22%
10	HALLMARK_PANCREAS_BETA_CELLS	Details ...	39	-0.38	-1.20	0.211	0.269	0.882	880	tags=10%, list=4%, signal=11%
11	HALLMARK_ANDROGEN_RESPONSE	Details ...	97	-0.39	-1.17	0.153	0.290	0.909	1813	tags=28%, list=9%, signal=30%
12	HALLMARK_HEME_METABOLISM	Details ...	194	-0.30	-1.03	0.363	0.461	0.976	2899	tags=24%, list=14%, signal=27%
13	HALLMARK_COMPLEMENT	Details ...	194	-0.36	-1.02	0.427	0.456	0.983	2234	tags=24%, list=11%, signal=27%
14	HALLMARK_INTERFERON_GAMMA_RESPONSE	Details ...	200	-0.42	-1.00	0.456	0.443	0.984	1968	tags=28%, list=10%, signal=31%
15	HALLMARK_CHOLESTEROL_HOMEOSTASIS	Details ...	73	-0.41	-0.99	0.457	0.439	0.990	1492	tags=27%, list=7%, signal=29%

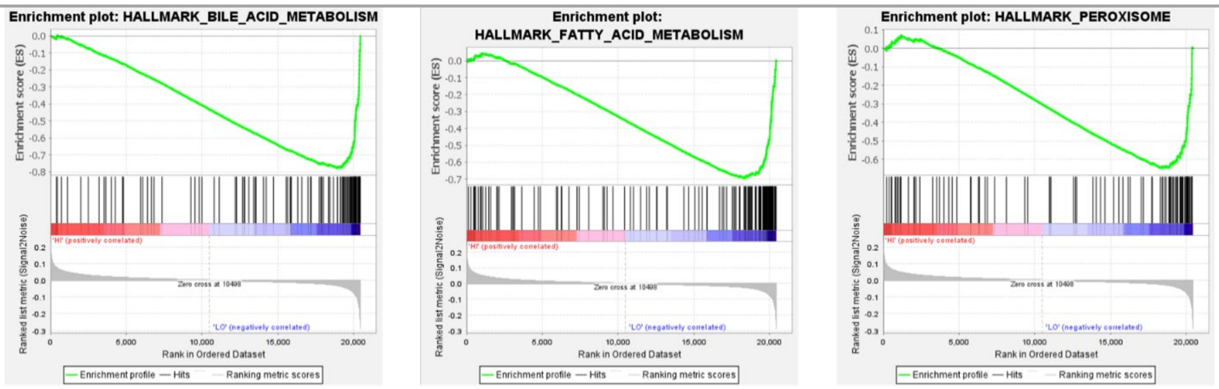

Gene sets enriched in phenotype LOW IGF1R

Fig. S9: Gene set enrichment analysis in HCC.

Gene set enrichment analysis was performed in the GSE36376 dataset containing expression analysis from human HCC (240 human HCC samples, segregated between the 20% most expressing *IGF1R* and the rest 80% of the samples). Enrichment was considered as significant if FDR q-value < 0.05. The three significantly enriched processes in low-IGF1R are highlighted in blue in the table and the enrichment plots are represented below.

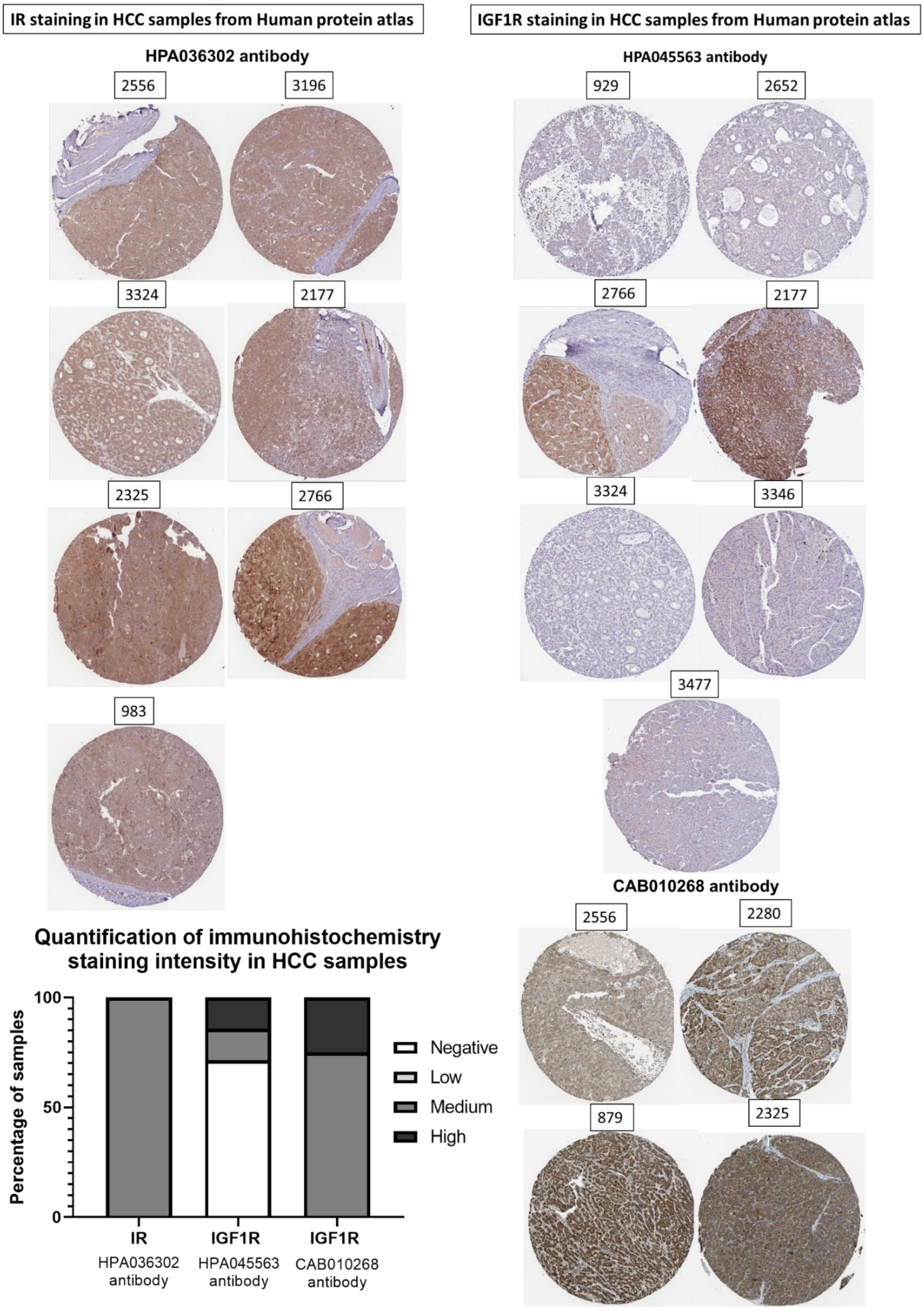
IR staining in HCC samples from Human protein atlas
HPA036302 antibody
2556
3196
3324
2177
2325
2766
983
IGF1R staining in HCC samples from Human protein atlas
HPA045563 antibody
929
2652
2766
2177
3324
3346
3477
CAB010268 antibody
2556
2280
879
2325
Quantification of immunohistochemistry staining intensity in HCC samples
Percentage of samples
100
50
0
IR
HPA036302 antibody
IGF1R
HPA045563 antibody
IGF1R
CAB010268 antibody
Negative
Low
Medium
High

Fig. S10: Immunohistochemistry of IR and IGF1R in HCC samples from the Human Protein Atlas.

Images for IR or IGF1R immunohistochemistry staining in biopsies from HCC patients were obtained from the *Human Protein Atlas* database (proteinatlas.org). The ID for each patient is indicated above the immunohistochemistry image (black box).

The evaluation of the intensity of the staining (negative, low, medium or high) was done by the *Human Protein Atlas* database and a graphical representation was done, showing the percentage of different intensity levels for each staining.

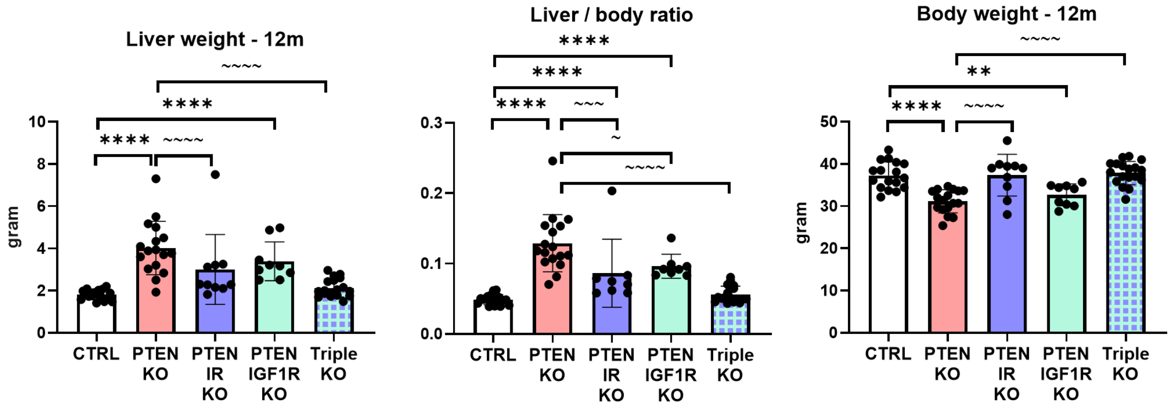

Fig. S11: Liver and body weight at 12 months of age.

Ex vivo assessment of liver weight (left panel), liver weight presented as percentage of body weight (middle panel), and body weight (right panel) in 12 months old CTRL, PTENKO, PTEN-IRKO, PTEN-IGF1RKO and triple-KO mice. Outliers were removed following ROUT (Q=1%) test and one-way ANOVA was performed. Values were considered significant when compared to CTRL (*) and to PTENKO (~):

*/~ p≤ 0.05, **/~~ p≤ 0.01,***/~~~ p≤ 0.001 or ****/~~~~ p≤ 0.0001.

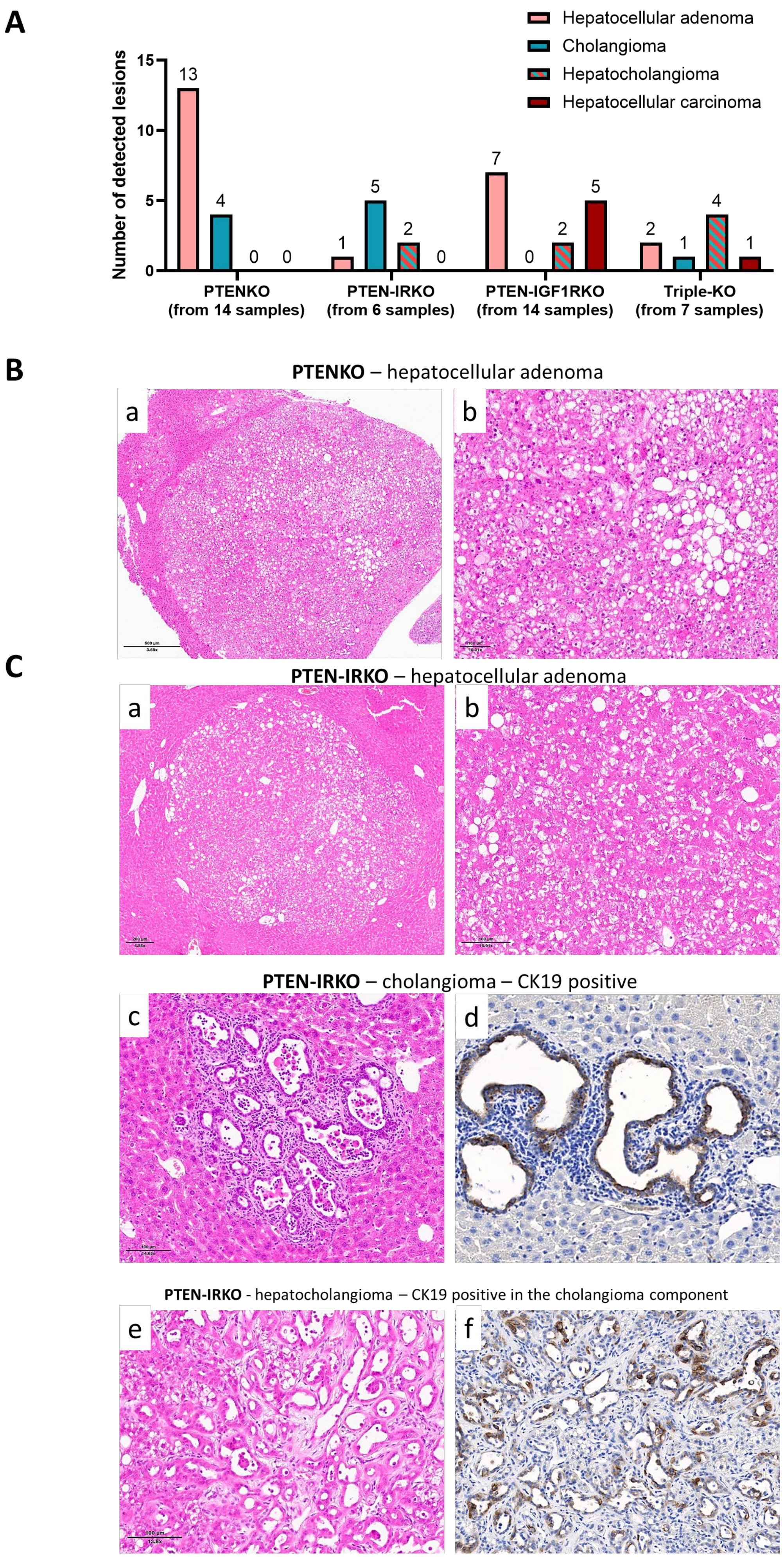
A
Hepatocellular adenoma
Cholangioma
Hepatocholangioma
Hepatocellular carcinoma
Number of detected lesions
15
10
5
0
13
4
0
0
1
5
2
0
7
0
2
5
2
1
4
1
PTENKO (from 14 samples)
PTEN-IRKO (from 6 samples)
PTEN-IGF1RKO (from 14 samples)
Triple-KO (from 7 samples)
B
PTENKO – hepatocellular adenoma
a
b
C
PTEN-IRKO – hepatocellular adenoma
a
b
PTEN-IRKO – cholangioma – CK19 positive
c
d
PTEN-IRKO - hepatocholangioma – CK19 positive in the cholangioma component
e
f

D **PTEN-IGF1RKO** – hepatocellular carcinoma

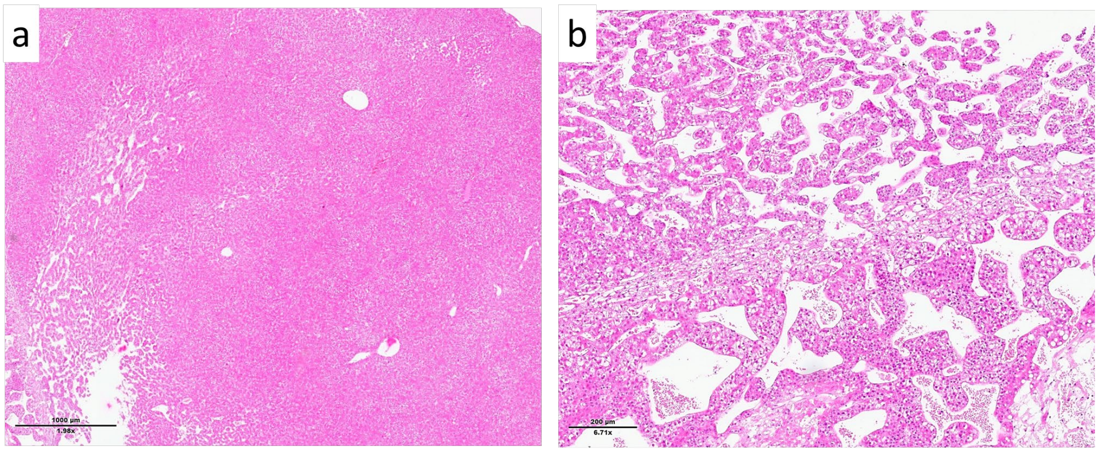

E **Triple-KO** – hepatocholangioma – CK19 positive in the cholangioma component

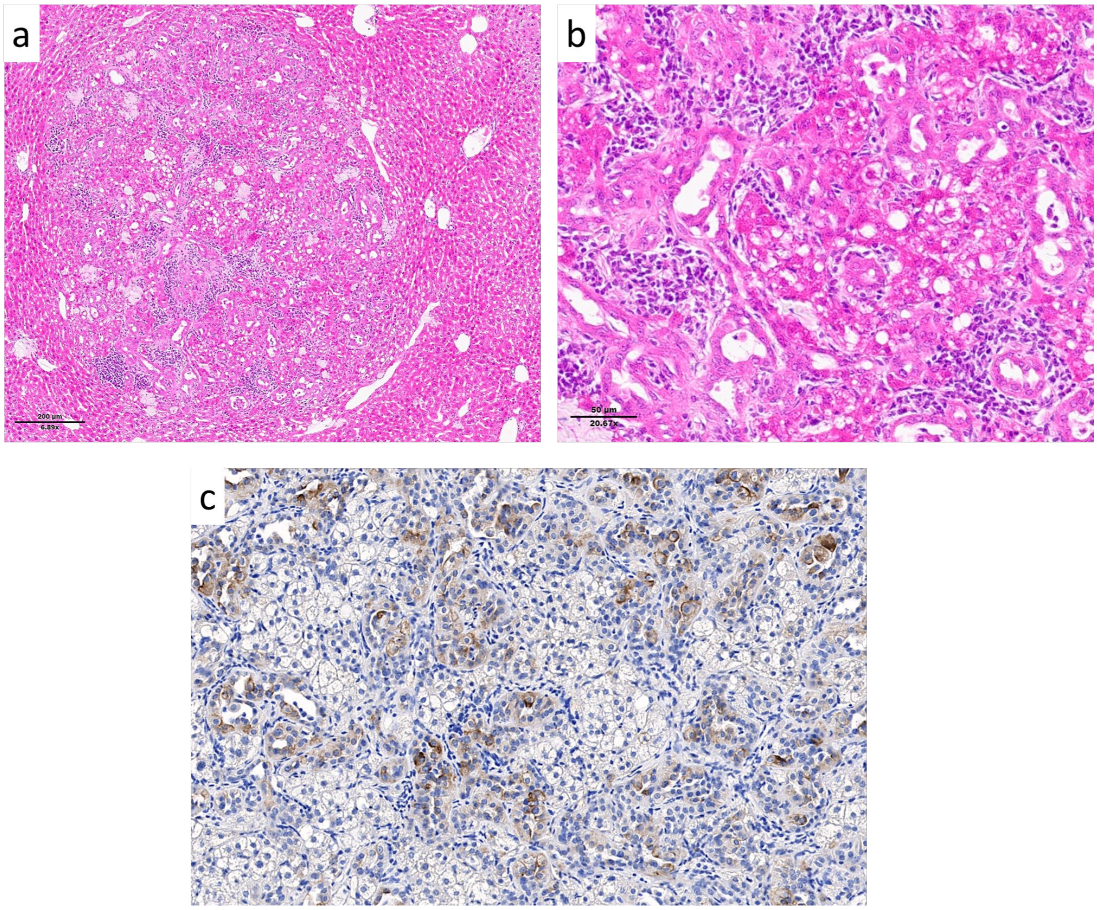

Fig. S12: Hepatic tumor incidence and histology.

H&E staining and CK19 immunohistochemistry of livers/hepatic tumors of 12 months-old PTENKO, PTEN-IRKO, PTEN-IGF1RKO and triple-KO mice.

(A) Incidence rate of different types of tumors in the mutants analyzed by histological assessment. Hepatic samples were randomly selected (PTENKO n=14, PTEN-IRKO n=6 PTEN-IGF1RKO n=14, triple-KO n=7). Representative images from each group are shown below (panels B-E).

(B) PTENKO mice: Representative H&E staining images of liver cell adenoma at (a) OMx3.68 and at (b) OMx10, negative for CK19: a nodule of well-differentiated hepatocytes with borders pushing towards the adjacent parenchyma. The liver cell plates are thicker than normal but no larger than two or three cells at the maximum. Hepatocytes have a large clear cytoplasm.

(C) PTEN-IRKO mice: Representative H&E staining images of liver cell adenoma at (a) OMx4.55 and at (b) OMx16, negative for CK19; (c) cholangioma at OMx14.6, (d) positive for CK19; and (e) hepatocholangioma at OMx13.6, (f) positive for CK19 staining in the cholangioma department. The mixed hepatobiliary tumors are a mixture of irregular ductular bile structures lined either by cuboïdal cells or by thin small cells and of liver cell plates or nests with well-differentiated hepatocytes showing either an eosinophilic cytoplasm or some steatosis.

(D) PTEN-IGF1RKO mice: a) Hepatocellular carcinoma at (a) OMx1.98 and (b) OMx6.71, negative for CK19. Large nodule composed of malignant hepatocytes arranged in thick trabeculae along large vascular channels. Hepatocytes have a large eosinophilic cytoplasm and there is some nuclear polymorphism. Mitoses are found. There are areas of necrosis and the lesion is not well delimitated.

(E) Triple-KO mice. Hepatocholangioma at (a) OMx6.89 and (b) OMx20.87, (c) CK19 positive in the cholangioma component.

Regarding the CK19 immunohistochemistry staining: Antigen retrieval was done with citrate buffer pH 6, followed by 15 min treatment H_2O_2 3%. The saturation of the slides was done with 10% NGS. CK19 primary antibody was incubated overnight at 4°C. The next day, secondary HRP antibody was used and staining was revealed with the Abcam DAB kit.

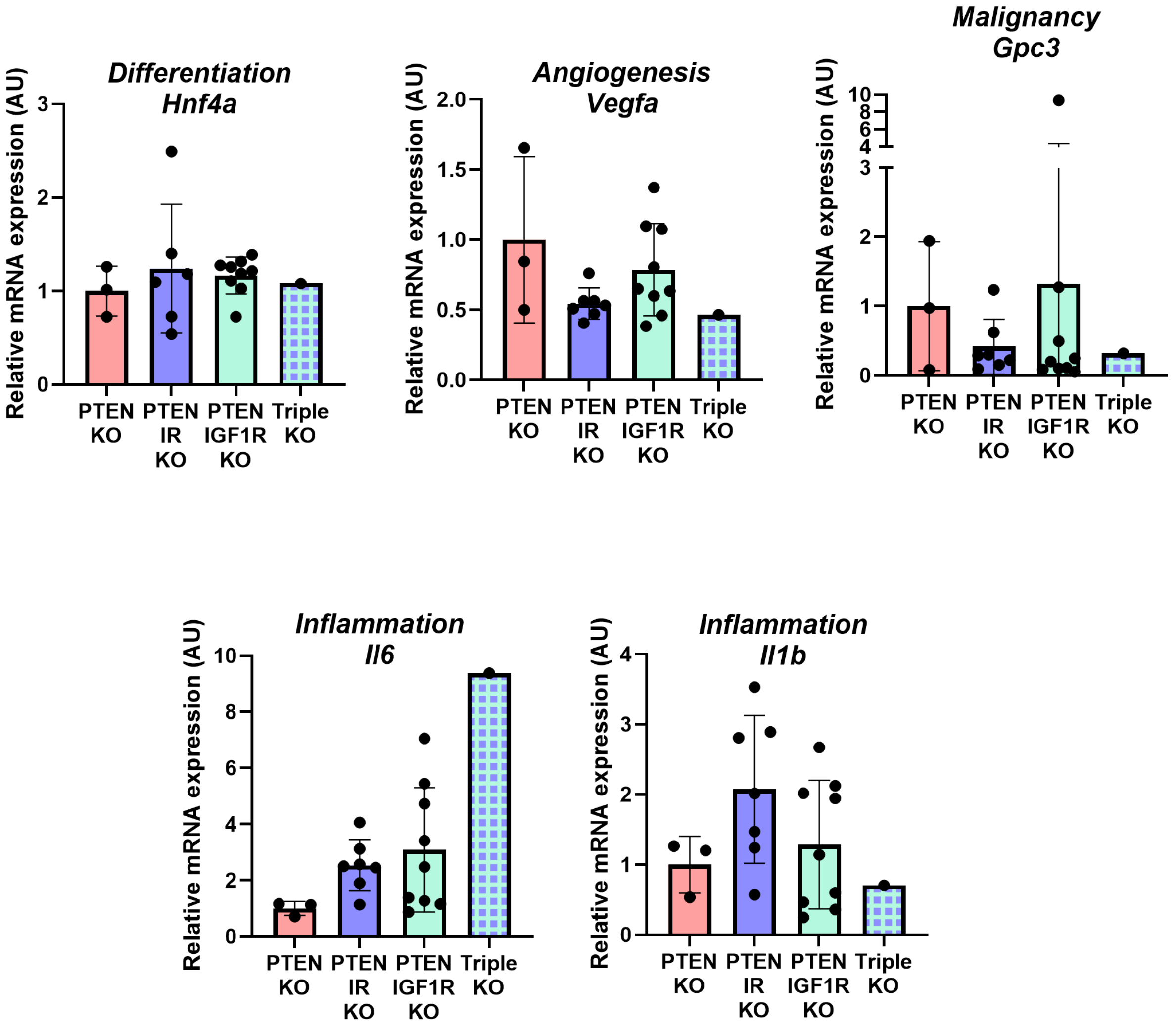

Fig. S13: Cancer marker expression in dissected tumors.

mRNA expression of *Hnf4, Vegfa, Gpc3, Il6 and Il1b* was investigated under fed conditions in dissected tumor samples of PTENKO (n=3), PTEN-IRKO (n=7), PTEN-IGF1RKO (n=9) and triple-KO (n=1) mice at 12 months of age *via* RT-qPCR. Outliers were removed following ROUT (Q=1%) test and one-way ANOVA was performed. Values are represented as mean +/- SD.

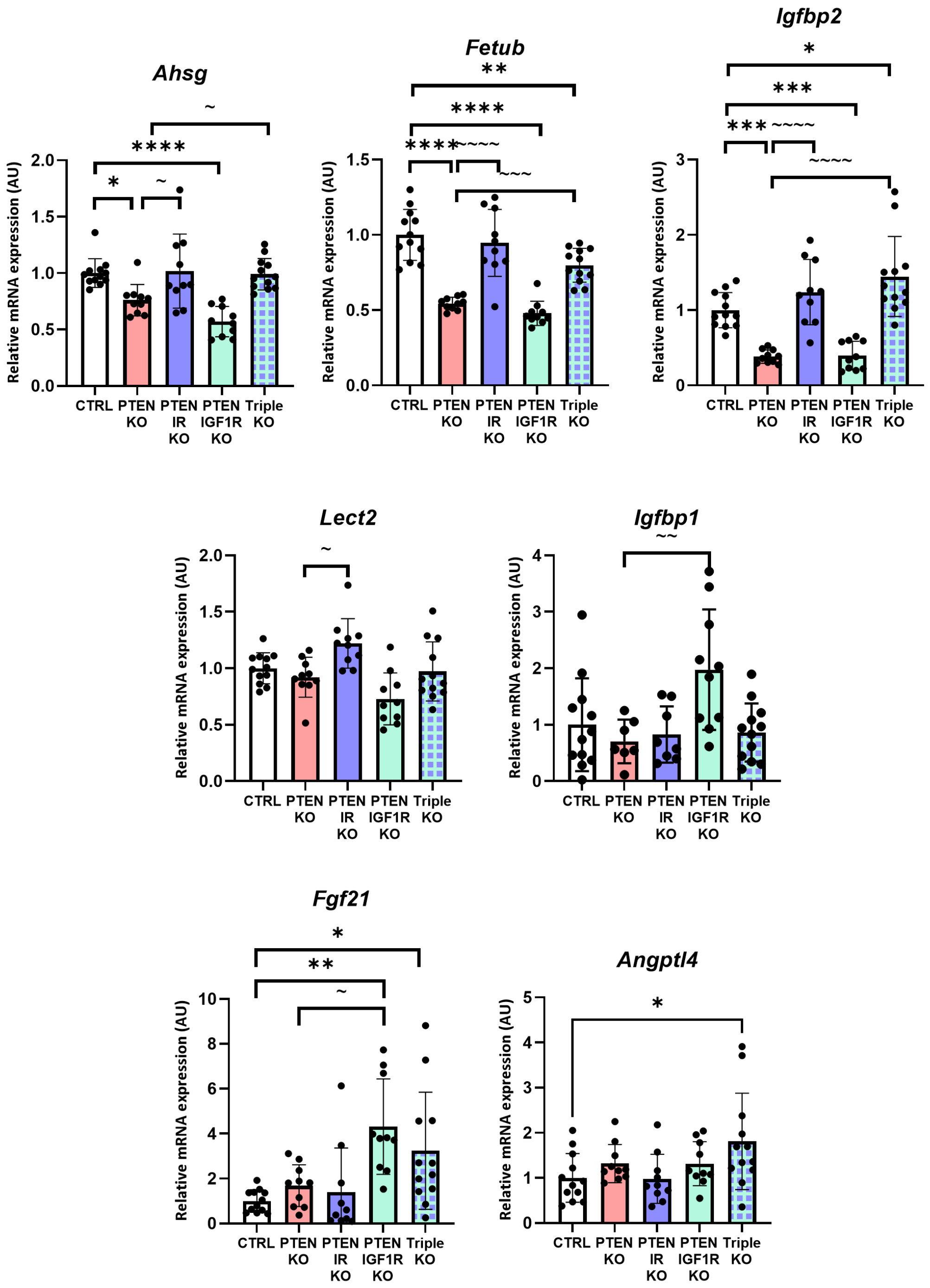

Fig. S14: Hepatokine expression in the liver.

Hepatokine mRNA expression was investigated under fed conditions in the livers of CTRL, PTENKO, PTEN-IRKO, PTEN-IGF1RKO and triple-KO mice at 4 months of age *via* RT-qPCR of *Ahsg* (Fetuin-A), *Fetub* (Fetuin-B), *Angptl4* (Angiopoietin-Like 4), *Igfbp1* (Insulin-like Growth Factor Binding Protein 1), *Igfbp2* (Insulin-like Growth Factor Binding Protein 2), *Lect2* (Leukocyte Cell Derived Chemotaxin 2) and *Fgf21* (Fibroblast Growth Factor 21). Values are mean +/- SD of 8-12 mice per group. Outliers were removed following ROUT (Q=1%) test and two-way ANOVA was performed, followed by a multiple comparison test. Values were considered significant when compared to CTRL (*) and to PTENKO (~): */~ p≤ 0.05, **/~~ p≤ 0.01,***/~~~ p≤ 0.001 or ****/~~~~ p≤ 0.0001.

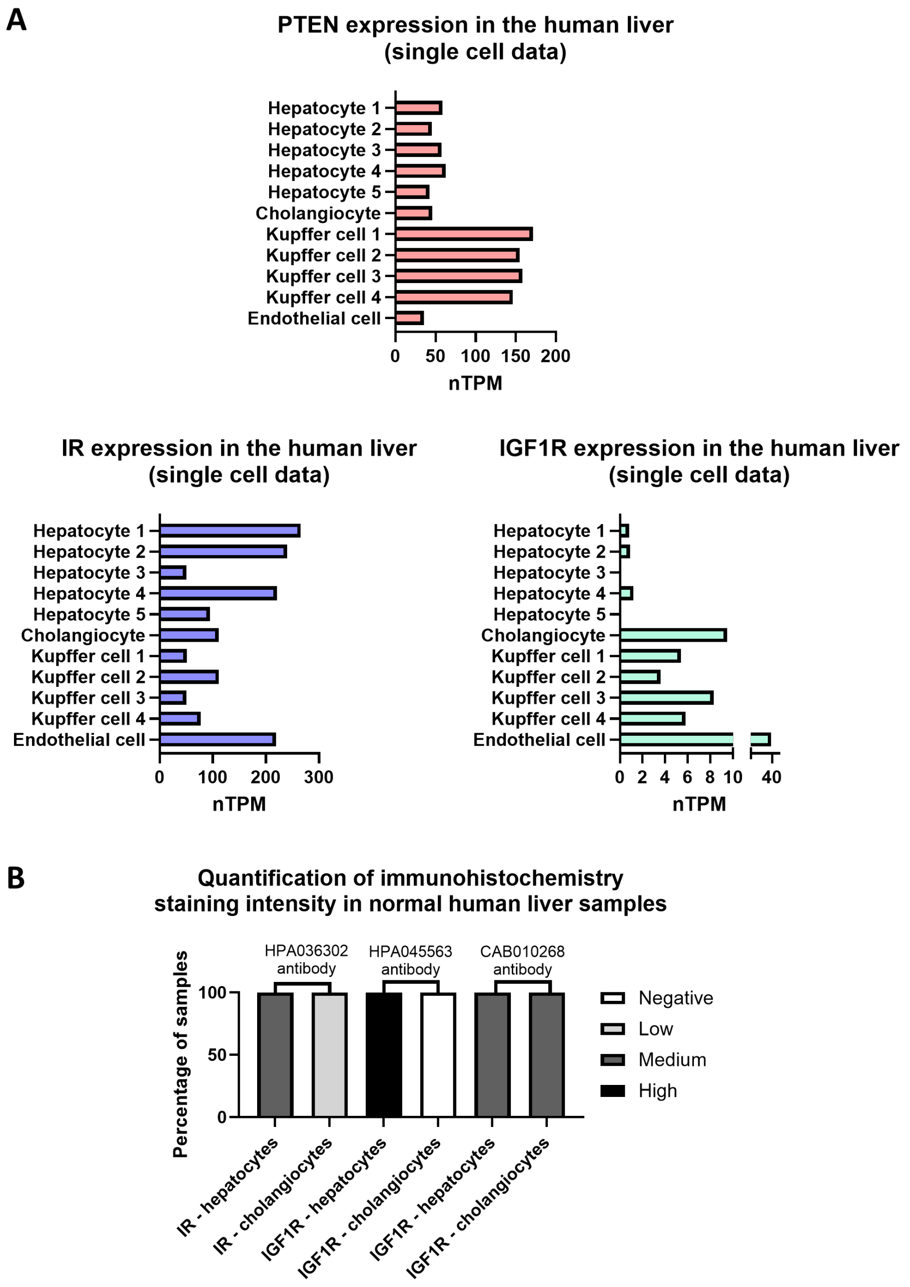

Fig. S15: RNA and protein levels of IGF1R and IR in hepatocytes and cholangiocytes in normal human liver.

(A) Single-cell RNA gene expression data for PTEN, IGF1R and INSR in human liver were obtained through the Human protein atlas database (proteinatlas.org). RNA expression is expressed in normalized transcript per million (nTPM).

(B) Protein levels of IR and IGF1R in human hepatocytes and cholangiocytes were assessed through immunohistochemistry of these proteins in healthy human liver samples, available in the publicly available human protein atlas database (proteinatlas.org). The evaluation of the intensity of the staining (negative, low, medium or high) was done by the Human Protein Atlas database and a graphical representation was done, showing the percentage of different intensity levels for each staining. For IGF1R, two different antibodies were used for immunohistochemistry (HPA045563 and CAB010268, scored separately).

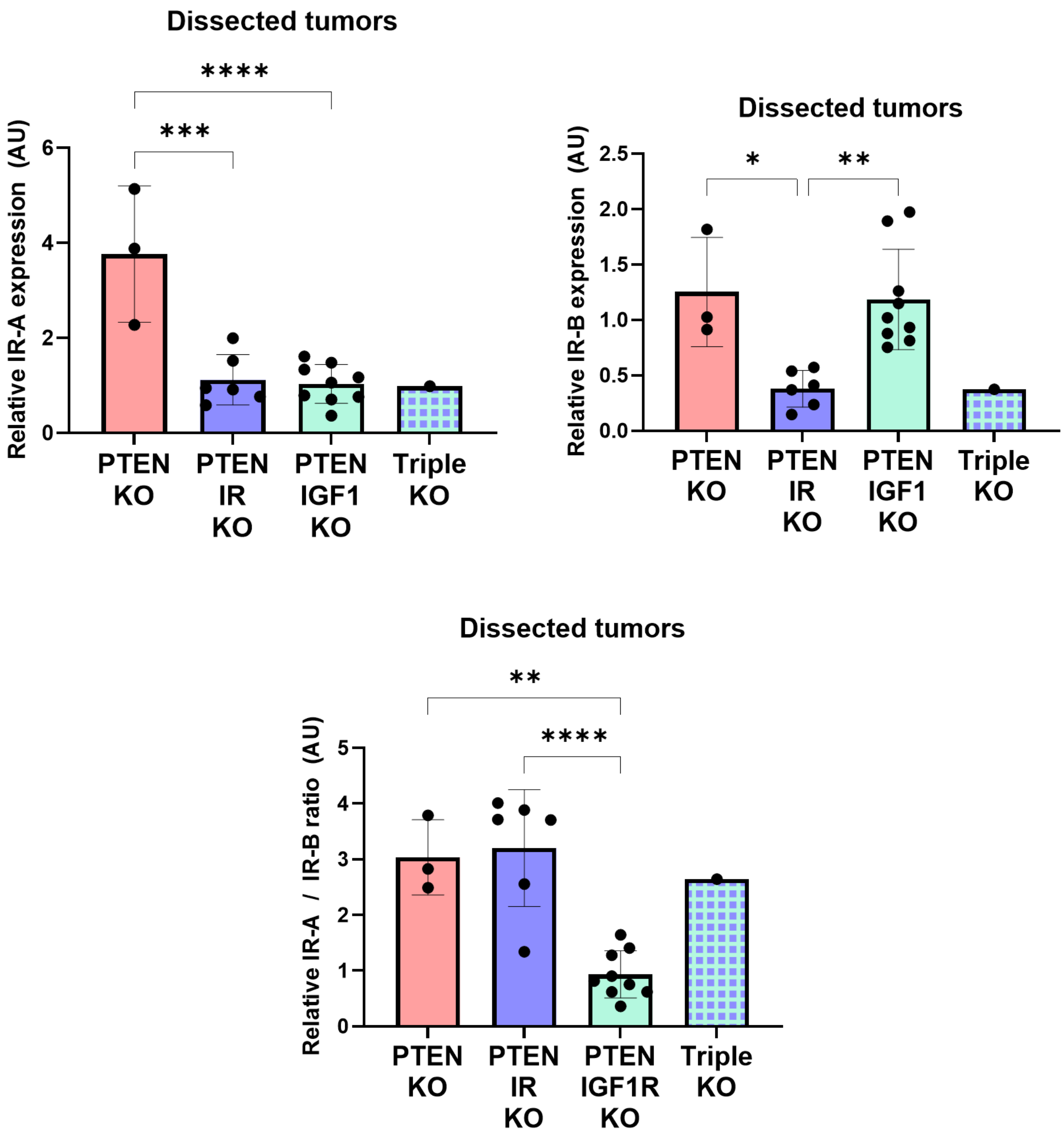

Fig. S16: IR-A and IR-B expression in the dissected tumors.

mRNA expression of IR-A and IR-B isoform and IR-A/IR-B ratio was investigated under fed conditions in dissected tumor samples of PTENKO, PTEN-IRKO, PTEN-IGF1RKO and triple-KO mice at 12 months of age *via* RT-qPCR. Outliers were removed following ROUT (Q=1%) test and one-way ANOVA was performed. Values are mean +/- SD of 1-9 mice per group. Values were considered significant when * p≤ 0.05, ** p≤ 0.01, *** p≤ 0.001 or **** p≤ 0.0001.

.

	PMID	Publication	Role
IGF1R in hepatocellular carcinoma	28619711	IGFBP7 Deletion Promotes Hepatocellular Carcinoma	Pro-oncogenic
	28202495	Expression of GRK2 and IGF1R in Hepatocellular Carcinoma: Clinicopathological and Prognostic Significance	Pro-oncogenic
	31732382	miR-455-5p Suppresses Hepatocellular Carcinoma Cell Growth and Invasion via IGF-1R/AKT/GLUT1 Pathway by Targeting IGF-1R	Pro-oncogenic
	30360707	Insulin-like Growth Factor I Receptor: A Novel Target for Hepatocellular Carcinoma Gene Therapy	Pro-oncogenic
	28624790	Co-targeting of IGF1R/mTOR Pathway by miR-497 and miR-99a Impairs Hepatocellular Carcinoma Development	Pro-oncogenic
	26658144	Tumour Initiating Cells and IGF/FGF Signalling Contribute to Sorafenib Resistance in Hepatocellular Carcinoma	Pro-oncogenic
	31267557	GSTZ1-1 Deficiency Activates NRF2/IGF1R Axis in HCC via Accumulation of Oncometabolite Succinylacetone	Pro-oncogenic
	29156819	miR-29a-3p Suppresses Cell Proliferation and Migration by Downregulating IGF1R in Hepatocellular Carcinoma	Pro-oncogenic
	31160483	MiR-505 Suppressed the Growth of Hepatocellular Carcinoma Cells via Targeting IGF-1R	Pro-oncogenic
	27126374	Abrogating the Interplay Between IGF2BP1, 2 and 3 and IGF1R by let-7i Arrests Hepatocellular Carcinoma Growth	Pro-oncogenic
	29881824	Inhibition of insulin-like growth factor 1 receptor enhances the efficacy of sorafenib in inhibiting hepatocellular carcinoma cell growth and survival	Pro-oncogenic
	22895605	Inhibition of hepatocellular carcinoma cell growth by an anti-insulin-like growth factor-I receptor monoclonal antibody	Pro-oncogenic
	31114322	microRNA-944 Inhibits the Malignancy of Hepatocellular Carcinoma by Directly Targeting IGF-1R and Deactivating the PI3K/Akt Signaling Pathway	Pro-oncogenic

	PMID	Publication	Role
IR in hepatocellular carcinoma	30849481	Insulin receptor isoform A favors tumor progression in human hepatocellular carcinoma by increasing stem/progenitor cell features	Pro-oncogenic
	28710407	Role of insulin receptor substrates in the progression of hepatocellular carcinoma	Pro-oncogenic
	23633480	Mitogenic Insulin Receptor-A Is Overexpressed in Human Hepatocellular Carcinoma due to EGFR-Mediated Dysregulation of RNA Splicing Factors	Pro-oncogenic
	21411721	Upregulation of the Insulin Receptor and Type I Insulin-Like Growth Factor Receptor Are Early Events in Hepatocarcinogenesis	Pro-oncogenic

Table S1: Available literature on IR and IGF1R in hepatic carcinogenesis.

Supplementary materials and methods:

EchoMRI and CT-scan

Lean mass was measured using quantitative nuclear magnetic resonance analyzer EchoMRI-700 (Echo Medical Systems, Houston, TX). The fat depot distribution was imaged by multidetector CT-scan (Discovery 750 HD, GE Healthcare, Milwaukee, USA) and the volume was quantified by OsiriX imaging software (*Version 3.9*). The weight of major adipose depots was also investigated by dissection at the end of the experimental protocols.

Livers of 12 months aged mice were imaged by *in vivo* X-Ray computed tomography (CT scan) (Skysan 1076, Bruker, Belgium). Injection of the contrast agent ExiTron*TM* Nano12000 (MiltenyiBioTec, Germany) allowed the visualization of benign and malignant tumors. Liver volume, tumor number, and tumor volume were quantified by OsiriX imaging software (*Version 3.9*).

Glucose / pyruvate tolerance tests (GTT / PTT) and insulin injections

Mice were fasted overnight prior to intraperitoneal administration of glucose (1.5 g/kg) or pyruvate (2 g/kg). Blood glycemia was measured from tail vein during 2h.

Metabolic phenotyping

Indirect calorimetry, locomotor activity, food, and fluid intake were measured for 48 hours using Labmaster metabolic cages (TSE, Bad Homburg, Germany) after 3 days of adaptation prior to recording (mice were housed individually). Energy expenditure and respiratory exchange ratio were calculated as described [2].

Histological analyses

Tissue samples were fixed in 4% paraformaldehyde, embedded in paraffin, and cut into 5µm sections. For morphological investigations, hematoxylin/eosin staining was performed, and images were acquired using AxioScan.Z1 slide scanner (Zeiss, Germany).

Plasma and tissue analyses

Plasma glucose were determined by medical analysis laboratories accredited for the processing of patient samples (TEST Tailored Efficient Swiss Testing SA and AnaBio, Geneva, Switzerland). An automated Abott Architect analyzer (Abott Architect, Paris, France) was used to determine AST and ALT. Plasma insulin, glucagon as well as liver content of triglycerides, cholesteryl esters, and ketone bodies were measured with commercial kits (Supplementary Material and Methods). Hepatic glycogen content was assessed as previously described [3].

RNA extraction and real-time PCR

Trizol Reagent® was used to extract RNA and 1µg of RNA was reverse-transcribed using High-Capacity RNA-to-cDNA Kit (AppliedBiosystems). The quantitative real time PCR analysis was performed with the SYBR® Select Master Mix and the StepOne Plus® PCR system (Life Technologies). Primer sequences are listed in Supplementary Material and Methods. The following settings were used for the PCR amplifications: 40 cycles with: 94°C for 2 min, 60°C for 1 min, 68°C for 2 min. Results were normalized to the mRNA expression of cyclophilin A, β-actin and RPS9 using the geNorm algorithm.

Western blot analysis

Tissues were lysed in ice-cold RIPA buffer supplemented with a cocktail of protease inhibitors (Roche, Rotkreuz, Switzerland) using Tissue Lyser II (Qiagen, Düsseldorf, Germany). Proteins

were resolved by 5-20% gradient SDS-PAGE and blotted onto nitrocellulose membranes (Amersham, GE Healthcare, Buckinghamshire, UK). Detection of proteins was done with specific primary antibodies and HRP-conjugated secondary antibodies using chemoluminescence (Supplementary Material and Methods). The PXi system from SYNGENE (SYNOPTICS group, Cambridge, UK) and the GeneTools Software 4.03.00 were used to detect and quantify the signals.

***In silico* analysis**

Expression of INSR and IGF1R in hepatic cancer in patients

In silico assessment of the expression of INSR and IGF1R was performed in hepatocellular carcinoma samples *vs.* normal hepatic tissue in the cancer genome atlas (TCGA) cohort of patients (LIHC – liver hepatocellular carcinoma subgroup) [4]. Analysis and graphical representation were done by the Gepia2 cancer database (http://gepia2.cancer-pku.cn/#index, accessed 18.07.2023).

Survival of hepatic cancer patients

Gepia2 was used to assess the survival of LIHC patients from the TCGA cohort. Patients from the cohort were segregated into 2 groups – group 1 containing 10% of the patients which exhibit the highest expression of either INSR or IGF1R versus group 2 containing the remaining 90% of the patients with lower expression. Kaplan-Meier representation and statistical analysis were done by the Gepia2 database.

Expression of INSR and IGF1R in HCC at different stages

The Human Protein atlas (https://www.proteinatlas.org/ accessed 20.11.2023) was used to assess the expression levels of *INSR* and *IGF1R* at different stages of HCC in LIHC patients from the TCGA cohort. Patients from the cohort were segregated into 3 groups – stage I (n=102), stage II (n=84) and stage III (n=83). Patients with stage IV HCC were not considered as the number of individuals was very low (n=4). Patients with no staging information were also not considered. The human protein atlas was also used to assess the gene expression data for PTEN, IGF1R and INSR in human liver, on a single-cell level (single cell RNA seq database). RNA expression is expressed in normalized transcript per million (nTPM).

GEOdataset analysis of expression of INSR and IGF1R in HCC vs normal liver

The Gene expression omnibus (GEO) datasets (ncbi.nlm.nih.gov/gds) were accessed 17.11.2023. The expression levels of *INSR* and *IGF1R* were determined in 4 different GEOdatasets of HCC patients (GSE14520, n=19, GSE25097, n=243, GSE60502, n=18, GSE64041, n=60) and the fold change in expression between HCC and non-tumoral biopsy was determined for each patient. Expression data are presented as log10 transformed.

GEOdataset	*Type of dataset*	*INSR ID*	*IGF1R ID*	*PMID*
GSE14520	Array	207851_s_at	203627_at	[5]
GSE25097	Array	100148812_TGI_at	100148401_TGI_at	[6]
GSE60502	Array	207851_s_at	203627_at	[7]
GSE64041	Array	8033362	7986359	[8]

GSEA analysis

The GSEA software was used to assess the GSE36376 dataset containing expression analysis from human HCC (240 human HCC samples, segregated between the 20% most expressing

IGF1R or *INSR* and the rest 80% of the samples). Enrichment was calculated based on 1000 permutations (type of permutation – phenotype), using the number of gene ranged at the bottom or the top of the list of genes associated. The sets of genes presenting a FDR q-value < 0.05, were taken as significant.

Statistics

Statistical significance of the results was determined by ANOVA test followed by a Holm-Sidak post-hoc test. Outlier test was performed (ROUT 1%) and outliers were removed. Values were considered significant when compared to CTRL (*) and to PTENKO (~) */~ p≤ 0.05, **/~~ p≤ 0.01, ***/~~~ p≤ 0.001 or ****/~~~~ p≤ 0.0001. Statistical differences between fed and fasted state were represented as follows: ^ p≤ 0.05, ^^ p≤ 0.01, ^^^ p≤ 0.001 or ^^^^ p≤ 0.0001.

Supplementary references

1. Horie, Y., A. Suzuki, E. Kataoka, et al., *Hepatocyte-specific Pten deficiency results in steatohepatitis and hepatocellular carcinomas.* J Clin Invest, 2004. **113**(12): p. 1774-83.
2. Peyrou, M., L. Bourgoin, A.L. Poher, et al., *Hepatic PTEN deficiency improves muscle insulin sensitivity and decreases adiposity in mice.* J Hepatol, 2015. **62**(2): p. 421-9.
3. Monteillet, L., M. Gjorgjieva, M. Silva, et al., *Intracellular lipids are an independent cause of liver injury and chronic kidney disease in non alcoholic fatty liver disease-like context.* Mol Metab, 2018. **16**: p. 100-115.
4. Tomczak, K., P. Czerwińska, and M. Wiznerowicz, *The Cancer Genome Atlas (TCGA): an immeasurable source of knowledge.* Contemp Oncol (Pozn), 2015. **19**(1a): p. A68-77.
5. Roessler, S., H.L. Jia, A. Budhu, et al., *A unique metastasis gene signature enables prediction of tumor relapse in early-stage hepatocellular carcinoma patients.* Cancer Res, 2010. **70**(24): p. 10202-12.
6. Tung, E.K., C.K. Mak, S. Fatima, et al., *Clinicopathological and prognostic significance of serum and tissue Dickkopf-1 levels in human hepatocellular carcinoma.* Liver Int, 2011. **31**(10): p. 1494-504.
7. Wang, Y.H., T.Y. Cheng, T.Y. Chen, et al., *Plasmalemmal Vesicle Associated Protein (PLVAP) as a therapeutic target for treatment of hepatocellular carcinoma.* BMC Cancer, 2014. **14**: p. 815.
8. Makowska, Z., T. Boldanova, D. Adametz, et al., *Gene expression analysis of biopsy samples reveals critical limitations of transcriptome-based molecular classifications of hepatocellular carcinoma.* J Pathol Clin Res, 2016. **2**(2): p. 80-92.
